# BEDwARS: a robust Bayesian approach to bulk gene expression deconvolution with noisy reference signatures

**DOI:** 10.1186/s13059-023-03007-7

**Published:** 2023-08-03

**Authors:** Saba Ghaffari, Kelly J. Bouchonville, Ehsan Saleh, Remington E. Schmidt, Steven M. Offer, Saurabh Sinha

**Affiliations:** 1 Department of Computer Science, University of Illinois at Urbana-Champaign, Thomas M. Siebel Center, 201 N. Goodwin Ave., Urbana, IL USA; 2 Department of Molecular Pharmacology and Experimental Therapeutics, Mayo Clinic, Gonda 19-476, 200 First St. SW, Rochester, MN 55905 USA; 3 Wallace H. Coulter Department of Biomedical Engineering at Georgia Tech and Emory University, Georgia Institute of Technology, 3108 U.A. Whitaker Bldg., 313 Ferst Drive, Atlanta, GA 30332 USA

**Keywords:** Bulk gene expression deconvolution, Single cell RNA-seq, Bayesian inference, Dihydropyridine dehydrogenase deficiency

## Abstract

Differential gene expression in bulk transcriptomics data can reflect change of transcript abundance within a cell type and/or change in the proportions of cell types. Expression deconvolution methods can help differentiate these scenarios. BEDwARS is a Bayesian deconvolution method designed to address differences between reference signatures of cell types and corresponding true signatures underlying bulk transcriptomic profiles. BEDwARS is more robust to noisy reference signatures and outperforms leading in-class methods for estimating cell type proportions and signatures. Application of BEDwARS to dihydropyridine dehydrogenase deficiency identified the possible involvement of ciliopathy and impaired translational control in the etiology of the disorder.

**Supplementary Information:**

The online version contains supplementary material available at 10.1186/s13059-023-03007-7.

## Background

RNA sequencing (RNA-seq) is the cornerstone of regulatory genomics studies. It provides information on changes in gene expression accompanying a biological process and allows for the reconstruction of gene regulatory networks. The widespread adoption and utility notwithstanding, traditional “bulk” RNA-seq technologies offer an incomplete and potentially biased view of expression changes, especially for heterogeneous tissues, since they report gene expression levels aggregated across multiple cell types present in unknown proportions. In data from heterogeneous samples, differential gene expression can reflect a regulated change of transcript abundance within a cell type, a change in the proportion of cell types within the sample, and/or a combination of both phenomena. Differentiating these scenarios is important for inferring mechanisms surrounding biological processes.

Single-cell transcriptomics techniques, such as single-cell RNA-seq (scRNA-seq), can address this issue directly, but are considerably more expensive and time-consuming, limiting the use of these high-resolution assays to fewer samples per biological condition than is afforded by bulk technologies. This practical consideration underlies the need to develop reliable computational methods to deconvolve bulk transcriptomics profiles. Deconvolution methods [1–3] have the potential to reveal cell type-resolution transcriptomes at the low cost and large scale afforded by bulk RNA-seq, allowing greater statistical power in detecting transcriptomic and compositional changes in a biological process.

Deconvolution methods assume that a bulk RNA-seq profile is a weighted mixture of cell type-specific profiles, known as “signatures”, and use statistical techniques to estimate the weights and/or cell type signatures that comprise the bulk profile. While it may be possible to estimate both simultaneously, a more practical approach is to rely on reference signatures (cell type-specific expression profiles from similar biological conditions) to estimate cell type proportions. CIBERSORT [4] and FARDEEP [5] adopt this approach. A similar approach is used by MuSIC [6], SCDC [7], BISQUE [8], and BayesPrism [9], which utilize entire existing scRNA-seq data sets as reference. While these methods demonstrate the potential of this approach, they also highlight challenges that arise due to technical and biological differences between reference signatures and bulk transcriptomic profiles. For instance, reference cell type signatures obtained from scRNA-seq data may be unsuitable for deconvolving bulk RNA-seq data due to a difference in technologies, even if they profile the same biological conditions. Similarly, reference signatures derived from healthy subjects used for deconvolving patient transcriptomics profiles or those from untreated biospecimens used for experimentally perturbed samples may introduce unknown biological biases, leading to errors in deconvolution. Tissue-specific differences in cell type transcriptomes may also lead to such errors.

Recently, comprehensive benchmarking studies have detailed the extent to which biologically and/or technologically mismatched reference signatures can affect the accuracy of deconvolution methods. For instance, Sutton et al. [10] observed a negative impact of biological differences on deconvolution accuracy across all methods tested. Newman et al. [11] propose the use of batch correction to bridge the gap between transcriptomics technologies, while Jew et al. [8] suggest learning a transformation between synthetic bulk profiles generated from a reference scRNA-seq data set and the target bulk data set, which can be used for deconvolution. Sutton et al. [10] propose using reference signatures that are aggregated from multiple sources and technologies, while Wang et al. [6] and Dong et al. [7] utilize heterogeneity across multiple single cell data sets to improve the accuracy of deconvolution. Recently, Chu et al. [9] developed a Bayesian model to infer sample-specific signatures, allowing for technical and biological variation between reference scRNA-seq and bulk expression profiles. Despite these efforts, deconvolution in the face of mismatched references signatures remains an unsolved problem.

In this work, we describe a rigorous Bayesian probabilistic method for bulk expression deconvolution, called BEDwARS (Bayesian Expression Deconvolution with Approximate Reference Signatures), which tackles the problem of signature mismatch from a complementary angle. It does not assume availability of multiple reference signatures, nor does it rely solely on transformations of data prior to deconvolution. Instead, it incorporates the possibility of reference signature mismatch directly into the statistical model used for deconvolution, using the reference to estimate the true cell type signatures underlying the given bulk profiles while simultaneously learning cell type proportions. It assumes that each bulk expression profile is a weighted mixture of cell type-specific profiles (“true signatures”) that are unknown but not very different from given reference signatures. Thus, the reference signatures are used as priors in a Bayesian estimation of true signatures, along with the cell type proportions. Our strategy of jointly inferring both proportions and signatures is a notable departure from the two-step strategy of current methods whereby reference signatures are first “corrected” and then used for deconvolution. It has parallels to so-called full deconvolution methods [12, 13] but its ability to utilize given reference signatures distinguishes it from this genre of methods. Moreover, our technique works with reference cell type signatures from any source and is not limited to scRNA-seq references.

We demonstrate the advantages of BEDwARS through extensive tests on semi-synthetic data sets mimicking human pancreatic islet and brain gene expression data, under varying levels of misalignment between reference and true signatures. We evaluate its ability to recover cell type-specific expression signatures as well as sample-specific cell type compositions in comparison to state-of-the-art reference signature-based deconvolution methods [1]. In these tests, BEDwARS outperforms leading methods such as CIBERSORT, CIBERSORTx, FARDEEP, and BayesPrism in the estimation of cell type proportions. Furthermore, it generally provides more accurate estimates of true cell type signatures compared to RODEO, a state-of-the-art expression deconvolution method that estimates such signatures based on cell type proportions inferred by methods such as CIBERSORTx or FARDEEP. Our evaluations demonstrate the advantage of jointly inferring cell type proportions and cell type-specific signatures while allowing the latter to deviate from pre-determined reference signatures that may not be accurate for the bulk data being studied. Finally, we apply BEDwARS to study the mechanisms underlying pediatric dihydropyridine dehydrogenase (DPD) deficiency, based on new data from induced pluripotent stem (iPS) cell-derived neural organoids.

## Results

### Overview of BEDwARS

**Fig. 1:**
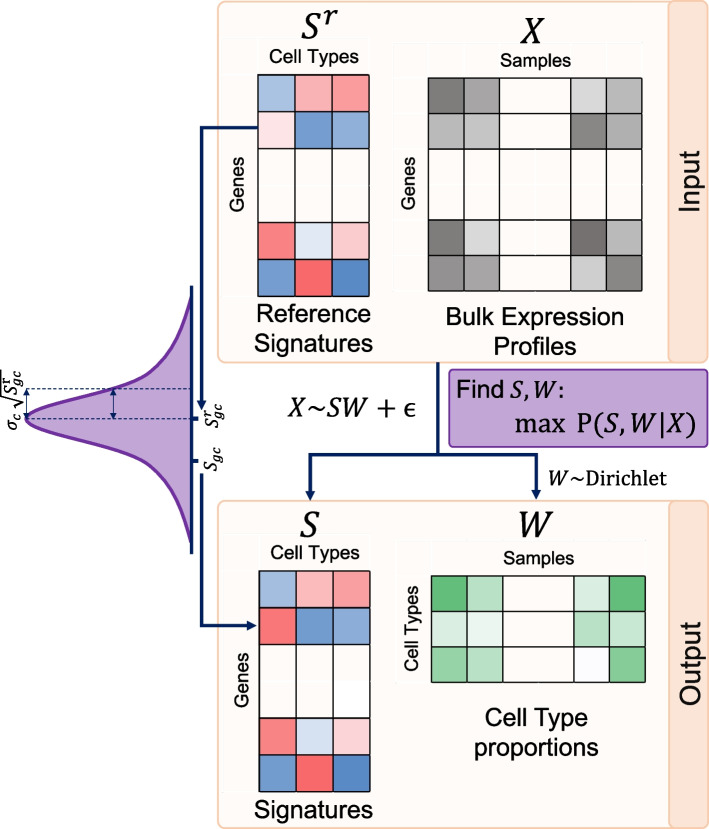
Model outline. BEDwARS takes as input the bulk expression profiles ($X$) as well as the reference signatures of individual cell types (${S}^{r}$). BEDwARS models bulk profiles ($X$) as combinations of “true” but unknown signatures ($S$) of cell types mixed in unknown proportions ($W$) and estimates both $S$ and $W$ from data. The true signatures are assumed to be similar to the reference signatures and differences between them are assumed to be normally distributed with mean zero and variance proportional to the reference gene expression (${S}_{gc}^{r}$). The constant of proportionality is a cell-type specific parameter (${\sigma }_{c}$) that allows for the degree of differences to vary across the cell types. The unknown cell type proportions ($W$) are assumed to follow a Dirichlet distribution. Maximum a posteriori estimation is used to find the cell type proportions and signatures based on the data

BEDwARS is a Bayesian approach to deconvolving bulk expression profiles using reference expression profiles (“signatures”) of the constituent cell types. It is designed to be robust to “noise” in provided reference signatures that may arise due to biological and/or technical differences from the bulk expression profiles. The underlying model assumes, like other deconvolution models, that the bulk expression profile, say ${\varvec{X}}$, of a biological sample is a weighted mixture of cell type-specific signatures, say ${{\varvec{S}}}_{{\varvec{c}}}$ (for each cell type $c$). Loosely speaking, ${\varvec{X}}={\sum }_{c}{w}_{c}{{\varvec{S}}}_{{\varvec{c}}}$, where ${\varvec{X}}$ and ${{\varvec{S}}}_{{\varvec{c}}}$ are $G$-dimensional expression profiles ($G$ is the number of genes) and ${w}_{c}$ is the proportion of cell type $c$ in the sample (Fig. 1). Importantly, the BEDwARS model assumes that a cell type’s signature ${{\varvec{S}}}_{{\varvec{c}}}$, henceforth called the “true signature” of cell type $c$, is similar to but not identical to the available reference signature, say ${{\varvec{S}}}_{{\varvec{c}}}^{\mathbf{r}}$, of that cell type, and must be estimated as part of the deconvolution process. This is the fundamental conceptual difference of BEDwARS from existing approaches. In other words, given a collection of bulk expression profiles $\left\{{\varvec{X}}\right\}$ and a set of reference signatures $\left\{{{\varvec{S}}}_{{\varvec{c}}}^{\mathbf{r}}\right\}$, BEDwARS simultaneously infers the proportions ${\varvec{w}}= {\{w}_{c}\}$ of all cell types $c$ in each sample, and the unknown “true signatures” $\left\{{{\varvec{S}}}_{{\varvec{c}}}\right\}$ of all cell types while maintaining similarity between reference signatures and inferred true signatures.

To understand the model-prescribed relationship between the provided reference signatures and unknown true signatures (Fig. 1), consider the expression of gene $g$ in cell type $c$, as per the true signature (${S}_{gc}$) and reference signature (${S}_{gc}^{\mathrm{r}}$). The model assumes that ${S}_{gc}$ differs from ${S}_{gc}^{\mathrm{r}}$ by an amount that is Gaussian distributed with mean 0 and a gene- and cell type-dependent variance. In particular, the variance of this “noise” term reflecting the difference between the true and reference signature values is proportional to gene’s reference expression (${S}_{gc}^{\mathrm{r}})$ and the constant of proportionality is cell type-dependent. (The noise is modeled on log-transformed expression, see “Methods”.) Thus, the model prefers the true signature value ${S}_{gc}$ to be similar to the reference ${S}_{gc}^{\mathrm{r}}$ but also allows them to diverge, and the divergence can be larger for more highly expressed genes, following a conventional assumption of gene expression models [14]. Furthermore, since biological differences in expression profiles may manifest to different extents in different cell types, the model allows the divergence to be greater for some cell types than for others. The extent of divergence, reflected in the variance term, is learnt from the data.

The above design principles underlie how BEDwARS assigns a probability $\mathrm{Pr}\left({\varvec{X}}| \left\{{{\varvec{S}}}_{{\varvec{c}}}\right\},\boldsymbol{ }{\varvec{w}}\right)$ to the bulk expression profile ${\varvec{X}}$ conditional on a specific estimation of true signatures $\left\{{{\varvec{S}}}_{{\varvec{c}}}\right\}$ and cell type proportions ${\varvec{w}}$, and how it assigns prior probabilities to ${{\varvec{S}}}_{{\varvec{c}}}$ based on reference signature ${{\varvec{S}}}_{{\varvec{c}}}^{\mathbf{r}}$ (see “Methods”). The tool estimates the true signatures and cell type proportions with greatest posterior probability $\mathrm{Pr}(\left\{{{\varvec{S}}}_{{\varvec{c}}}\right\}, {\varvec{w}}|{\varvec{X}})$, using Metropolis Hastings sampling. The calculations include $C+2$ additional parameters (where $C$ is the number of cell types), which are simultaneously optimized. Importantly from a usability perspective, no parameters require hand-tuning and the preset parameters used in the model were the same for all experiments performed in this study (see “Methods” for a more precise description of the model and optimizations).

### BEDwARS deconvolution of human pancreatic islet transcriptomic profiles is robust to mismatched and noisy reference signatures

We assessed the accuracy of bulk expression deconvolution by BEDwARS following benchmarking practices established by recent publications [1, 10] and making use of eight different transcriptomics data sets (Table 1). The overall approach to these evaluations is the following: (1) begin with a single-cell transcriptomics data set with labeled cell types and aggregate the transcriptomes of heterotypic cells to create a “pseudo-bulk” transcriptomic profile, keeping track of the relative proportions of different cell types in the aggregate; repeats of this process result in multiple pseudo-bulk profiles that form the “target” data set to be deconvolved, (2) select a suitable transcriptomics data set representing a biological condition similar to the target data set and wherefrom we can derive an expression profile for each cell type; this is the collection of “reference signatures”, and (3) deconvolve the target data set using the reference signatures and a method of choice, and compare the recovered proportions of different cell types to their true values from step 1. We compared the performance of BEDwARS to leading available tools—BayesPrism [9], FARDEEP [5], CIBERSORT [4], and CIBERSORTx [11]. BayesPrism is among the most recently presented deconvolution methods. FARDEEP and CIBERSORT were the best performing deconvolution tools from the benchmarking study of Cobos et al. [1]. CIBERSORTx was included as it improves upon CIBERSORT by performing batch correction to account for technical differences between the target data set and reference signatures.

**Table 1:** Summary of datasets used for benchmarking

Dataset	Tissue type	Data type	Sequencing protocol	Species
Baron	Pancreatic islet	scRNA-seq	inDrop/CEL-Seq (Illumina Hi-Seq 2500)	Human
Segerstolpe	Pancreatic islet	scRNA-seq	FACS/Smart-Seq2	Human
Enge	Pancreatic tissue	scRNA-seq	FACS/Smart-Seq2	Human
Darmanis	Middle temporal gyrus	scRNA-seq	Smart-Seq	Human
IP	Temporal lobe cortex	Bulk RNA-seq	Illumina Next-Seq	Human
CA	Middle temporal gyrus	snRNA-seq	Smart-Seq2	Human
NG	Prefrontal cortex	snRNA-seq	10X Chromium	Human
MM	Cerebral cortex	Bulk RNA-seq	Illumina Hi-Seq 2000	Mouse

An important aspect of our evaluations was to record how different methods perform with mismatched reference signatures, i.e., those derived from conditions or transcriptomic assays that do not perfectly match those of the target transcriptomic data. Additionally, we tested the impact of artificially “noised” versions of these reference signatures, which we simulated while respecting the general trend observed between these signatures and their corresponding true signatures (see “Methods”; also, we refer below to figures illustrating noisy signatures).

**Fig. 2:**
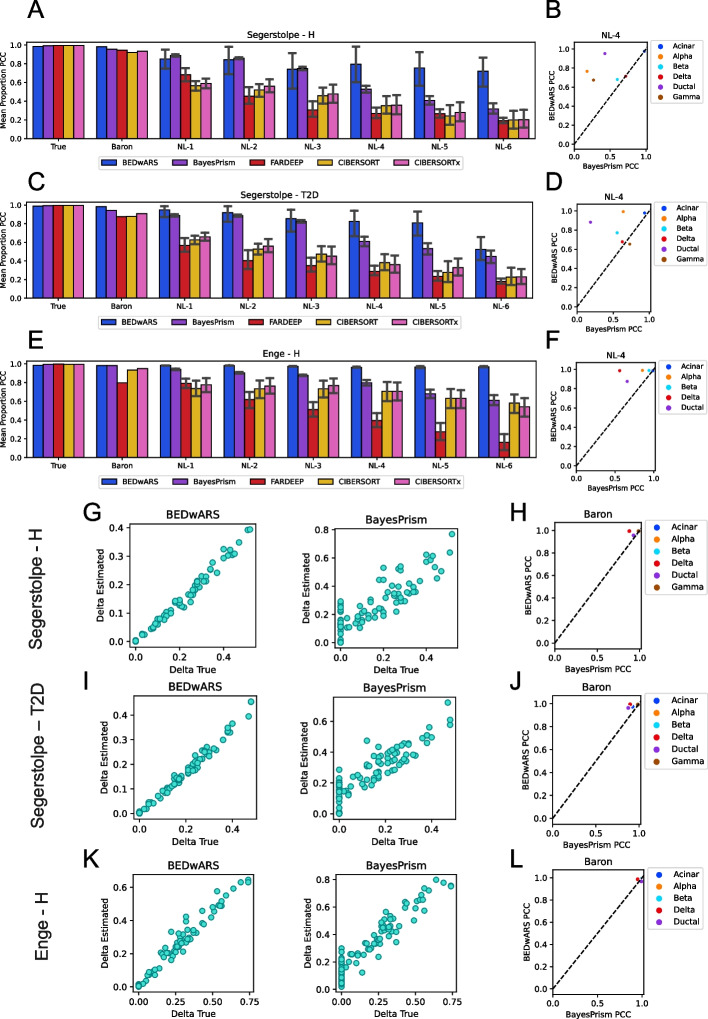
Evaluation of cell type proportion estimation in pancreatic transcriptomic profiles. **A**, **C**, **E** Pearson correlation coefficient (PCC) between true and estimated cell type proportions in 100 pseudo-bulk samples, averaged over cell types, is shown for different deconvolution methods. Results are shown for the Segerstolpe-H (**A**), Segerstolpe-T2D (**C**), and Enge-H (**E**) datasets. Category labels of bar charts indicate the reference signature, with “True” indicating the true underlying signature that is normally not available during deconvolution, “Baron” indicating the Baron signatures, and “NL-x” indicating Baron signatures with noise added at level x. For “NL-x”, results shown are mean with 95% confidence interval from evaluations using 11 variants of the Baron signature with noise added at level x. **B**, **D**, **F**, **H**, **J**, **L** PCC for each cell type separately is compared between the two best methods for respective datasets, when using NL-4 signatures (**B**, **D**, **F**) or Baron signatures (**H**, **J**, **L**). **G**, **I**, **K** Estimated and true proportions in the 100 pseudo-bulk profiles are directly compared, for a single cell type from each dataset, and for the two best methods for that dataset. BEDwARS performance is more robust to noise than the other methods in all datasets. All methods have comparable performance when the true signature is used. For the Baron signature, the performance of BEDwARS is similar to other methods, with a noticeable improvement for Segerstolpe-T2D dataset. BEDwARS provides better estimates for almost all cell types in the NL-4 evaluations and for at least one cell type with the Baron signatures

For our first evaluations, we used single-cell RNA-seq data on human pancreatic islets from healthy subjects [15] to generate 100 pseudo-bulk profiles from weighted mixtures of cells of six pre-labeled types—alpha, beta, gamma, delta, acinar, and ductal. We adopted the procedures of Cobos et al. [1] for data processing and generation of mixtures. Deconvolution of this target data set (called “Segerstolpe-H”) was set to be performed using reference signatures constructed from the inDrop scRNA-seq data of pancreatic islet samples from Baron et al. [16]. (Single cell transcriptomic profiles of cells of a type were averaged to obtain the reference signature of that cell type.) Note that the reference signatures and the target data set (pseudo-bulk profiles) are derived from different sequencing platforms (Table 1); this is one way of mimicking the technical differences between transcriptomics profiles that are often encountered in real-world deconvolution problems. Additional file 1: Fig. S1 shows the relationship between reference and true signatures, suggesting a high level of concordance despite the technical differences. Each of the five evaluated methods—BEDwARS, BayesPrism, FARDEEP, CIBERSORT, and CIBERSORTx—was used to infer cell type proportions in each pseudo-bulk profile and we calculated, for each cell type, the Pearson correlation coefficient (PCC) between true and predicted proportions across the 100 pseudo-bulk profiles. Figure 2A (group “Baron”) shows that BEDwARS makes marginally more accurate estimates, indicated by average PCC over the six cell types, though all five methods proved highly accurate in this evaluation. Figure 2H shows this comparison for each cell type separately, for the top two methods—BEDwARS and BayesPrism, revealing that the difference in performance is mainly for the delta cell type (PCC 0.99 vs 0.88), as seen more clearly in Fig. 2G. (See Additional file 1: Fig. S2 for similar comparisons for all cell types and methods.) The improved accuracy of BEDwARS estimates is also borne out when using alternative metrics of comparison—mean absolute error (MAE) or root mean squared error (RMSE), rather than PCC—between true and estimated proportions (Additional file 1: Fig. S3A, B). For instance, we observed BayesPrism to underestimate beta and acinar proportions by nearly a factor of two and overestimate delta and ductal proportions in samples depleted of these cell types (Additional file 1: Fig. S2), leading to a higher RMSE and MAE compared to BEDwARS (Additional file 1: Fig. S3A, B).

To test the effect of noisy reference signatures, we created six sets of perturbed versions of the above-mentioned Baron reference signature, representing different levels of noise and named “NL-1”, “NL-2”, etc., using randomization to create 11 such signatures in each set. Noise was introduced as described in “Methods”, maintaining the overall trend with the true signature and with a greater noise level inducing greater variance among genes with similar expression values in the true signature. The extent of injected noise is illustrated, for the lowest and highest levels NL-1 and NL-6, in Additional file 1: Fig. S1; as seen in Additional file 1: Fig. S4A, increasing noise levels result in progressively decreasing correlation coefficient between the reference and true signatures. As shown in Fig. 2A, accuracy of cell type proportion estimation degrades with increasing noise levels (groups NL-1, … NL-6), but to a lesser extent for BEDwARS than the other methods. Performances of BEDwARS and BayesPrism are similar and better than other methods for lower noise levels (NL-1,-2,-3), while BEDwARS is significantly better than all other methods for higher noise levels (NL-4,-5,-6). Figure 2B shows that BEDwARS is substantially better than BayesPrism at the noise level (NL-4), inferring more accurate proportions for four out of six cell types. Additional file 1: Fig. S5 shows that BEDwARS has greater or almost equal accuracy compared to BayesPrism (the second-best method) for all cell types and noise levels except for delta and beta cell types in NL-1 and NL-3. Notably, at all noise levels, BEDwARS has the least RMSE and MAE between estimated and true proportions (Additional file 1: Fig. S3A, B).

Next, we created a new target data set (called “Segerstople-T2D”) comprising pseudo-bulk mixtures derived from the same study as in Fig. 2A (human pancreatic islets [15]) but representing patients with type II diabetes (T2D). (See Additional file 1: Figs. S4B and S6.) Mirroring trends in Segerstolpe-H evaluations, BEDwARS and BayesPrism perform similarly and significantly better than other methods for lower noise levels (NL-1,-2,-3). BEDwARS stays the best method for higher noise levels, with significant improvements over BayesPrism for NL-4,-5 (Fig. 2C). The significant gap between BEDwARS and BayesPrism at the noise level NL-4 is due to superior performance for three cell types and almost equal performance for the rest of them (Fig. 2D). (See Additional file 1: Fig. S7 for more complete comparisons.) In fact, the performance gap in the absence of noise (Fig. 2C, group “Baron”) was slightly more than in Segerstolpe-H evaluations, with the larger differences seen for the delta and ductal cell types (Fig. 2J, I). Additional file 1: Fig. S8 shows a detailed comparison with all methods for all cell types, revealing for instance that CIBERSORT and CIBERSORTx (collectively called CIBERSORT(x)) underestimate acinar cell type proportions by nearly an order of magnitude and BayesPrism estimations are off-target by a factor of four, while BEDwARS estimates are close to the true values. BEDwARS has a clear advantage over all methods here, indicated by its exhibiting the least RMSE and MAE between estimated and true proportions at no noise (group “Baron”) and all noise levels (see Additional file 1: Fig. S3C, D).

We repeated the above evaluations with a different target data set—pseudo-bulk mixtures generated using scRNA-seq data from Enge et al. [17], representing human pancreatic tissue from healthy subjects (“Enge-H data set”). This is similar to the above tests in that the reference signatures (from Baron et al. [16]) and target profiles represent different technologies (Table 1). (Also see Additional file 1: Figs. S4C and S9.) Performance comparisons yielded similar trends (Fig. 2E)—BEDwARS showed marginal improvement over BayesPrism with the Baron signatures (Fig. 2K, L), but now the gap with FARDEEP was larger (average PCC of 0.98 vs 0.8) (Additional file 1: Fig. S10); see Additional file 1: Fig. S3E, F for MAE and RMSE metrics. BEDwARS also showed a remarkable robustness to increasing noise levels, with progressively greater improvements over the other methods. A direct comparison with the second-best performing method (BayesPrism) at noise level NL-4 (Fig. 2F) shows that the largest performance gap is for delta cell type. (Also see Additional file 1: Fig. S11 for comparisons to BayesPrism at varying noise levels.)

We then performed a closer comparison for the performance of BEDwARS with other methods in recovering rare cell type population. In all pancreatic datasets, BEDwARS estimates the rare cell type proportions better than other methods (see Additional file 1 Supplementary Note 1A, Figs. S36-S41, Table S11). In another evaluation, we used heterogeneous data sets comprising pseudo-bulk profiles generated from two different sources and observed that BEDwARS performance can occasionally deteriorate in such scenarios as it assumes that the bulk profiles share their underlying signatures (see Additional file 1 Supplementary Note 1B, Fig. S42). We also examined the effect of varying numbers of bulk profiles and based on the results we recommend users to use datasets with the size at least four times the number of cell types to be deconvolved (see Additional file 1 Supplementary Note 1C, Fig. S43).

In summary, BEDwARS was found to provide more accurate estimates of cell type proportion compared to four leading methods, across a range of benchmarking conditions representing varying levels and sources of divergence between the true cell type signatures underlying the target data set and the provided reference signatures. (This was observed not only with the PCC but also alternative evaluation metrics such as MAE and RMSE.) Notably, all methods yielded near-perfect estimates of proportions when provided the true signatures, in all settings, indicating that the challenge in accurate deconvolution arises mainly from signature mismatch and noise.

### BEDwARS accurately estimates cell type signatures from noisy references

The principle underlying robust deconvolution by BEDwARS is to jointly estimate proportions as well as cell type signatures, allowing the latter to diverge from the reference. It is natural to ask, then, if the estimated signatures are indeed accurate. This can be assessed by comparing the BEDwARS-estimated cell type signature to the corresponding true signature from the target data set, using correlation coefficients. An alternative strategy to reconstructing the true signatures is to estimate cell type proportions in the target data set (as above) and use this information to re-estimate the true signatures. For this last step, we chose RODEO, a leading deconvolution method based on robust linear regression that infers cell type-specific signatures underlying a given bulk transcriptomics data set, given their cell type proportions in each sample. RODEO has been shown to be more accurate compared to other existing methods and to be robust to noise in the cell type proportions provided to it. We thus compared signature estimation accuracy of BEDwARS and RODEO, with the latter using cell type proportions estimated using BayesPrism, FARDEEP, CIBERSORT, or CIBERSORTx (in four separate runs). We also examined the accuracy of RODEO when provided cell type proportions from BEDwARS deconvolution. The correlation between the true signatures and reference signatures was used as a baseline.

**Fig. 3:**
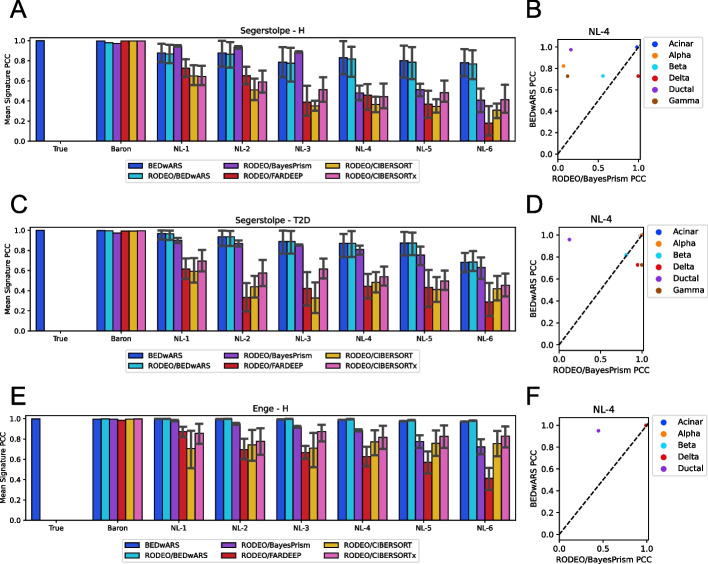
Evaluation of cell type signature estimation using pancreatic transcriptomic profiles. **A**, **C**, **E** Pearson correlation coefficient (PCC) between true and estimated cell type signatures, averaged over cell types, is shown for different deconvolution methods. Signatures were estimated by deconvolving 100 pseudo-bulk samples generated from Segerstolpe-H (**A**), Segerstolpe-T2D (**C**), and Enge (**E**) datasets. Performance of BEDwARS is compared with RODEO provided with cell type proportion estimates obtained using BayesPrism, CIBERSORT, CIBERSORTx, FARDEEP, or BEDwARS. Category labels of bar charts indicate the reference signature used. Category label “True” indicates that the true signatures were provided as reference to BEDwARS; no comparisons are made to RODEO in this case, rather this setting was used to assess if BEDwARS, which allows the estimated signature to deviate from the given reference, reports back an estimated signature similar to the true signature. BEDwARS is more robust to the increasing noise levels in recovering the true signatures in all datasets. RODEO provided with BEDwARS-estimated cell type proportions performs as well as BEDwARS, suggesting that accurate proportion prediction by BEDwARS is key to accurate signature estimation. **B**, **D**, **F** PCC for each cell type separately is compared between BEDwARS and its closest competitor (excluding RODEO/BEDwARS) for respective datasets, when using NL-4 signatures. In this setting, the PCC is substantially higher for BEDwARS-estimated signatures of ductal cell type than its competitor, for all datasets

The above evaluations were performed on each of the three benchmarks with human pancreatic data and revealed a few clear trends (Fig. 3). First, BEDwARS and/or RODEO/BayesPrism estimate significantly more accurate signatures when provided with noisy reference signatures, compared to the other three methods. For instance, on the Segerstolpe-T2D target data set (Fig. 3C, D), BEDwARS and RODEO/BayesPrism have similar performance, which is better than other alternatives at all noise levels. On the Segerstolpe-H data set (Fig. 3A), RODEO/BayesPrism performs marginally better than BEDwARS at lower noise levels (NL-1,-2,-3) whereas BEDwARS is significantly better at higher noise levels (NL-4,-5,-6) (Fig. 3B). On the Enge-H dataset, BEDwARS is the top performing method at all noise levels and its performance gap with BayesPrism is larger at higher noise levels (Fig. 3E, F). Cell type level performance comparison reaffirms the advantage of BEDwARS for signature estimation at higher noise levels (Additional file 1: Figs. S12-S14).

A second trend we noted was that all methods inferred accurate signatures when provided with the Baron signatures without noise (Fig. 3A, C, E, group Baron). Thirdly, we realized that poor signature estimation was tied mainly to errors in the proportion estimation (the first deconvolution step), as evidenced by the fact that RODEO recovers equally accurate signatures as BEDwARS if provided with the more accurate cell type proportions from BEDwARS; this is true regardless of the noise levels. Trends seen here were confirmed when using RMSE instead of correlation as the metric for signature comparison (Additional file 1: Fig. S15).

The results of this and the previous section together demonstrate the value of jointly estimating cell type proportions and signatures when deconvolving bulk profiles using noisy or mismatched reference signatures. Indeed, when true signatures underlying the target data set are known accurately, all methods recover proportions accurately (Fig. 2A, C, E; group True), and when proportions are estimated accurately using BEDwARS, RODEO can also provide equally accurate signatures.

### Robust deconvolution of brain transcriptomic profiles with BEDwARS

The next set of evaluations were performed following the recent benchmarking study of Sutton et al. [10], where scRNA-seq data from middle temporal gyrus in human brain of [18] were used to generate the target data set. Using their methodology, we generated 100 pseudo-bulk profiles as weighted mixtures of the three most frequent cell types in the scRNA-seq data—neurons, astrocytes, and oligodendrocytes. As above, the proportions and true signatures used here were recorded as ground truths of the benchmark. In our first evaluations on these data, the reference signatures used were bulk RNA-seq profiles of immunopurified (IP) cells from the human brain [19] (see “Methods”); this is the “IP” signature. Noisy versions of this signature were also tested. (See Additional file 1: Figs. S4D and S16 for illustrations of how the IP signature and its noisy versions relate to the true signature.)

**Fig. 4:**
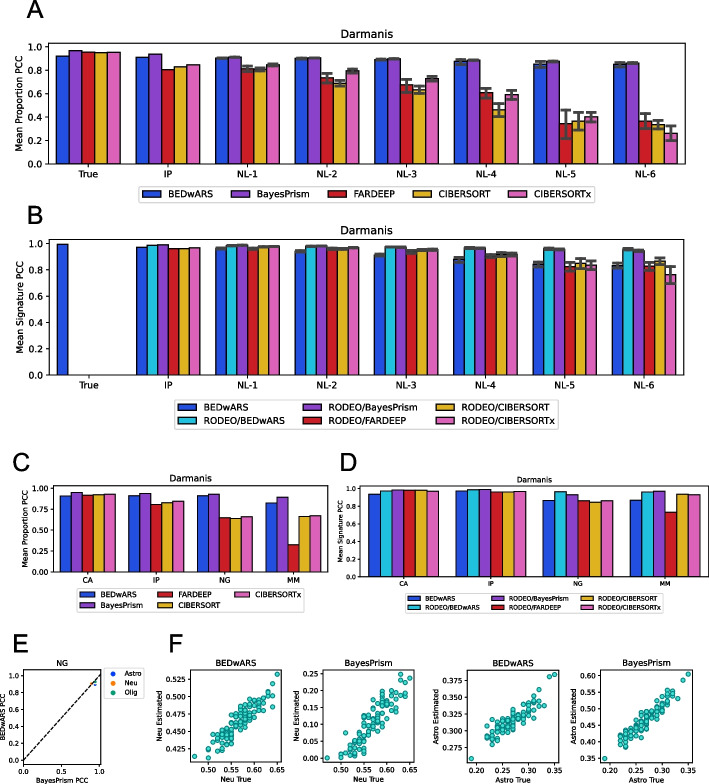
Evaluation of cell type proportion and signature estimation from brain transcriptomic profiles. **A**, **B** Pearson correlation coefficient (PCC) computed between the estimated and true cell type proportions (**A**) or cell type signatures (**B**), averaged over cell types, when deconvolving 100 pseudo-bulk samples generated from Darmanis dataset. Category labels of bar charts indicate the reference signature used. BEDwARS and BayesPrism are similar and have higher PCC than the other methods in the estimation of cell type proportions for the IP signature and its noisy versions (NL-x), with the performance gap increasing as the noise level increases. For estimation of cell type signatures, RODEO provided with BEDwARS or BayesPrism-estimated proportions (RODEO/BEDwARS, RODEO/BayesPrism) outperform other methods including BEDwARS. **C**, **D** Average PCC between estimated and true cell type proportions (**C**) or signatures (**D**), using the IP signatures (same as in **A**, **B**) as well as the CA, NG, MM signatures. All methods perform comparably for proportion estimation when using the CA signature but BEDwARS exhibits better performance when the reference signature is more diverged from the true signature, such as NG (different region of human brain) and MM (mouse brain). All methods show comparable performance in signature estimation when provided the CA and IP references signatures, but RODEO provided with BEDwARS- or BayesPrism-estimated proportions exhibits superior performance for the more diverged reference signatures (NG and MM). **E** PCC for each cell type separately is compared between the two best methods (BEDwARS and BayesPrism) when using NG signatures. Both methods perform equally well evaluated by PCC criterion. **F** Estimated and true proportions in the 100 pseudo-bulk profiles are directly compared, for neurons (NEU) and astrocytes (ASTRO), for the two best methods when using the NG signatures. BEDwARS estimates are considerably more accurate in magnitude than BayesPrism estimates

As shown in Fig. 4A, BEDwARS and BayesPrism provide more accurate estimates of cell type proportions in the brain data set, as compared to the three other methods. This is true even with the no-noise reference signatures (group “IP”, BEDwARS/BayesPrism correlation 0.91/0.94 vs. CIBERSORTx correlation 0.84). Their deconvolution accuracy remains stable (between 0.91 and 0.85) at the wide range of noise levels, while other methods see their accuracy drop from ~ 0.83 (at no noise) to ~ 0.32 at the highest noise level; this trend is also seen with alternative metrics such as MAE and RMSE (Additional file 1: Fig. S17A, B). A closer examination (Additional file 1: Fig. S19) reveals that with the IP signature the oligodendrocytes cell type is the primary reason for performance deterioration in CIBERSORTx (BEDwARS correlation 0.94 vs. CIBERSORTx correlation 0.81), which underestimates the true proportions by nearly four-fold. (Also see Additional file 1: Figs. S18 and S19.) Evaluation of signature estimation accuracy by PCC criterion (Fig. 4B) suggests that all methods are capable of recovering the true underlying signatures for this data set, although at higher noise levels RODEO using BEDwARS- or BayesPrism-estimated cell type proportions (RODEO/BEDwARS, RODEO/BayesPrism) clearly outperform others. (Also see Additional file 1: Fig. S20.) This is reaffirmed by evaluations with the RMSE criterion (Additional file 1: Fig. S15D) and provides further support for the advantage of BEDwARS and BayesPrism in terms of proportion estimation.

Next, we evaluated brain transcriptome deconvolution with three additional reference signatures that were considered by Sutton et al. [10]. These include a signature obtained from bulk RNA-seq profiles of immune-purified cells from mouse brain tissue [20] (“MM”- Mus Musculus), one obtained from Human Cell Atlas containing single-nucleus RNA-seq of adult human middle temporal gyrus [21] (“CA”-Cell Atlas), and one signature from single-nucleus expression profiles of adult human prefrontal cortex in control samples [22] (“NG”- Nagy et al.). Additional file 1: Figs. S21 and S22 show that the MM and NG signatures are more diverged from the true signatures (than are IP signatures), while the CA signatures are more similar to the true signatures. As shown in Fig. 4C, with the NG and MM reference signatures (more mismatch), the BEDwARS- and BayesPrism-estimated proportions (Additional file 1: Fig. S24A) are clearly more accurate than CIBERSORTx and FARDEEP. Evaluations with MAE and RMSE metrics (Additional file 1: Fig. S17C,D) confirm the advantage of BEDwARS in this benchmark, but only for the NG signature; the MM signature yields comparable accuracy across methods by these alternative metrics. Notably, when using NG signatures, neuron proportions are severely underestimated by BayesPrism (Fig. 4F, Additional file 1: Fig. S23) while astrocyte proportions are overestimated by a factor of 1.5, even though PCC values for either cell type are similar between BEDwARS and BayesPrism (Fig. 4E).

Evaluation of signature estimation (Fig. 4D) suggests that for the CA and IP signatures (more matched with true signatures) all methods perform equally well, while for the NG and MM signatures (more mismatched), RODEO using BEDwARS- or BayesPrism-estimated proportions has better performance than others. (Also see Additional file 1: Figs. S15E and S24B.) For deconvolution with MM signatures, we observed (Additional file 1: Fig. S25) that oligodendrocyte proportions are poorly estimated by all methods but BEDwARS and BayesPrism estimates are well correlated with true proportions. This suggests that a cell type signature estimation method such as RODEO can benefit from proportion estimates that are accurate in relative if not absolute terms. Evaluations using the CA reference signature revealed similar performance by all evaluated methods, both for proportion estimation (Additional file 1: Fig. S26) and for signature estimation, with correlation values of ~ 0.9 or greater (Fig. 4C, D), highlighting the importance of matched reference signatures for the deconvolution task.

We also compared BEDwARS with the recently reported tool SCADIE [23], which, like RODEO, relies on initial estimates of cell type proportions provided by any existing deconvolution method and iteratively refines the cell type signatures and proportions. Our evaluations demonstrate that the performance of SCADIE in both proportion and signature estimation is strongly dependent on the quality of cell type proportions used in the initialization. In particular, we found that when using reference signatures that are more diverged from true signatures, SCADIE initialized with CIBERSORTx- or FARDEEP-estimated proportions performs significantly worse than when initialized with BEDwARS-estimated and comparably to BEDwARS (for proportion estimation) and RODEO/BEDwARS (for signature estimation). (See Additional file 1: Figs. S27-S29).

In summary, extensive comparative evaluations on a published set of benchmarks involving brain transcriptomics data reaffirmed the conclusions drawn from pancreatic islet benchmarks, that BEDwARS is capable of robust proportion estimation in the face of noisy and mismatched signatures and such proportions can then be the basis of more accurate signature estimation as well.

### Application of BEDwARS to characterize the cell type-specific regulomes of DPD-deficient patients

In this section, we present a case study in the use of single cell and bulk transcriptomics to characterize molecular mechanisms underlying a rare disorder. Dihydropyridine dehydrogenase (DPD) deficiency is caused by deleterious germline variants within the *DPYD* gene and typically presents as a pharmacogenomic condition, in which patients are at significantly higher risk of severe adverse events when treated with the commonly used chemotherapeutic 5-fluorouracil (5-FU) [24]. DPD deficiency has also been linked to rare inborn error of metabolism that is accompanied by neurological disorders of varying degrees of severity in children [25, 26]. The penetrance of the pediatric condition within individuals with DPD deficiency is very low. For the purposes of this manuscript, we will refer to this condition as “pediatric DPD deficiency” to distinguish it from the pharmacogenomic disorder or the generalized reduction in DPD function. While the biochemistry surrounding DPD is well characterized, there is extremely limited information pertaining to how DPD deficiency could contribute to the clinical presentation of neurologic and metabolic conditions in affected children.

The analyses presented in this manuscript represent a subset of a larger clinical study designed to characterize the developmental and biochemical pathways that are altered in pediatric DPD deficiency with the goals of gaining a better understanding of the disease etiology as well as identifying potential therapeutic approaches to improve quality of life for affected patients. For the overall study, fibroblasts were obtained from affected individuals, non-affected family members, and unrelated controls. Fibroblasts were reprogrammed into induced pluripotent stem cells (iPS cells), which were subsequently used to derive neural organoids. At least 3 independent iPS clones were generated from each subject.

For the present study, RNA-seq was performed on 72 brain organoids from three patients with pediatric DPD deficiency (referred to as DPD1, DPD3, and DPD6) and on 48 organoids from two non-affected subjects (DPD2 and DPD4). ScRNA-seq profiling was also performed for three organoids from patient DPD1 and for three organoids from the non-affected subject DPD4. For purposes of cross-technology calibration, we ensured that eight of the organoids profiled using bulk RNA-seq in each group (patient or non-affected) were generated and cultured in parallel with the three organoids used for scRNA-seq. We will refer to these bulk-profiled organoids as “semi-matched” bulk samples below.

**Fig. 5:**
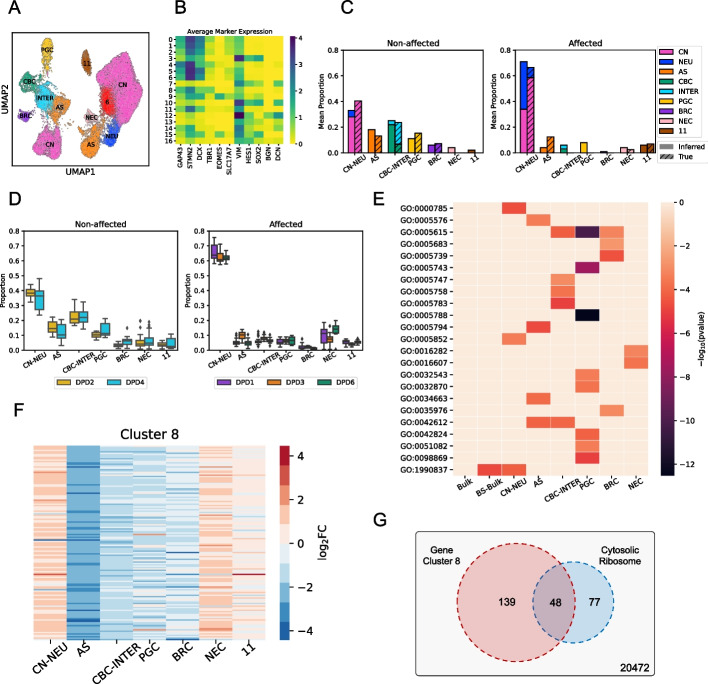
Cell type-specific characterization of transcriptomic differences between organoids from DPD deficiency affected and non-affected subjects using BEDwARS deconvolution of bulk RNA-seq data. **A** UMAP plot of processed cells clustered into 17 groups. Cells on the right represent the affected patient and cells on the left represented the non-affected subject. **B** Average expression of 11 marker genes in cells of each cluster indexed by numbers. These markers were used for cell type assignment. **C** Comparison between the average inferred proportions (plain) from eight bulk samples of an affected and a non-affected subject and the average of “true” proportions (diagonal striped) derived from the semi-matched single cell data on three organoids for the same subject. **D** Inferred proportions of different cell types obtained by BEDwARS deconvolution of bulk RNA-seq data from organoids derived from two non-affected subjects and three affected patients. CN-NEU sum of inferred proportions of CN and NEU, CBC-INTER sum of inferred proportions of CBC and INTER. **E** Negative logarithm (base 10) of the *p*-value ($-{\mathrm{log}}_{10}(\mathrm{pvalue})$) of hypergeometric tests of Gene Ontology (GO) term enrichments in the top 200 differentially expressed genes (DEGs) from bulk samples (72 affected vs 48 non-affected, “Bulk”), bootstrapped bulk profiles derived from the single cell data (100 affected vs 100 non-affected, “BS-Bulk”) and the cell type-specific bulk expression derived from BEDwARS deconvolution (72 affected vs 48 non-affected, “CN-NEU”, “AS”, “CBC-INTER”, “PGC”, “BRC”, “NEC”). None of the GO terms enriched in the top 200 DEGs of cell type-specific profiles are enriched in the top 200 DEGs derived from the bulk expression profiles. See Additional file 1: Table S3 for information on GO terms. **F** Logarithm (base 2) of the fold-change (${\mathrm{log}}_{2}\mathrm{FC}$) of expression of the 139 genes grouped into cluster 8 based on their pattern of differential expression in different cell types. **G** The 139 genes of cluster 8 are highly enriched in the GO term “cytosolic ribosome” (hypergeometric test *p*-value $2\times {10}^{-85}$)

To deconvolve the bulk RNA-seq profiles into cell type-specific components, we first generated reference signatures using the single-cell data. Single-cell profiles from the affected and non-affected subjects were processed together to obtain ~29,707 cells that segregated into 17 clusters that potentially represented different cell types and states (Fig. 5A). To identify cell types represented by these clusters, we utilized a multi-pronged strategy based on work by Tanaka et al. [27]. The average expression of neuronal markers (STMN2, GAP43, DCX) and early neurogenesis genes (VIM, HES1, SOX2) was used to discriminate neuronal from non-neuronal clusters (Fig. 5B, Additional file 1: Fig. S30). Further resolution was achieved through the consideration of additional known cell type markers, as well as statistically identified marker genes, enrichment of cell type-related Gene Ontology terms in these markers and overlaps with similarly obtained marker sets from Tanaka et al. (see “Methods”) (Additional file 1: Fig. S31). Using this approach, we were able to assign cell types to 15 of the 17 clusters (Additional file 1: Table S1). Notably, cortical neurons and astrocytes were the only cell types with representation in the affected and non-affected samples. Reference signatures were then obtained as average gene expression profile of each cell type found in the non-affected individual (astrocytes (AS), cortical neurons (CN), progenitor cells (PGC), cilia-bearing cells (CBC), intermediate (INTER), BMP-related cells (BRC)), as well as three cell types in the affected individual (neurons (NEU), neuroepithelial cells (NEC) and cluster-11); in some cases, multiple clusters were mapped to the same cell type in this step (see “Methods”).

We next performed deconvolution of bulk RNA-seq profiles in each group (i.e., affected and non-affected) separately, using BEDwARS with the above-mentioned references signatures of nine cell types. (Bulk profiles for each group were first batch corrected to match the pseudo-bulk profiles generated from single cell data for the respective group, see “Methods” and Additional file 1: Fig. S32.) As an internal control, we first compared estimated cell type proportions in the eight semi-matched bulk samples in each group to those in the scRNA-seq samples of the same individual and found the deconvolution to successfully recover the proportions of dominant cell types (Fig. 5C). In both groups, the sum of inferred proportions of cortical neurons (CN) and neurons (NEU) matched the corresponding sum in the single cell data. However, the proportions of these two individual cell types could not be accurately resolved due to the similarity in the signatures (see Additional file 1: Fig. S33). Similar observations were made for the cilia-bearing cells (CBC) and intermediate (INTER) cell type proportions, with their sum matching between bulk-deconvolved and single cell data. Apart from these four cell types, any other cell type with either the inferred or true proportion above 5% (AS, PGC, BRC in non-affected and PGC, cluster-11 in affected) was deconvolved accurately. The only exception to this trend was the AS cell type in the affected samples, where the true proportion from single cell data, roughly 10%, was underestimated at ~4%, at the expense of an over-prediction of PGC proportion. Overall, this exercise confirmed our ability to deconvolve cell type proportions in bulk RNA-seq from organoids, leading us to apply the same procedure to the entire data set.

We next deconvolved the 48 and 72 bulk profiles from two non-affected and three affected subjects respectively, using the same signatures and procedure as above. Estimated proportions of almost every cell type (and in two cases, sums over cell type pairs – CN+NEU, CBC+INTER) were consistent among subjects within the same group (Fig. 5D). The CN+NEU proportion is noticeably higher in the affected subjects, consistent with the limited single cell data (Fig. 5C), while CBC+INTER proportion is higher in the non-affected subjects. Ciliated cells (i.e., CBCs) are involved in extracellular signal transduction that is critical for patterning and morphogenesis during neural development [28, 29]. Disruptions to the processes facilitated by ciliated cells has been shown to contribute to both neurodevelopmental and degenerative diseases [30]. The reduced number of CBC populations within organoids derived from affected individuals is suggestive that ciliopathies might be linked to DPD deficiency and contribute to the observed clinical presentation of pediatric DPD deficiency, warranting further study of this novel observation.

Deconvolution with BEDwARS also allowed us to examine cell type-resolved components of each bulk transcriptomic profile, obtained by multiplying the cell type’s inferred proportion with the respective inferred signature. We could thus compare gene expression between the two groups of samples (affected versus non-affected) in a cell type-specific manner, with the large numbers of bulk RNA-seq samples (72 and 48 in the two groups) providing high statistical power and the multiplicity of subjects in each group offering a more diverse representation than possible with the limited single cell data. We derived the genes most differentially expressed (DE) between groups, for each cell type separately (Additional file 2: Table S2), and performed Gene Ontology (GO) enrichment tests for the top 200 genes to characterize their biological functions (Fig. 5E, Additional file 1: Table S3 and Additional file 3: Table S4), see “Methods”. None of the significant GO terms (FDR < 0.05), except for one, obtained from this cell type-specific analysis were significantly enriched in the top 200 DE genes derived from bulk profiles or from bootstrapped samples of the single cell data. This demonstrates that the deconvolution approach likely helped reveal latent patterns of gene expression changes within specific cell populations that could not be observed within bulk data or in a limited sampling strategy (i.e., small number of organoids from fewer subjects) common to scRNA-seq analyses.

For example, chronic fatigue and metabolic dysfunction, consistent with mitochondrial disorders, have been previously reported in subjects with DPD deficiency [31]. However, it is unclear if mitochondrial disorder is a shared feature of pediatric DPD deficiency [32]. Mitochondria-related GO terms were identified in the deconvolved expression data for PGC, CBC-INTER, and BRC cell types (Fig. 5E and Additional file 1: Table S3 and Additional file 3: Table S4, suggesting that changes in the expression of mitochondrial genes within these compartments might be relevant to the disease etiology. As another example of latent features potentially identified by this analysis, dysfunctions in protein synthesis and folding (e.g., endoplasmic reticulum, ER, dysfunction) have been suggested to contribute to numerous neurological disorders; however, the etiology and/or pathogenesis have not been fully elucidated [33]. Terms related to ER and translation initiation were significantly enriched in CBC-INTER, PGC, CN-NEU, NEC, and AS clusters (Fig. 5E), suggesting that changes in protein translation and folding might contribute to clinical presentation of pediatric DPD deficiency.

In a complementary analysis, we clustered genes based on their patterns of differential expression across all nine cell types (Additional file 1: Fig. S34), obtaining 10 major clusters of 39–5107 genes (Additional file 4: Table S5), with similar GO term associations as above (Additional file 5: Table S6). One of these clusters (cluster 8, Fig. 5F) shows a pattern in which genes are up-regulated in CN+NEU and NEC but downregulated in AS, PGC, and CBC+INTER cell types, in affected subjects compared to non-affected subjects. Genes showing this pattern of expression were enriched for those associated with the cytosolic ribosome (i.e., “free” ribosomes, Fig. 5G; FDR = 5 × 10^–83^). Cells of the nervous system, in particular neuronal cells, rely on localized translation of gene products via cytosolic ribosomes [34]. This pseudo-compartmentalized translation within neural cells has been shown to create spatial variability in protein expression that is critical for neural development, function, and plasticity [35]. Disruptions to localized translation have been linked to various neurodevelopmental and neurodegenerative disorders [36, 37]. Combined with the results linking ER/translation GO terms in these same cells, these findings indicate that dysregulation of translation and protein folding, whether at the ER and/or at distal sites, might contribute to the clinical presentation of pediatric DPD deficiency.

## Discussion

We present here a Bayesian approach to the deconvolution of bulk expression profiles especially designed to address potential differences between reference cell type signatures and the true (but unknown) cell type signatures underlying the bulk profiles, a common challenge in deconvolution. One might expect that a Bayesian deconvolution approach that allows for noisy signatures as part of its model will not perform best if the reference signatures are in fact very similar to the true signatures underlying the bulk data, i.e., when the anticipated noise is not there. However, we noted that our model’s performance is better than or competitive with the other approaches in most of the benchmarks in this study, including those where the signature differences were the smallest. This suggests that in such cases, the model learns to perturb the reference signature to a lesser extent based on the data. We note that BayesPrism also allows for adjustments to single cell-derived reference signatures, but not in a cell type-specific manner as is done in BEDwARS. Therefore, our evaluations also suggest that such cell type-specific perturbations to reference signatures may be required for a more accurate deconvolution in higher noise regimes.

The BEDwARS model estimates the true cell type signatures using reference signatures as prior information. This means that $G\times C$ parameters are learnt from the entire bulk data, where $G$ and $C$ are the numbers of genes and cell types respectively. Among other things, this implies that one needs to exercise care when using BEDwARS with many cell types and few bulk samples. While it is difficult to make precise recommendations about these numbers, since they depend on the additional data characteristics, we note that our evaluations have been successful with ~40 bulk profiles and ~10 cell types. Scenarios with more bulk profiles and fewer cell types should be safe for BEDwARS application, and further tests are needed for other scenarios. These considerations further imply that cell type proportion estimation cannot be done for one bulk profile at a time and all bulk samples should be provided at once to BEDwARS, even though the estimated proportions are different for each sample.

Applying BEDwARS to a new dataset, we were able to gain new insight into a rare pediatric inborn error of metabolism linked to DPD deficiency. Using a limited set of scRNAseq data to generate reference signatures, we deconvolved bulk RNAseq data from complex patient-derived neural organoids to identify novel expression changes in specific neural cell types. These findings suggest that multiple changes likely contribute to the pathology of the disorder, including disruptions to ciliated cell function, mitochondrial dysfunction, and alterations to translational machinery at the ER and associated with free ribosomes. While pediatric DPD deficiency has previously been suspected of having a mitochondrial component [32], to our knowledge, this is the first reported evidence for possibly involvement of ciliopathy and impaired translational control in the etiology of the disorder. Further study of these pathways as potential targets for the development of new treatments for ameliorate the symptoms associated with pediatric DPD deficiency is warranted.

Based on our experience as well as on theoretical grounds, we believe BEDwARS may not be able to accurately tease apart the contributions/proportions of highly correlated cell types [4, 38, 39], a common unsolved problem with deconvolution methods. We also believe that BEDwARS performance can be further improved by modifying it to be more robust to outliers, e.g., by changing the optimization objective or through outlier detection and removal. Furthermore, BEDwARS can be improved by accommodating for higher granularity of signature adjustment, i.e., sample specific signatures. We leave these important engineering challenges for future iterations of the tool.

## Conclusions

We implemented a Bayesian approach to the deconvolution of bulk expression profiles, specifically to address the challenge of misalignment between reference cell type signatures and the unknown cell type signatures underlying given bulk profiles. Through extensive benchmarking, we demonstrated that our method outperforms leading in-class methods in the estimation of cell type proportions and is more robust to the extent of misalignment between the reference and true cell type signatures. Furthermore, with a few exceptions our method achieves a better estimation of true cell type signatures than the state-of-the-art method, especially for higher noise levels. Application of BEDwARS to dihydropyridine dehydrogenase deficiency provided new insights into the possible involvement of ciliopathy and impaired translational control in the etiology of the disorder.

## Methods

### Preprocessing of datasets used for benchmarking

#### Pancreas data

“Baron”: scRNA-seq data obtained from Baron et al. [16] were used in generating signatures. Count-level data on cells from all four human subjects (3 healthy and 1 T2D) were utilized. “Segerstolpe”: scRNA-seq datasets from Segerstolpe et al. [15] were used, with count-level data on cells from six healthy individuals forming the “Segerstolpe-H” dataset and those from four T2D individuals forming the “Segerstolpe-T2D” dataset. Cells with “not applicable”, “unclassified”, and “co-expression” tags were removed in this step. “Enge”: Count-level scRNA-seq data on cells from eight healthy individuals, reported in [17], formed the “Enge-H” dataset. Except for the Enge-H dataset, six cell types—alpha, beta, gamma, delta, acinar, and ductal—were analyzed. The Enge-H dataset did not contain gamma cell type therefore only the remaining 5 cell types were considered.

Following the quality control procedure of Cobos et al. [1], for each pancreatic dataset, we removed cells with library size, ribosomal content or mitochondrial content more than three median absolute deviations away from the median. Then, only the genes with nonzero counts in at least 5% of all cells were kept. Finally, RPKM normalization was done using hg19 human genome assembly.

#### Brain data

scRNA-seq data from the middle temporal gyrus in human brain, reported by Darmanis et al. [18], were RPKM-normalized using hg19 human genome assembly, following the preprocessing pipeline of Sutton et al. [10], to form the “Darmanis” dataset. The three most abundant cell types—neurons, astrocytes, and oligodendrocytes—were analyzed. “IP” signatures were obtained from FPKM-normalized RNAseq data on immunopurified cells from human adult brain (temporal lobe cortex) [19]. “MM” signatures were formed from FPMK-normalized RNA-seq data on immunopurified mouse brain tissue, reported by Zhang et al. [20]. “CA” signatures represent RPKM-normalized count-level single-nucleus expression in middle temporal gyrus [21], while “NG” signatures were single-nucleus expression of human prefrontal cortex [22], obtained from Sutton et al. [10] without repeating their preprocessing pipeline.

### Generation of cell type signatures and their variants for benchmarking

#### Signature generation

Cell type signatures were generated by averaging the gene expression profiles of all cells of the same type in a dataset. Following Sutton et al. [10], for the signatures generated from Darmanis, IP, MM, and CA datasets, only genes with more than one RPKM/FPKM expression in at least one cell type were retained. NG signature was taken directly from Sutton et al. [10]. Signatures used in brain gene expression deconvolution were restricted to neurons, astrocytes, and oligodendrocytes, matching a similar restriction imposed on the Darmanis data set (see above).

#### Perturbation of signatures

This procedure is performed separately for each cell type, starting with a reference signature and a true signature of that cell type. (The true signature represents the target dataset to be deconvolved and the reference signature reflects the related dataset used by the deconvolution method.) The procedure, described next, modifies the reference signature by adding random noise to it while maintaining a statistical relationship between the reference and true signatures. It is parameterized by a single parameter $\sigma$. First, the signatures are log-transformed. (Genes with zero expression in any cell type were excluded from the reference and true signatures before the transformation.) Next, genes are partitioned into equal-frequency bins based on their expression values in the true signature. Since a reference signature generally exhibits high positive correlation with the true signature (e.g., see Additional file 1: Figs. S1, S6, S9, and S16, genes in a bin that represents high (or low) expression level in the true signature have a high (resp., low) mean expression in the reference signature as well. This is the statistical relationship that the perturbation procedure maintains, as noted next. In the next step, for each bin, the mean expression of genes in that bin is calculated and each gene’s deviation from the mean is scaled by the same constant; this constant is set so that the resulting expression values (of genes in that bin) have a standard deviation of $\sigma$. This step ensures that the average expression of genes in a bin remains unchanged, so the above-mentioned statistical relationship is maintained; at the same time the variance of reference gene expression in each bin is increased to the pre-set level $\sigma$, thereby adding noise to the signature overall. Some examples of the result of such perturbation are shown in Additional file 1: Figs. S1, S6, S9 and S16.

In our benchmarking, we set $\sigma$ to values in the range $[1, 2.25]$ with increments of $0.25$, to define six noise levels called “NL-1” ($\sigma =1$), “NL-2”, … “NL-6” ($\sigma =2.25$), with higher noise levels resulting in lower correlation coefficients between reference and true signatures. The partitioning of genes was done so that each bin has 300 genes when benchmarking the Darmanis dataset with IP signatures and 100 genes when adding noise to the Baron signatures for deconvolution of Segerstolpe-H, Segerstolpe-T2D, and Enge-H datasets. The exception to this was in the benchmarks where the Baron signatures were used with the Segerstolpe-H and Segerstolpe-T2D target datasets, no perturbation was applied to the signatures of acinar and ductal cell types, as the reference signatures were already relatively poorly correlated with the true signatures for these cell types. A similar exception was made for the ductal cell type when adding noise to the Baron signatures for use with the Enge-H dataset.

The above deterministic procedure for signature perturbation was followed by a second procedure that introduces additional noise to the reference signatures. All genes in the same bin (defined above, representing a small range of values of true signature) were further partitioned into bins of four genes each based on their reference expression values; then the reference expression levels of the four genes in each such bin were shuffled. The entire procedure was repeated 10 times to get 10 variants of the deterministically perturbed reference signature from the first procedure (previous paragraph). Thus, for each noise level, we obtained 11 different randomly generated variants of the reference signature, perturbing it similarity to the true signature in a controlled manner. Note: the IP signature (Zhang et al. [19]) and its noisy variants were restricted to include genes with at least two-fold higher expression in one cell type compared to the others.

### Generation of pseudo-bulk mixtures

#### Pancreas datasets

We followed the pseudo-bulk mixture generation pipeline by Cobos et al. [1] with minor modifications. First, we randomly selected the number of cell types to be present in a mixture, uniformly from the range [2, *K*] where *K* is the total number of cell types. Second, the selected number of cell type identities were randomly sampled without replacement. Next, the “true” proportions associated with the selected cell types were uniformly sampled from $\left[0.05, 1\right]$, followed by scaling to ensure that they sum to one. Finally, 100 cells were sampled so that each cell type was represented with its respective proportion and the expression profiles of the sampled cells were averaged to create a pseudo-bulk profile. By repetitions of this process, 100 mixtures with known cell type proportions and a pseudo-bulk expression profile were generated for each of the data sets Segerstolpe-H, Segerstolpe-T2D, and Enge-H.

#### Brain datasets

One hundred mixtures and corresponding pseudo-bulk profiles were generated by sampling (without replacement) 100 cells at a time from the Darmanis dataset [18] and averaging their expression profiles. The same process was used by Sutton et al. [10] to generate the bulk mixtures for this dataset.

### Induced pluripotent stem cells (iPSCs) and cerebral organoids for the study of DPD-deficiency

iPSCs were reprogrammed from skin fibroblasts that were obtained from skin biopsies. Biopsies were collected following written informed consent/assent from the donor and/or guardian and approved by the Mayo Clinic Institutional Review Board (IRB protocol 14-005685). iPSCs were maintained on 60-mm plates coated with hESC-qualified Matrigel (Corning Life Sciences, Corning, NY) in mTeSR Plus medium (StemCell Technologies, Vancouver, Canada) containing 100 units/mL penicillin and 100 mg/mL streptomycin. Cells were grown at 37 °C in humidified air containing 5% CO_2_. Differentiated cells were removed and medium was exchanged every 1–2 days. Cells were passed using ReLeSR (StemCell Technologies).

Cerebral organoids were generated from iPSCs using the StemCell Technologies STEMdiff Cerebral Organoid kit according to the manufacturer’s instructions. For bulk RNAseq analyses, organoids were harvested on day 46, lysed in TRIzol (Invitrogen, Waltham, MA), and stored at –80 °C until RNA extraction. RNA was extracted using the Zymo Research Direct-zol RNA miniprep kit (Zymo Research, Irvine, CA) according to the manufacturer’s instructions. RNAseq libraries were prepared using TruSeq Stranded mRNA reagents (Illumina, San Diego, CA). For scRNAseq, single cells were isolated using the Neural Tissue Dissociation Kit P (Miltenyi Biotec, Gaithersburg, MD) with gentle trituration. Single cell partitioning and scRNAseq library preparation performed using Single Cell Gene Expression reagents on a Chromium Controller (10 × Genomics, Pleasanton, CA) in the Mayo Clinic Medical Genome Facility Genome Analysis Core. RNAseq and scRNAseq libraries were sequenced using 2 × 150 PE chemistry on a NovaSeq 6000 (Illumina) at the University of Minnesota Genomics Center.

For RNAseq, adapter sequences were removed using the TrimGalore wrapper around Cutadapt [40], and reads were aligned to the human genome (hg19) using two-pass mapping and genes expression quantified as gene counts using STAR [41]. scRNAseq data was processed, mapped to hg19, and quantified using the CellRanger pipeline version 6.1.2 implemented on the 10 × Genomics cloud analysis platform.

### Preprocessing of the scRNA-seq and bulk RNA-seq data for the study of DPD-deficiency

#### Quality control, clustering, and marker detection

Scanpy [42] was used to process the combined scRNA-seq data of the non-affected (DPD4) and affected (DPD1) individuals. In the quality control step, cells with less than 1000 genes expressed and genes that were detected in less than 500 cells were removed. Furthermore, cells with more than 5% mitochondrial gene percentages were removed. To cluster the cells, the top 2000 highly variables genes were selected based on the highest standardized variance approach of Stuart et al. [43] implemented as “seurat_v3” in Scanpy. Principal component analysis (PCA) was performed on these highly variable genes and the top 20 PCs were used to build the neighborhood graph of cells. Leiden graph clustering method with resolution 0.8 was used to detect 17 clusters of cells. Most cells for clusters (0,1,2,3,5,6,9,11,15) were from the affected individual (“affected clusters”) and the rest of clusters mostly contained non-affected cells (“non-affected clusters”). Markers for each cluster were identified using *t*-test and Benjamini–Hochberg method was used for multiple hypothesis testing correction. The markers were then filtered by their adjusted *t*-test *p*-value less than 0.05 and logFC greater than 0.25.

#### Cell type assignment details

A cell type was assigned to a cluster if at least two (out of three) criteria were met. The first criterion is based on the average expression of marker genes of the cell type, following Tanaka et al. [27], that had detectable expression in our dataset. Clusters with high average expression of GAP43, STMN2, and DCX were tagged as neuronal clusters. Among these clusters, the expression of either TBR1 or SLC17A7 is indicative of cortical neurons (CN) whereas expression of EOMES is indicative of neurons (NEU). Based on average expression of neuronal marker genes, clusters 0, 1, 2, 4, 5, 6, 8, and 9 had supporting evidence of being cortical neurons and neurons, respectively. Non-neuronal clusters were identified by the high expression of VIM, HES1, and SOX2. Expression of two other markers, BGN and DCN, was detected for cluster 12, supporting its assignment to the progenitor cells (PGC) cell type. The cellular level expression of neuronal and non-neuronal marker genes is visualized in Additional file 1: Figs. S35 and S30.

The second criterion was the enrichment of certain Gene Ontology (GO) terms in the computationally derived markers of each cluster following the pipeline of Tanaka et al. [27]. Top 200 markers of each cluster (Additional file 6: Table S7) were tested for their enrichment in specific GO terms using the KnowEnG platform [44]. Clusters 3 and 10 were enriched in astrocyte differentiation, cluster 15 in mitosis-related terms, cluster 13 in motile cilium and epithelial cilium movement, and cluster 16 was also enriched in cilium-related terms (see Additional file 1: Table S8 and Additional file 7: Table S9). Following Figure S1.B of Tanaka et al. [27], we interpreted enrichment in astrocyte differentiation (clusters 3,10), mitosis-related terms (cluster 15), and cilium-related terms (clusters 13, 16) as indicators of astrocytes, neuroepithelial cells (NEC), and cilia-bearing cells (CBC), respectively (See Additional file 1: Table S1).

The third criterion used in cell type assignment was based on the overlap of the top 100 computationally derived markers of a cluster (Additional file 6: Table S7) with the corresponding markers from an annotated cluster in Tanaka et al. [27]. The cell type annotation of the annotated cluster of [27] with the largest overlap is used for labeling our clusters. In cases where multiple annotated clusters of [27] were assigned subtypes of the same cell type, the overlap was averaged over all subtypes. Based on this criterion, clusters 0,1,2,4,5, and 8 should be designated as cortical neurons, cluster 9 as neurons, clusters 3 and10 as astrocytes, clusters 13 and 16 as cilia-bearing cells (CBC), clusters 6, 7, 11, and 14 as intermediate (INTER), and cluster 15 should be tagged as neuroepithelial cells (NEC).

The final cell type assignment was based on presence of at least two of the above three types of supporting evidence. Cluster 6 had conflicting evidence in support of cortical neurons (CN) and intermediate cells (INTER) based on the first and third criteria respectively. Cluster 11 was only supported by the third criterion to be assigned to intermediate cells (INTER). Therefore, clusters 6 and 11 were left unassigned and their indices were used as their “cell types”. Clusters 7 and 14 were identified as non-neuronal clusters and were supported by the overlap criterion only. However, we noted that they are well separated in the UMAP plot, and both can be assigned to BRC or INTER. Through a closer examination of Additional file 1: Fig. S31 we decided to tag clusters 7 and 14 with INTER and BRC cell types. The rest of the clusters were supported by two out of three criteria and were thus reliably annotated with cell types.

#### Cell type signature generation

The preprocessed combined scRNA-seq data from non-affected and affected individuals was further filtered for cells with library size, ribosomal content or mitochondrial content more than three median absolute deviations away from median. Also, only the genes with nonzero counts in at least 5% of all cells were kept. The signature was generated from all non-affected annotated clusters as well as three affected clusters. So, the final signature contained cell types CN, AS, CBC, INTER, BRC, and PGC from the non-affected individual as well as NEC, Neuron, and cluster 11 from the affected individuals. The difference between the number of cell types used in the reference signature [9] and the total number of clusters [17] was due to the existence of multiple clusters being assigned to the same cell type and clusters representing the same cell type being present in both non-affected and affected samples.

The count-level expression of all the cells in the clusters annotated with a cell type were summed, then RPKM-normalized (using hg19 assembly) to generate the final cell type signature. Affected cell types/clusters were used in the signature if they were not found in data from the non-affected individual (NEC and Neuron) or if we were not certain about their annotation (cluster 11). Cluster 6 from the affected individual was excluded in the signature generation as it had conflicting cell type assignment evidence to INTER and CN, both of which had representatives via cluster 11 (having weak evidence of being INTER) or non-affected clusters.

#### Preprocessing the bulk RNA-seq data of affected and non-affected groups

Pseudo-bulk mixtures were generated from the scRNA-seq data of the non-affected (DPD4) and affected (DPD1) individuals separately. One hundred pseudo-bulk mixtures were generated per individual by randomly sampling 100 cells without replacement and summing their count-level expression followed by RPKM-normalization. Furthermore, genes with zero expression in more than 20% of the samples were removed. Similarly, bulk RNA-seq profiles of non-affected and affected organoids (from 48 and 72 individuals respectively) were RPKM-normalized and filtered separately for the genes with zero expression in more than 20% of the samples. The bulk RNA-seq of affected and non-affected samples were batch corrected using ComBat [45] to affected and non-affected pseudo-bulk mixtures, respectively. The deconvolution was performed using the batch-corrected bulk RNA-seq data for each group separately.

### Differential gene expression analysis, clustering of genes, and gene set characterization

After deconvolving the batch-corrected bulk RNA-seq samples, for each cell type, differential gene expression (DGE) analysis was performed with log2-transformed expression values for affected vs non-affected group using Limma package [46]. For cell type pairs (CBC-INTER and CN-Neuron) whose inferred proportion sum matched their true proportion sum—derived from the single cell data—DGE analysis was performed for the sum of their deconvolved bulk profiles. After DGE analysis, genes were clustered into 10 groups by k-means algorithm using their discretized expression log fold-change (logFC) for the nine cell types. The discretization was performed as follows: first, genes with absolute expression logFC greater than 5 in any cell type were excluded. Then the logFC of the remaining genes was discretized to values $\pm 2, \pm 0.2,$ and 0 according to the following assignment rule,

$$\mathrm{Discretized}\left({\mathrm{logFC}}_{\mathrm{gc}}\right)= \left\{\begin{array}{lll}-2, & & {\mathrm{logFC}}_{\mathrm{gc}}< -2\\ -0.2,& & {\mathrm{logFC}}_{\mathrm{gc}}\in \left(-2, -0.2\right)\\ 0, & & {\mathrm{logFC}}_{\mathrm{gc}}\in \left(-0.2, 0.2\right) \\ 0.2,& & {\mathrm{logFC}}_{\mathrm{gc}}\in \left(0.2, 2\right)\\ 2,& & {\mathrm{logFC}}_{\mathrm{gc}}> 2\end{array}\right.$$

where g and c are gene and cell type indices, respectively.

Gene set characterization was performed using the David tool [47, 48] for each set of DE genes and cluster of genes. For each annotation cluster, the significant GO-term (FDR < 0.05) with the least FDR was only considered.

### Model

BEDwARS is a Bayesian probabilistic model for cell type proportion deconvolution specifically designed to adjust for deviations between the reference cell type signatures and the true cell type signatures. The deconvolution is formulated as

1 $${X}_{G\times N}={S}_{G\times C}{W}_{C\times N}+{\mathrm{\rm E}}_{G\times N}$$

where $G,C,$ and $N$ represent the number of genes, cell types, and samples with bulk expression profiles, respectively. $X$ is the bulk expression matrix to be deconvolved, with each column being a $G$-dimensional vector and each dimension representing the bulk expression of a gene in a sample. $S$ is the “true” (but unknown) signature matrix, with each column being the $G$-dimensional expression signature of a cell type. $W$ is the (unknown) proportions matrix, with each column being a $C$-dimensional vector and each dimension representing the proportion of a cell type in a sample. $E$ contains the unmodelled bulk gene expression noise which has $N\left(0, \sigma \right)$ distribution for all genes and bulk samples. Prior distributions for $S$ and $W$ are defined as follows:

2 $$\mathrm{log}\left({S}_{gc}\right)\sim \mathrm{log}\left({S}_{gc}^{r}\right)+\mathrm{N}\left(0, {\sigma }_{c}^{2}\mathrm{ log}({S}_{gc}^{r})\right), \forall \left(g, c\right)\in \left\{1, \dots , G\right\}\times \{1, \dots , C\}$$

3 $${W}_{i}\sim \mathrm{Dirichlet}\left(\alpha {W}_{0}\right), \forall i\in \left\{1, \dots , N\right\}$$

where ${S}_{gc}^{r}$ is the reference expression of gene $g$ in cell type $c$. Equation (2) is the key modeling assumption addressing the deviations between the known reference signature and the unknown true signature underlying the bulk profiles $X$. It states that the (log transformed) expression of a gene in a cell type deviates from the corresponding value in the reference signature by an amount that is normally distributed with zero mean and a variance that is gene- as well as cell type-dependent. This variance term, ${\sigma }_{c}^{2}\mathrm{ log}({S}_{gc}^{r})$ in Eq. (2), is proportional to the (log transformed) reference signature value, thus allowing greater deviations for more abundant genes, and the constant of proportionality ${\sigma }_{c}^{2}$ is cell type-dependent, allowing different cell types to exhibit globally more or less deviations. ${W}_{0}$, a $C$-dimensional probability vector is the mean of a Dirichlet distribution and can be set by user based on prior knowledge of cell type proportions. However, as such information is not commonly available, the value of ${W}_{0}$ is set to ${\left[\frac{1}{C}\right]}_{C}$ by default. $\alpha$ controls the variance of the Dirichlet distribution and its high values are associated with low variation. We also defined priors for the parameters of the distributions above,

$$\begin{array}{l}{\sigma }_{c}\sim \mathrm{HalfCauchy}\left({\beta }_{s}\right), \forall c\in \left\{1, \dots , C\right\}\\ \sigma \sim \mathrm{HalfCauchy}\left({\beta }_{b}\right)\\ \alpha \sim \mathrm{Unif}\left({\alpha }_{\mathrm{min}}, {\alpha }_{\mathrm{max}}\right)\end{array}$$

where${\beta }_{s}$, ${\beta }_{b}, {\alpha }_{\mathrm{min}}$, and ${\alpha }_{\mathrm{max}}$ were set to 1, 5, 0, and 30 for all the tests performed. These values can be set by user. For example, ${\beta }_{s}$ can be set to smaller values to reduce the amount of perturbation added to the reference signature. The Half Cauchy prior was used for the standard deviation as suggested by Gelman [49].

Inference of parameters was done by maximizing the posterior probability of all parameters given the bulk expression profiles $X$,

$$\widehat{\theta }={\mathrm{argmax}}_{\uptheta }\mathrm{Pr}\left(\Theta |X\right)={\mathrm{argmax}}_{\uptheta }\mathrm{Pr}\left(X|\Theta \right)\mathrm{Pr}\left(\Theta \right)$$

where $\Theta =\left\{S, W, {\left\{{\sigma }_{c}\right\}}_{c=1}^{C}, \sigma , \alpha \right\}$. Metropolis–Hastings (MH) sampling was used for maximum a posteriori estimation. In implementing MH algorithm, multiple chains were run in parallel to sample from the posterior distribution. In all chains, $S$ was initialized with the reference signature, $\alpha$ and $\sigma$ were initialized by sampling from their corresponding priors and columns of $W$ were initialized with ${W}_{0}$. For ${\left\{{\sigma }_{c}\right\}}_{c=1}^{C}$, equal number of chains were initialized with ${\left[0.01\right]}_{c=1}^{C}$, ${\left[0.1\right]}_{c=1}^{C}$, ${\left[1\right]}_{c=1}^{C}$ and by sampling from the prior. The “best” chain was selected to estimate the parameters. The criterion for selecting the best chain was the mean squared error between $X$ and $\widehat{X}=\widehat{S}\widehat{W}$, restricted to marker genes identified using the reference signature. For all tests reported in this work, the set of genes with at least four-fold higher expression in one cell type compared to the others were chosen as markers. The inference of parameters was done by averaging over the samples drawn by the best chain after its burn-in period. BEDwARS runs multiple chains in parallel on GPU and is implemented in PyTorch. The number of chains used for benchmarking and DPD-deficiency deconvolution experiments were set to 150 and 100, respectively.

#### Memory and run time analysis

All methods evaluated were run in their default setting. B-mode batch correction was used in CIBERSORTx. The run time and memory requirements of BEDwARS, BayesPrism, and CIBERSORTx are reported in Additional file 1: Table S10 for deconvolution of Segerstolpe-T2D pseudo bulk profiles using Baron signature. A complete guide to computing the memory and run time requirements of BEDwARS is provided on its GitHub page.

### Supplementary Information

**Additional file 1.** Supplementary Figures and Tables.

**Additional file 2: Table S2.** Differential gene expression analysis for DPD deficiency. The summary of differential gene expression analysis using Limma package for bulk, pseudo-bulk, and deconvolved cell type or pairs of cell types (CN-NEU, CBC-INTER) expression profiles (“DGE”). Top 200 DE genes per cell type or pairs of cell types are listed in “Top 200 DE genes per cell type” sheet.

**Additional file 3: Table S4.** Summary of David gene set characterization performed on top 200 DE genes identified by DGE analysis for bulk, bootstrapped pseudo-bulk and deconvolved cell type expression profiles for DPD deficiency. In each annotation cluster the first GO with significant FDR (FDR < 0.05), highlighted with yellow, was considered.

**Additional file 4: Table S5.** Cluster of genes identified by Kmeans clustering based on the pattern of genes’ differential expression across the cell types for DPD deficiency. Each column represents the genes belonging to the same cluster.

**Additional file 5: Table S6.** Summary of David gene set characterization for cluster of genes identified by Kmeans algorithm based on the pattern of genes’ differential expression across the cell types for DPD deficiency. The David results for each cluster are included in a sheet named with the cluster name (Cluster X). Clusters zero and four were excluded as they had more than 2000 genes. David results for top 200 DE genes identified by DGE analysis on bulk and bootstrapped pseudo-bulk are included for convenient comparison.

**Additional file 6: Table S7.** Top 200 markers per cluster of non-affected and affected cells for DPD deficiency. Each column contains the top 200 filtered marker genes for a cluster of cells. These markers were used for assigning cell types to the clusters.

**Additional file 7: Table S9.** KnowEng gene set characterization performed on the markers of a subset of cell clusters for DPD deficiency. These results were used to assign cell types to cluster of non-affected and affected cells. The GO terms that were enriched and used for cell type assignment are summarized in Additional file 1: Table S8 for easier lookup.

**Additional file 8.** The peer review history.

## Data Availability

The datasets used in this study were downloaded from the following links: Baron [51]: https://www.ncbi.nlm.nih.gov/geo/query/acc.cgi?acc=GSE84133 Segrestolpe [52]: https://www.ebi.ac.uk/arrayexpress/experiments/E-MTAB-5061/ Enge [53]: https://www.ncbi.nlm.nih.gov/geo/query/acc.cgi?acc=GSE81547 Darmanis [54]: https://github.com/VCCRI/CIDR-comparisons/tree/master/Brain/Data IP [55]: https://www.ncbi.nlm.nih.gov/geo/query/acc.cgi?acc=GSE73721 CA [56]: https://portal.brain-map.org/atlases-and-data/rnaseq/human-mtg-smart-seq NG [57]: Sutton et al. Supplementary Data 5 MM [58]: Data was shared by Steven Sloan contacted by email (https://www.brainrnaseq.org) Newly generated sequence data used for this study are deposited in the NIH Sequence Read Archive (SRA) under BioProject PRJNA986850. The code for BEDwARS is available at https://github.com/sabagh1994/BEDwARS [59] under MIT license. The source code used in this paper is also deposited at Zenodo [60].
