# Supplementary material for: BEDwARS: a robust Bayesian approach to bulk gene expression deconvolution with noisy reference signatures
Source: Genome Biol. 2023 Aug 3;24:178. doi: 10.1186/s13059-023-03007-7 (PMC10399072; doi:10.1186/s13059-023-03007-7)
Supplement: Supplementary file 1 — Additional file 1. Supplementary Figures and Tables. [file 13059_2023_3007_MOESM1_ESM.pdf]

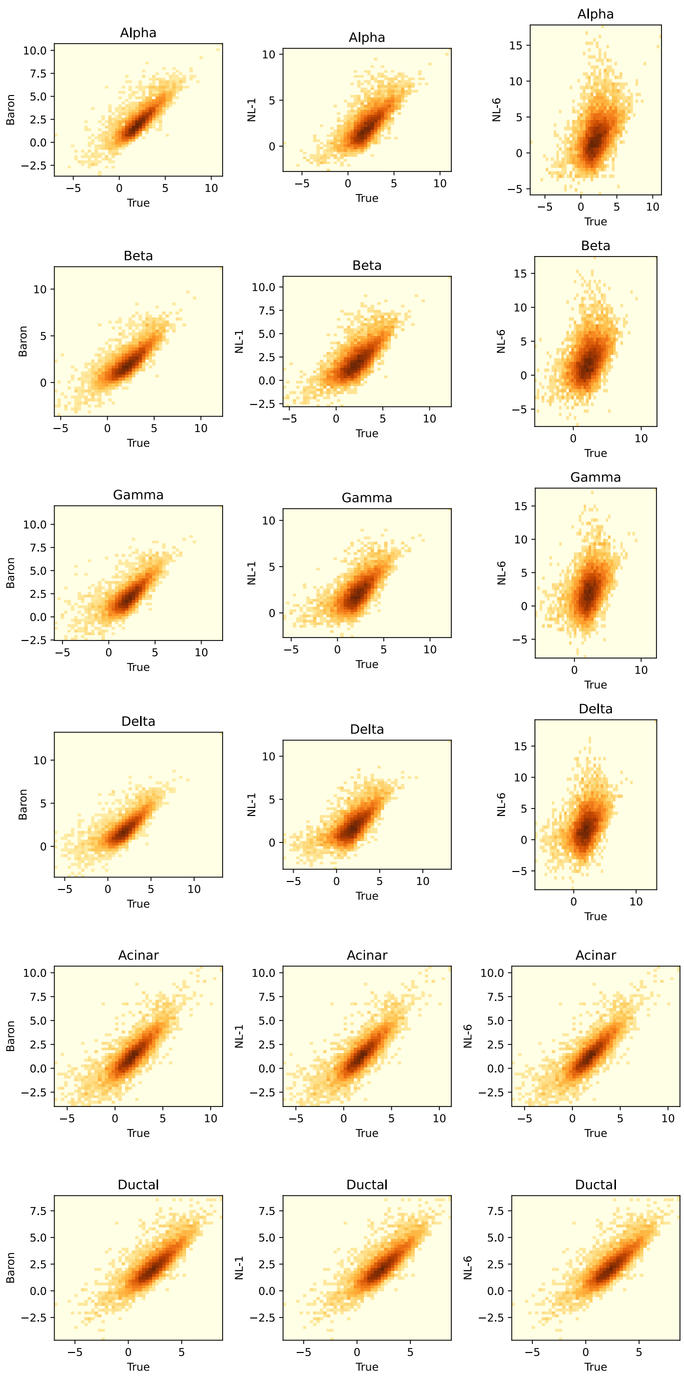

Figure S1. Relationship of Baron and its two noisy variants with the true Segerstolpe-H signature. For each reference signature ("Baron" and its two variants – "NL-1" and "NL-6", representing the lowest and highest levels of added noise respectively), each gene's expression value in that signature is shown on the y-axis and its expression in the true signature (Segerstolpe-H) is shown on the x-axis; both are in log scale. As the noise increases the signatures are less

similar to the true signature. For acinar and ductal cell types, noise is not added to the Baron signature, so the noisy signature (NL-1, NL-6) relationship with the true signature is the same as that of the Baron signature.

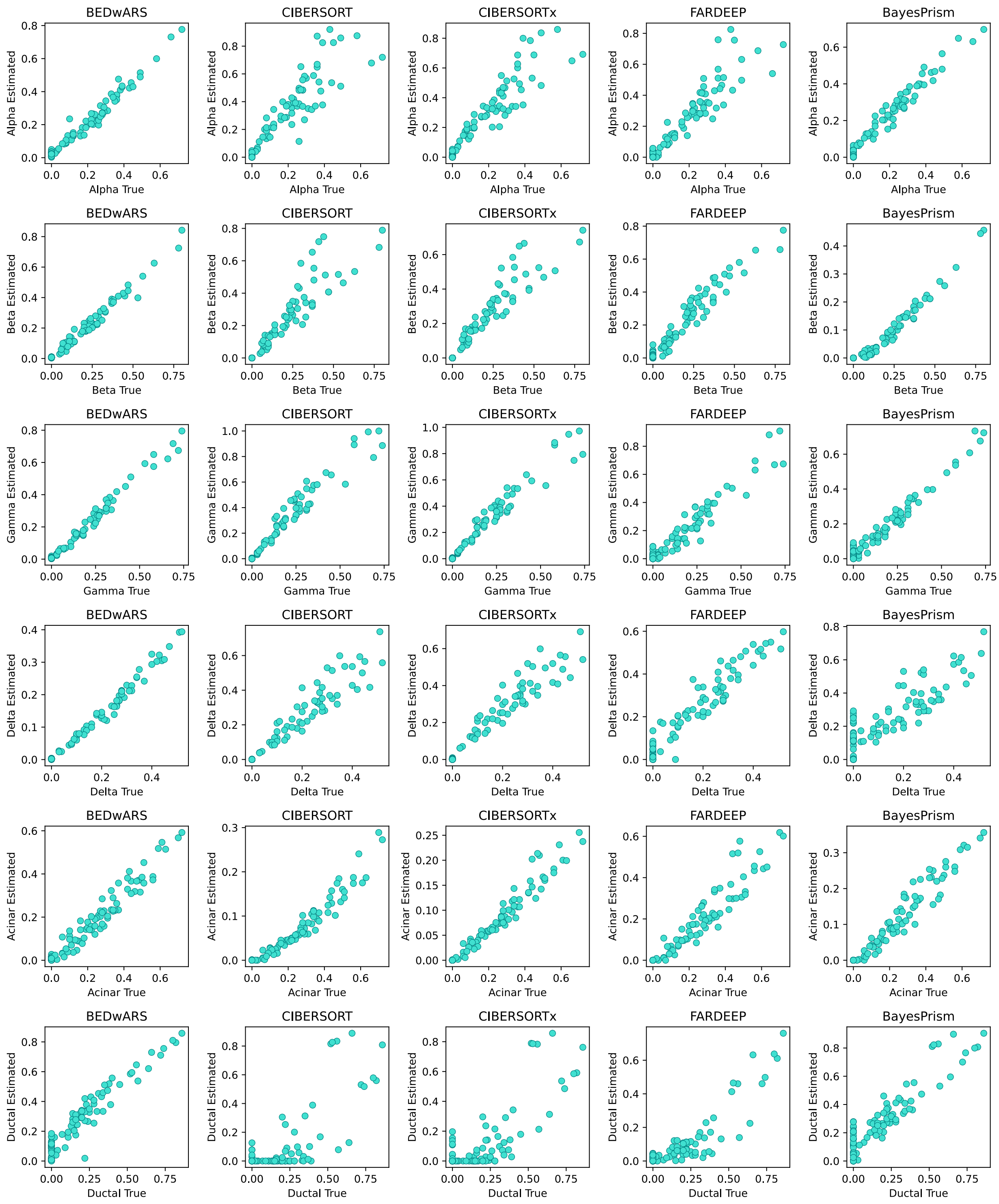

Figure S2. Quality of cell type proportion inference using Baron signature in deconvolving pseudo-bulk samples from Segerstolpe-H. The estimated and true proportions for each of 100 pseudo-bulk samples are compared, for all cell types and all methods (BEDwARS, CIBERSORT, CIBERSORTx, FARDEEP, BayesPrism). BEDwARS estimations match the true values better

than the other methods for all cell types. BayesPrism and CIBERSORT(x) underestimates the acinar proportions by a factor of 2 even though the estimated and true proportions are highly correlated. There is a severe underestimation of ductal proportions by all methods except BEDwARS and BayesPrism.

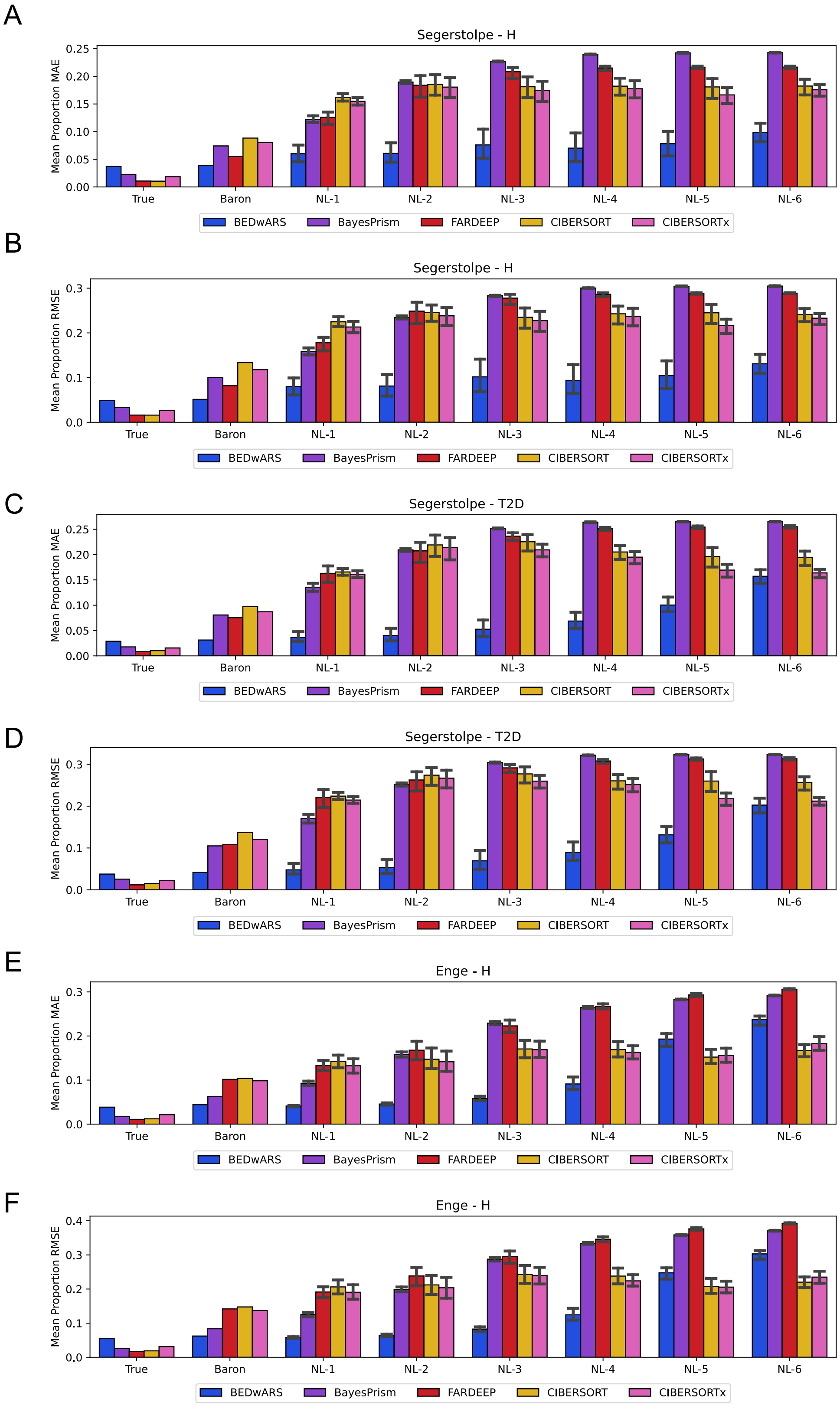
A
Segerstolpe - H
Mean Proportion MAE
0.00
0.05
0.10
0.15
0.20
0.25
True
Baron
NL-1
NL-2
NL-3
NL-4
NL-5
NL-6
BEDwARS
BayesPrism
FARDEEP
CIBERSORT
CIBERSORTx
B
Segerstolpe - H
Mean Proportion RMSE
0.0
0.1
0.2
0.3
True
Baron
NL-1
NL-2
NL-3
NL-4
NL-5
NL-6
BEDwARS
BayesPrism
FARDEEP
CIBERSORT
CIBERSORTx
C
Segerstolpe - T2D
Mean Proportion MAE
0.00
0.05
0.10
0.15
0.20
0.25
True
Baron
NL-1
NL-2
NL-3
NL-4
NL-5
NL-6
BEDwARS
BayesPrism
FARDEEP
CIBERSORT
CIBERSORTx
D
Segerstolpe - T2D
Mean Proportion RMSE
0.0
0.1
0.2
0.3
True
Baron
NL-1
NL-2
NL-3
NL-4
NL-5
NL-6
BEDwARS
BayesPrism
FARDEEP
CIBERSORT
CIBERSORTx
E
Enge - H
Mean Proportion MAE
0.0
0.1
0.2
0.3
True
Baron
NL-1
NL-2
NL-3
NL-4
NL-5
NL-6
BEDwARS
BayesPrism
FARDEEP
CIBERSORT
CIBERSORTx
F
Enge - H
Mean Proportion RMSE
0.0
0.1
0.2
0.3
0.4
True
Baron
NL-1
NL-2
NL-3
NL-4
NL-5
NL-6
BEDwARS
BayesPrism
FARDEEP
CIBERSORT
CIBERSORTx

Figure S3. Performance evaluation, by RMSE and MAE criteria, of methods for estimation of cell type proportions by deconvolving pancreatic pseudo-bulk profiles. Shown are MAE (mean absolute error) (**A, C, E**) and RMSE (root mean squared error) (**B, D, F**) between estimated and true proportions, averaged over the cell types, for all five methods – BEDwARS, BayesPrism, FARDEEP, CIBERSORT, CIBERSORTx– tested by us. RMSE and MAE are computed between the inferred and true cell type proportions of 100 pseudo-bulk samples derived from Segerstolpe-H (**A, B**), Segerstolpe-T2D (**C, D**), and Enge-H (**E, F**) data sets. Evaluations are shown with the Baron reference signature as well as its noisy variants (NL-1, NL-2, … NL-6). BEDwARS has the least RMSE and MAE in Baron group and all noise levels except for the largest two noise levels in (**E, F**). Evaluations are also shown for the hypothetical case where the true underlying signature was available during deconvolution (“True” category in each panel), though this is not a common situation.

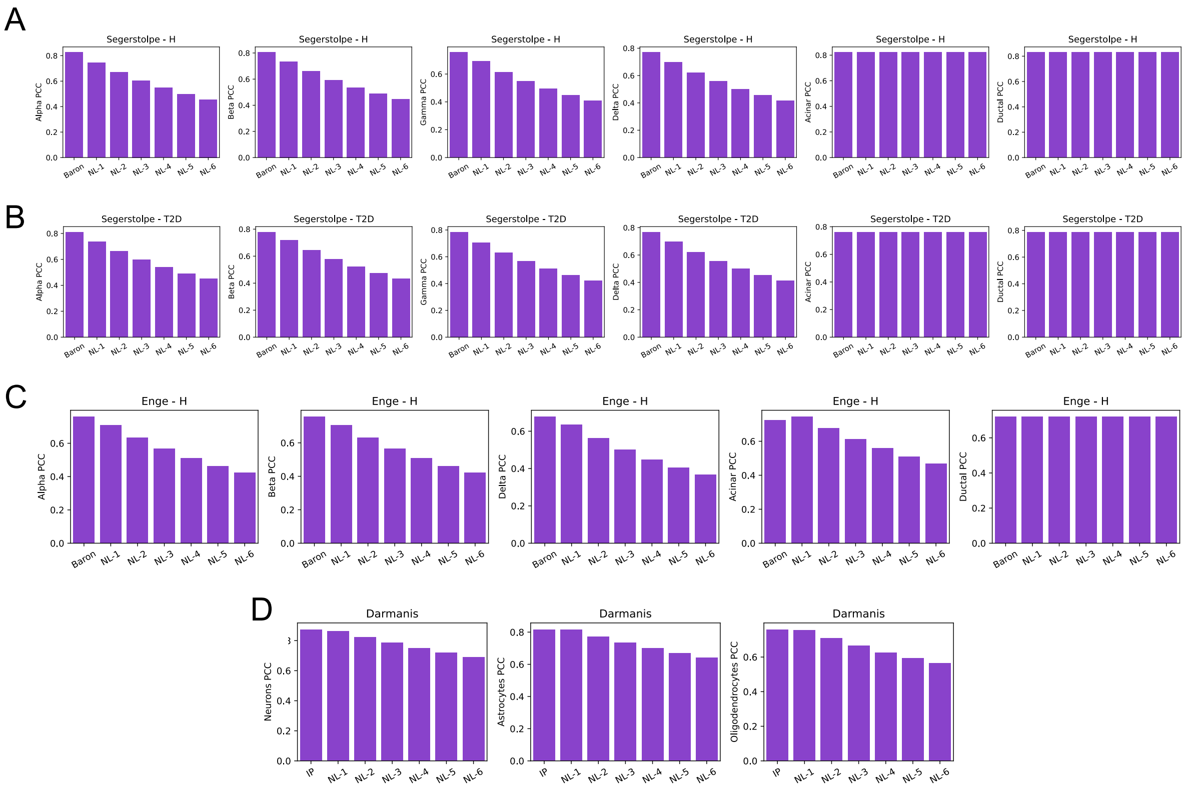

Figure S4. Correlation between reference and true signature at different noise levels, for three pancreatin data sets (A,B,C) and one brain data set (D). Each panel corresponds to a cell type, showing median Pearson correlation coefficient (PCC) between log-transformed reference and true signatures of that cell type, at varying noise levels. In each panel, the first category ("Baron" for A-C and "IP" for D) represents the reference signature without added noise and the remaining categories ("NL-X") represent noisy versions of this signature, with higher values of "X" indicating greater added noise. As the noise level increases, the noisy reference and true signatures become more dissimilar. To avoid large deviations, the signatures of cell types acinar and ductal are not perturbed when creating noisy reference signatures for Segerstolpe-H (A**)** and Segerstolpe-T2D (B) data sets. For the same reason, ductal signature is not perturbed in the Enge-H (C) data set.

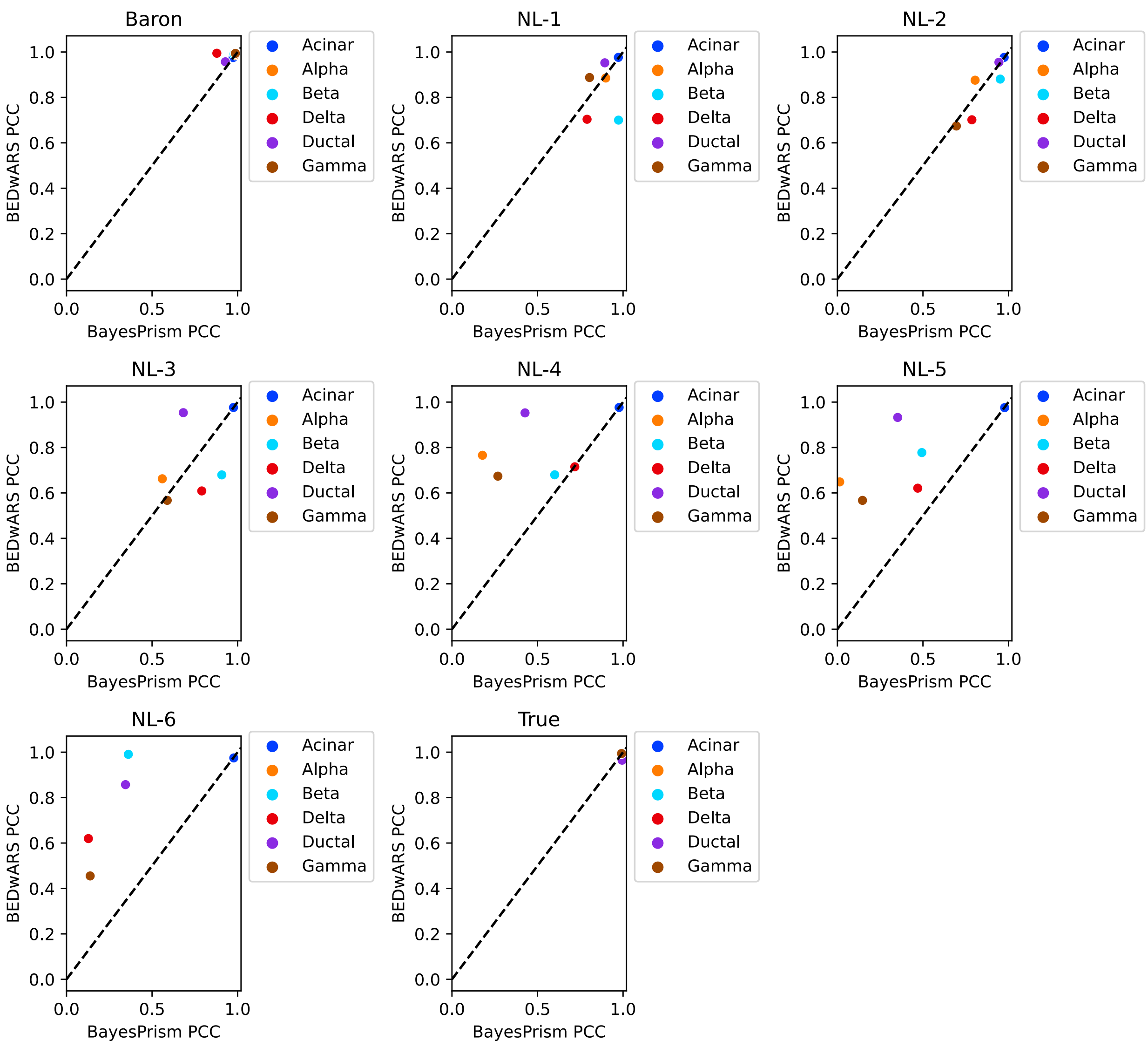

Figure S5. Cell type level comparison of BEDwARS and BayesPrism for the task of proportion estimation by deconvolution of Segerstolpe-H pseudo-bulk samples. Different panels correspond to different reference signatures used during deconvolution -- the Baron signature and its noisy variants NL-1, NL-2, … NL-6, as well as the true signature. Performance is measured by the Pearson Correlation Coefficient (PCC) between estimated and true proportions of a cell type, across the 100 pseudo-bulk samples. For evaluations with noisy signatures, the average over 11 noisy signatures at the same noise level is shown. The performance gap between BEDwARS and BayesPrism is large at higher noise levels for most of the cell types. The performance gap is small for lower noise levels NL-1,2,3 and Baron signature. The performance gap is absent when the true signature is used.

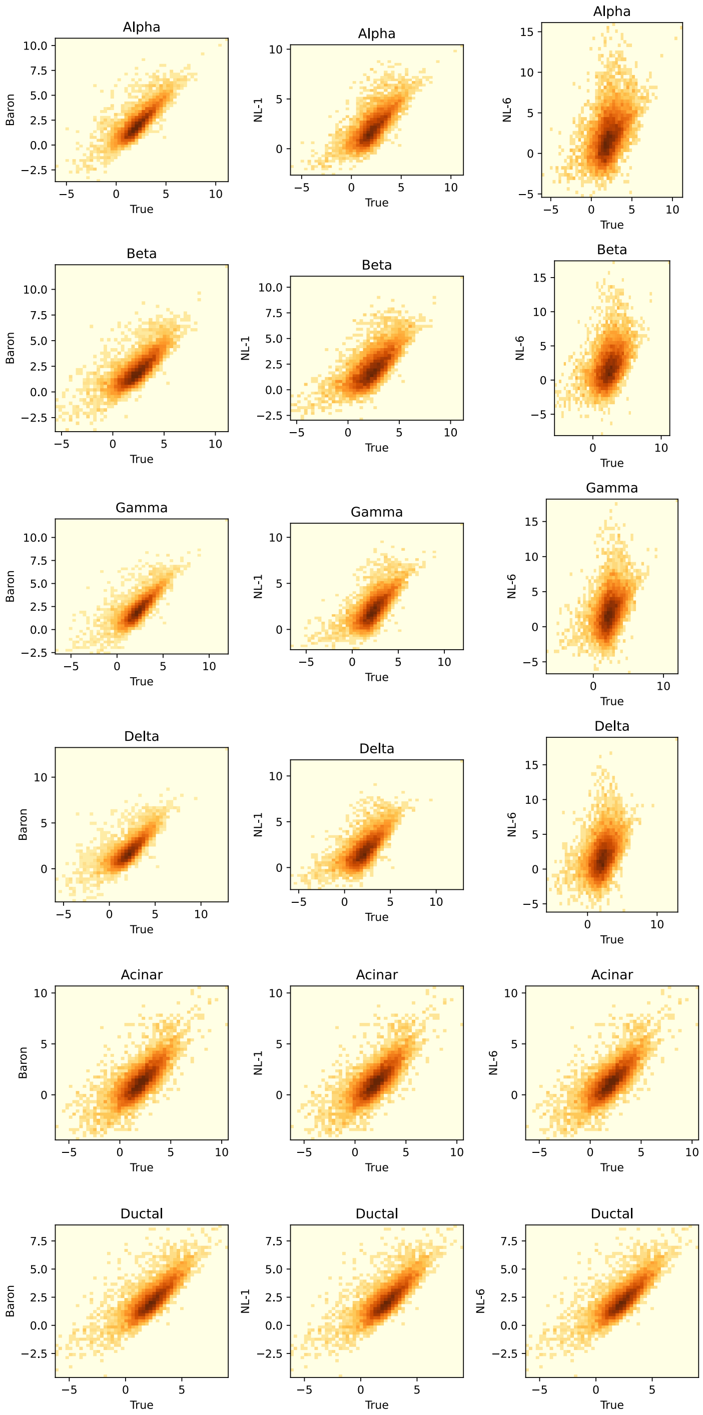

Figure S6. Relationship of Baron and its two noisy variants with the true Segerstolpe-T2D signature. For each reference signature ("Baron" and its two variants – "NL-1" and "NL-6", representing the lowest and highest levels of added noise respectively), each gene's expression value in that signature is shown on the y-axis and its expression in the true signature (Segerstolpe-

T2D) is shown on the x-axis; both are in log scale. As the noise increases the signatures are less similar to the true signature. For acinar and ductal cell types, noise is not added to the Baron signature, so the noisy signature (NL-1, NL-6) relationship with the true signature is the same as that of the Baron signature.

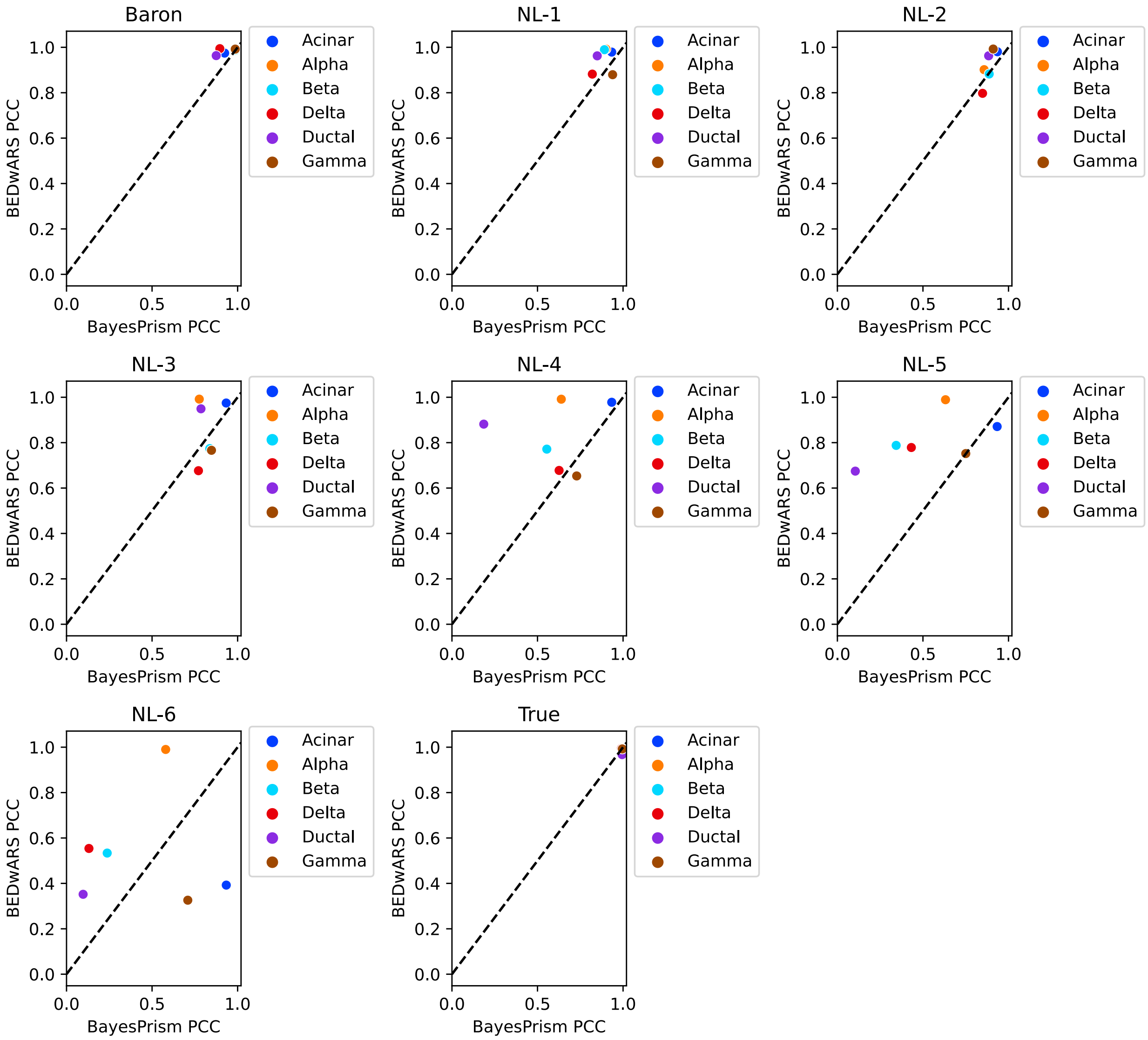

Figure S7. Cell type level comparison of BEDwARS and BayesPrism for the task of proportion estimation by deconvolution of Segerstolpe-T2D pseudo-bulk samples. Different panels correspond to different reference signatures used during deconvolution -- the Baron signature and its noisy variants NL-1, NL-2, … NL-6, as well as the true signature. Performance is measured by the Pearson Correlation Coefficient (PCC) between estimated and true proportions of a cell type, across the 100 pseudo-bulk samples. For evaluations with noisy signatures, the average over 11 noisy signatures at the same noise level is shown. The performance gap between BEDwARS and CIBERSORTx is large at higher noise levels for most of the cell types. The performance gap is small for lower noise levels NL-1,2,3 and Baron signature. The performance gap is absent when the true signature is used.

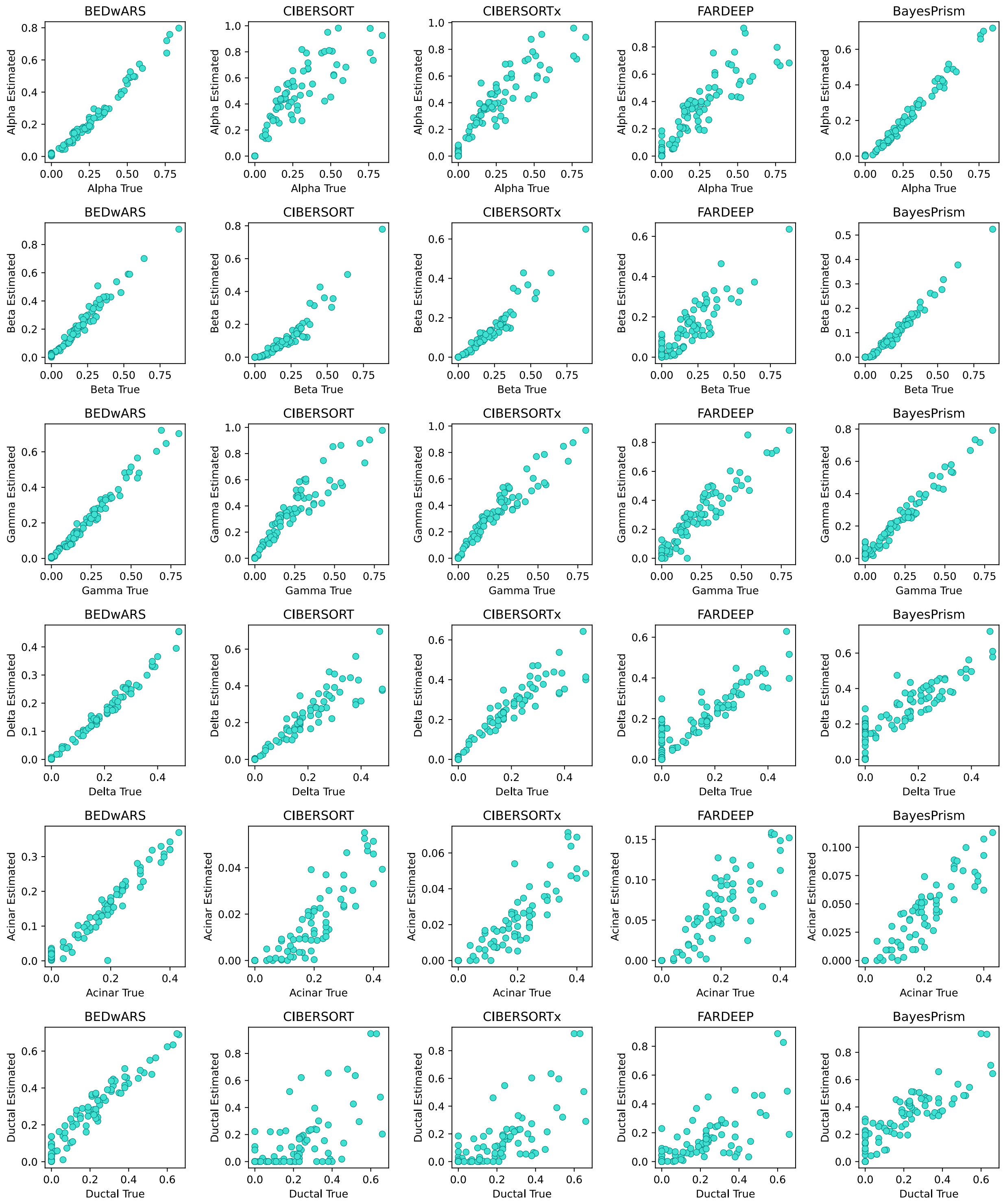

Figure S8. Quality of cell type proportion inference using Baron signature in deconvolving pseudo-bulk samples from Segerstolpe-T2D. The estimated and true proportions for each of 100 pseudo-bulk samples are compared, for all cell types and all methods (BEDwARS,

CIBERSORT, CIBERSORTx, FARDEEP, BayesPrism). BEDwARS estimations match the true values better than the other methods for all cell types. CIBERSORT(x) overestimates proportions of alpha, gamma, and delta cell types and underestimate acinar and ductal proportions. BayesPrism overestimates delta and ductal cell types and underestimates alpha, beta and acinar proportions. FARDEEP also frequently overestimates the small proportions and severely underestimates proportions of acinar and ductal cell types.

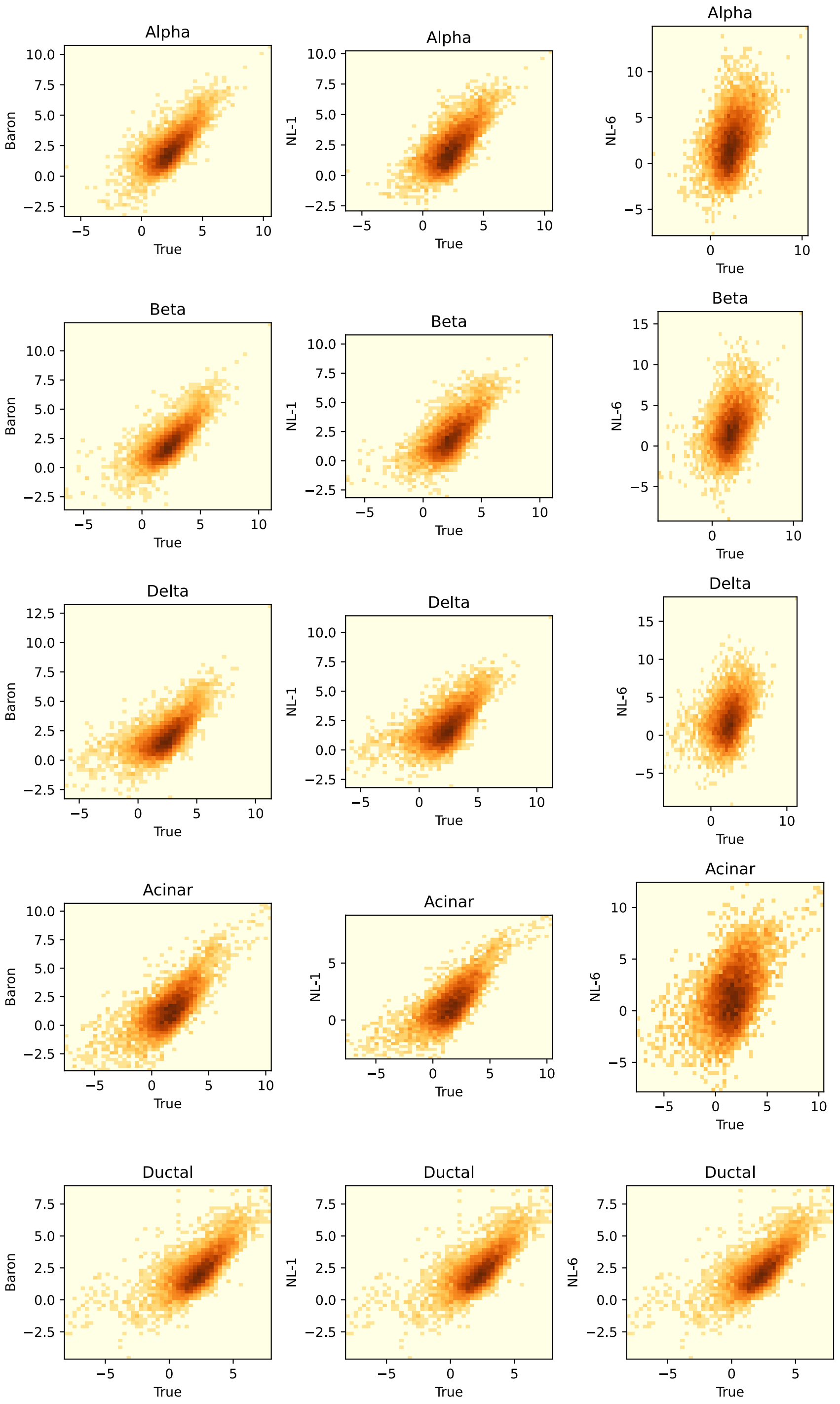
Alpha
Baron
True
Alpha
NL-1
True
Alpha
NL-6
True
Beta
Baron
True
Beta
NL-1
True
Beta
NL-6
True
Delta
Baron
True
Delta
NL-1
True
Delta
NL-6
True
Acinar
Baron
True
Acinar
NL-1
True
Acinar
NL-6
True
Ductal
Baron
True
Ductal
NL-1
True
Ductal
NL-6
True

Figure S9. Relationship of Baron and its two noisy variants with the true Enge-H signature. For each reference signature ("Baron" and its two variants – "NL-1" and "NL-6", representing the lowest and highest levels of added noise respectively), each gene's expression value in that signature is shown on the y-axis and its expression in the true signature (Enge-H) is shown on the x-axis; both are in log scale. As the noise increases the signatures are less similar to the true signature. For ductal cell type, noise is not added to the Baron signature, so the noisy signature (NL-1, NL-6) relationship with the true signature is the same as that of the Baron signature.

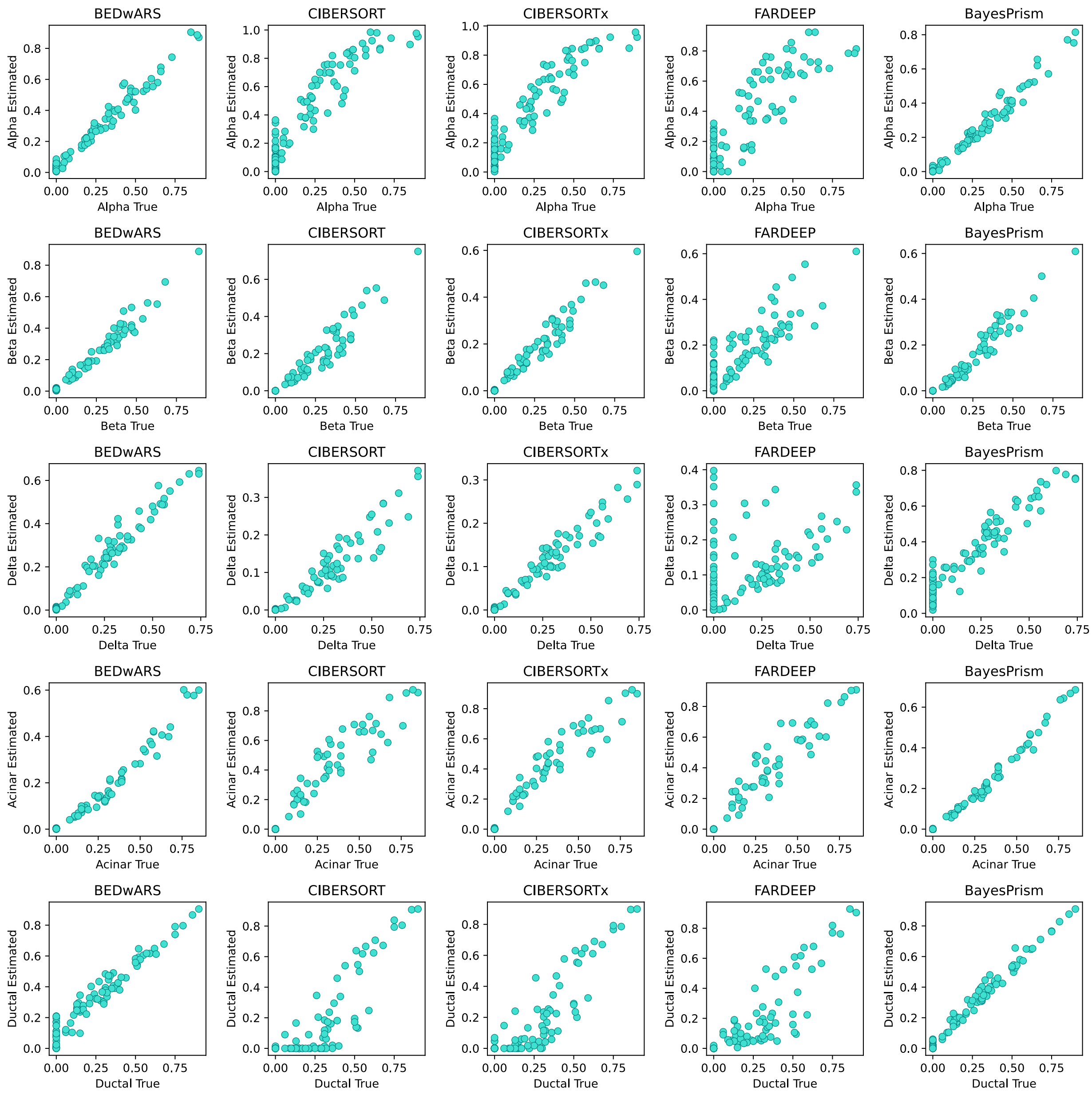

Figure S10. Quality of cell type proportion inference using Baron signature in deconvolving pseudo-bulk samples from Enge-H. The estimated and true proportions for each of 100 pseudo-bulk samples are compared, for all cell types and all methods (BEDwARS, CIBERSORT, CIBERSORTx, FARDEEP, BayesPrism). BEDwARS estimations match the true values better than the other methods for all cell types except delta for which BayesPrism has better estimations. All methods except BEDwARS and BayesPrism show a severe overestimation of zero-valued alpha proportions. CIBERSORT(x) underestimates delta proportions by 2 folds and BayesPrism has a severe overestimation of zero-valued delta proportions. FARDEEP

significantly overestimates the zero-valued delta proportions and underestimates the highest delta proportions by 2 folds.

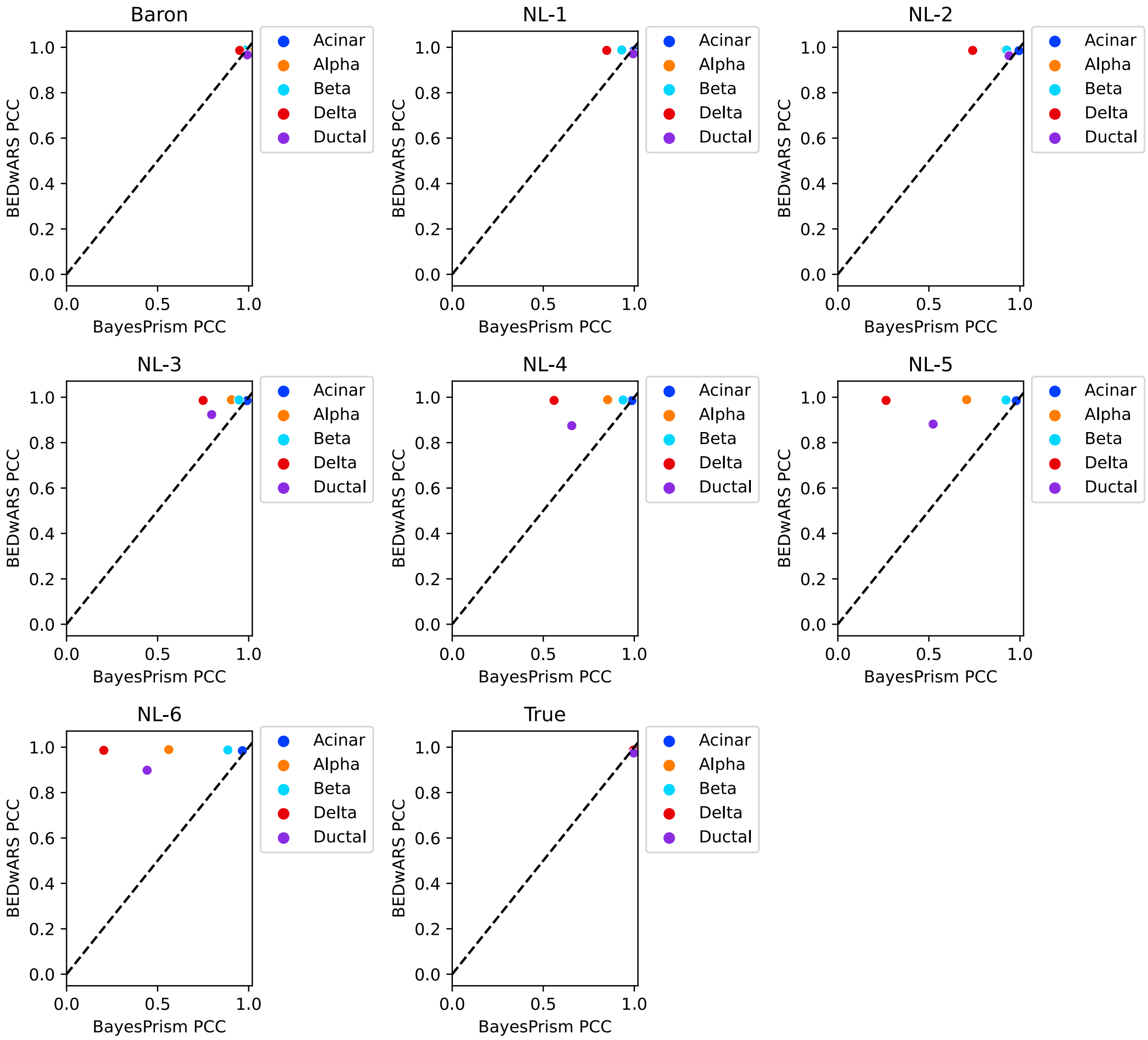

Figure S11. Cell type level comparison of BEDwARS and BayesPrism for the task of proportion estimation by deconvolution of Enge-H pseudo-bulk samples. Different panels correspond to different reference signatures used during deconvolution -- the Baron signature and its noisy variants NL-1, NL-2, … NL-6, as well as the true signature. Performance is measured by the Pearson Correlation Coefficient (PCC) between estimated and true proportions of a cell type, across the 100 pseudo-bulk samples. For evaluations with noisy signatures, the average over 11 noisy signatures at the same noise level is shown. The performance gap between BEDwARS and BayesPrism is large at higher noise levels NL-4,5,6 for most of the cell types. The performance gap is small in the case of the Baron signature, and absent when the true signature is used.

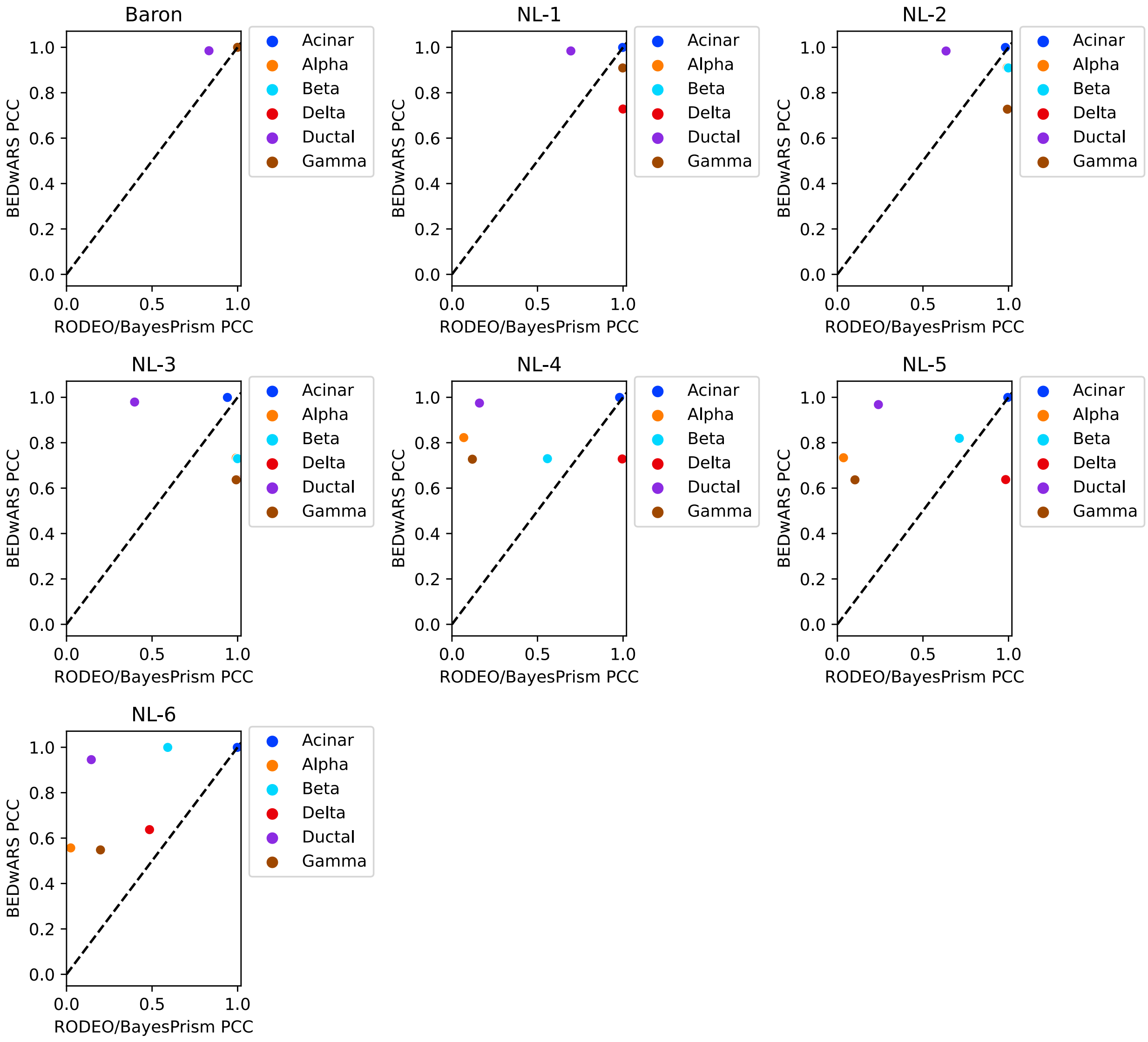

Figure S12. Cell type level comparison of BEDwARS and RODEO/Bayesprism for the task of signature estimation by deconvolution of Segerstolpe-H pseudo-bulk samples. Different panels correspond to different reference signatures used during deconvolution -- the Baron signature and its noisy variants NL-1, NL-2, … NL-6. Performance is measured by the Pearson Correlation Coefficient (PCC) between estimated and true signatures of a cell type. For evaluations with noisy signatures, the average over 11 noisy signatures at the same noise level is shown. BEDwARS performance is notably better than RODEO provided with BayesPrism estimates of proportions at higher noise levels NL-4,5,6 except for the delta cell type at noise levels NL-4 and NL-5. BEDwARS is better for ductal cell type in the case of the Baron signature.

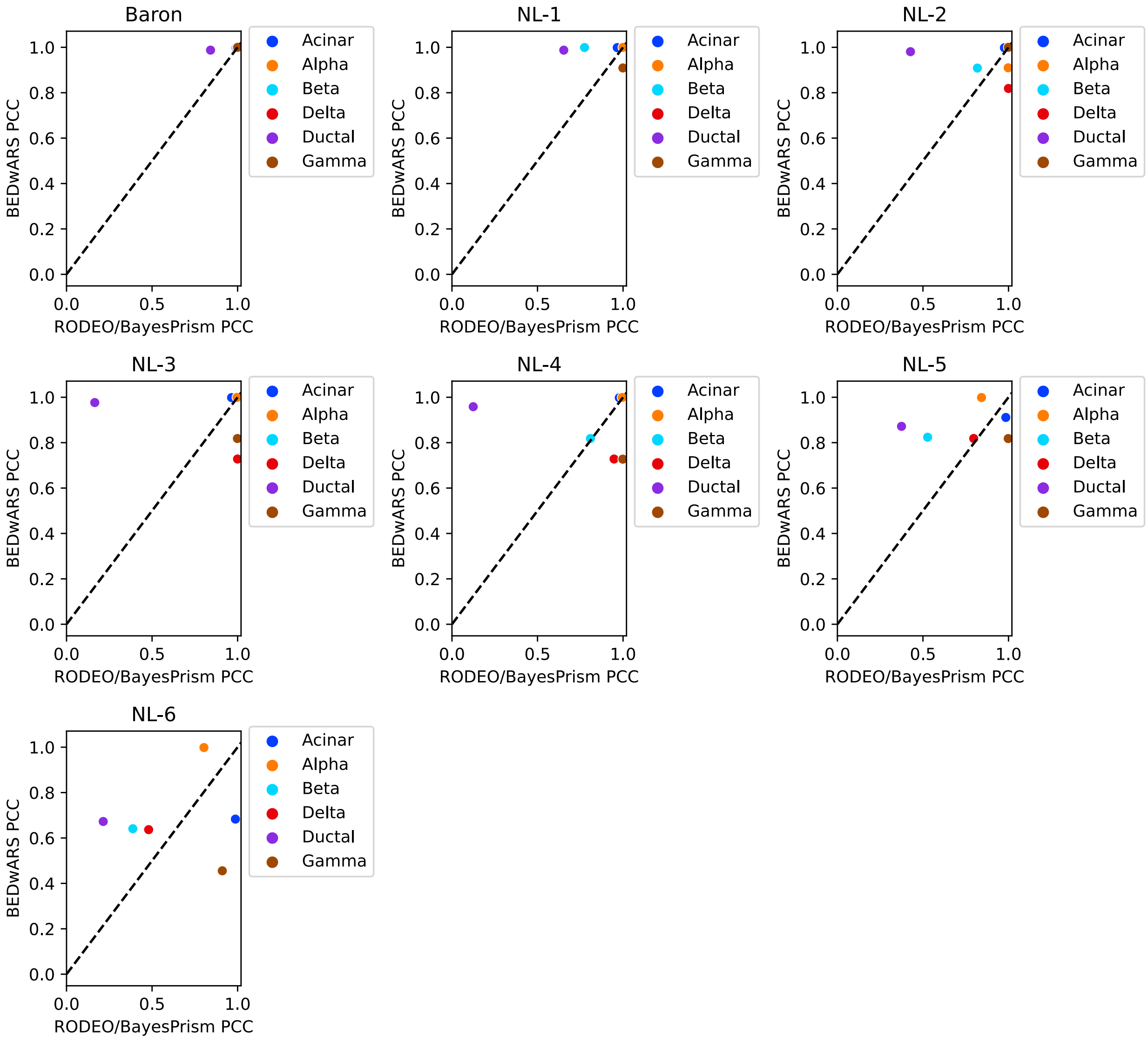

Figure S13. Cell type level comparison of BEDwARS and RODEO/BayesPrism for the task of signature estimation by deconvolution of Segerstolpe-T2D pseudo-bulk samples. Different panels correspond to different reference signatures used during deconvolution -- the Baron signature and its noisy variants NL-1, NL-2, … NL-6. Performance is measured by the Pearson Correlation Coefficient (PCC) between estimated and true signatures of a cell type. For evaluations with noisy signatures, the average over 11 noisy signatures at the same noise level is shown. BEDwARS performance is notably better than RODEO provided with BayesPrism estimates of proportions at higher noise levels and cell types except for gamma, delta (NL-4) and acinar (NL-6). BEDwARS is better for ductal cell type in the case of the Baron signature.

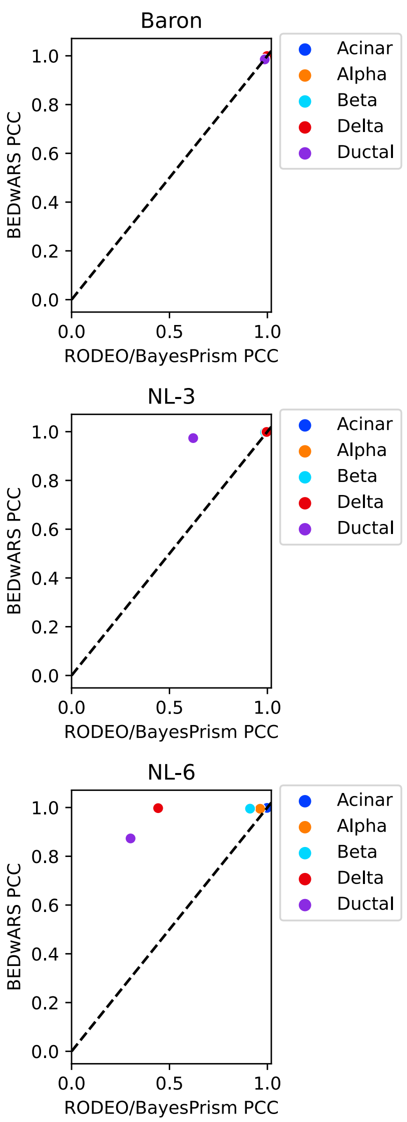

Figure S14. Cell type level comparison of BEDwARS and RODEO/BayesPrism for the task of signature estimation by deconvolution of Enge-H pseudo-bulk samples. Different panels correspond to different reference signatures used during deconvolution -- the Baron signature and its noisy variants NL-1, NL-2, … NL-6. Performance is measured by the Pearson Correlation Coefficient (PCC) between estimated and true signatures of a cell type. For evaluations with noisy signatures, the average over 11 noisy signatures at the same noise level is shown. BEDwARS is similar to or better than RODEO provided with BayesPrism-estimated proportions at all noise levels. When the Baron signature is used (without added noise) both methods perform equally well.

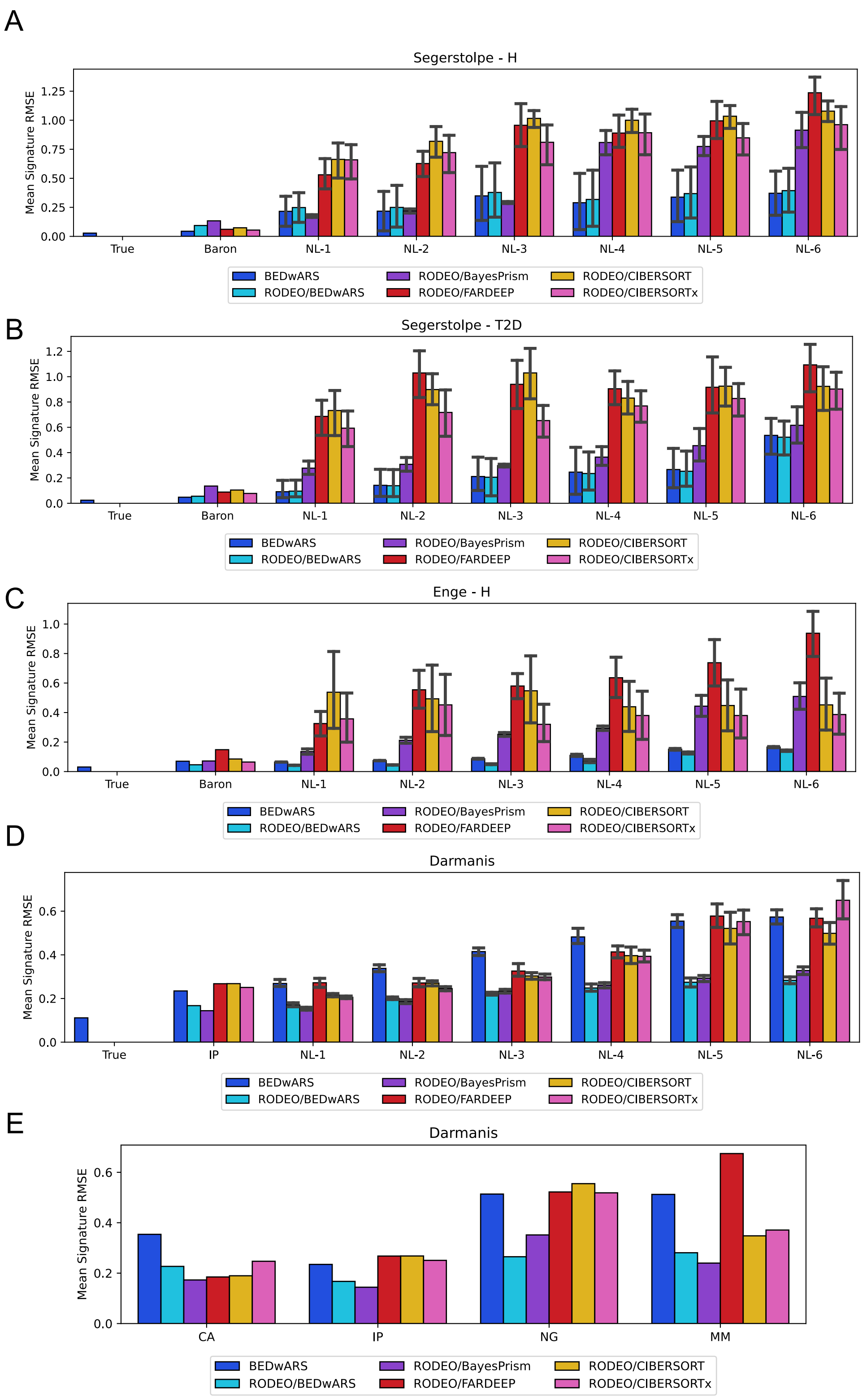

Figure S15. Performance evaluation, by RMSE criterion, of methods for estimating cell type signatures by deconvolving bulk transcriptomic profiles with a given reference signature.

Shown is the RMSE between estimated and true gene expression values, averaged over the cell types, for all five methods tested by us. Four of the methods utilize RODEO after estimating cell type proportions using a deconvolution method – BEDwARS, BayesPrism, FARDEEP, CIBERSORT or CIBERSORTx – these are named following the template “RODEO/M” where M is the method used for cell type proportion estimation. The sixth method evaluated is BEDwARS, since BEDwARS simultaneously estimates signatures as well as proportions. RMSE is computed between the inferred and true cell type signatures for the task of deconvolving 100 pseudo-bulk samples generated from Segerstolpe-H (**A**), Segerstolpe-T2D (**B**), Enge (**C**), and Darmanis (**D, E**) datasets. In **A, B** BEDwARS (RODEO/BEDwARS) has the least RMSE for Baron signature without added noise and higher noise levels NL-4,5,6. For lower noise levels, BEDwARS (RODEO/BEDwARS) is similar to RODEO/BayesPrism in (**B)**. BEDwARS (RODEO/BEDwARS) has the least RMSE at all noise levels in **C.** In the deconvolution of brain transcriptomic profiles, RODEO/BEDwARS and RODEO/BayesPrism have similar average RMSE which is less than other methods using IP and its noisy versions (**D**). RODEO/BEDwARS has the least RMSE for NG signature (**E**).

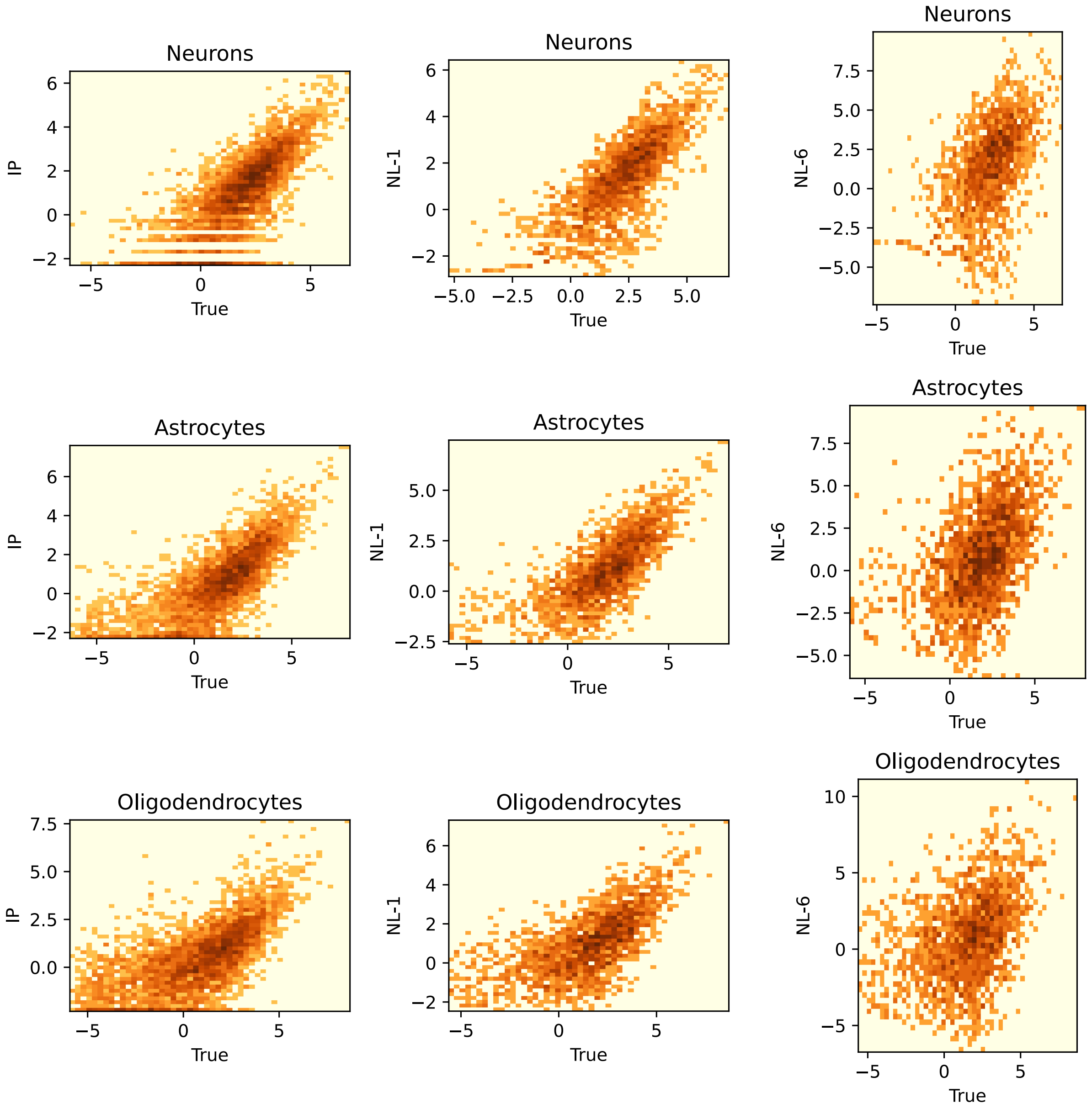

Figure S16. Relationship of IP and its two noisy variants with the true Darmanis signature. For each reference signature ("IP" and its two variants – "NL-1" and "NL-6", representing the lowest and highest levels of added noise respectively), each gene's expression value in that signature is shown on the y-axis and its expression in the true signature (Darmanis) is shown on the x-axis; both are in log scale. As the noise increases the signatures are less similar to the true signature. IP signature is highly diverged from the Darmanis signature.

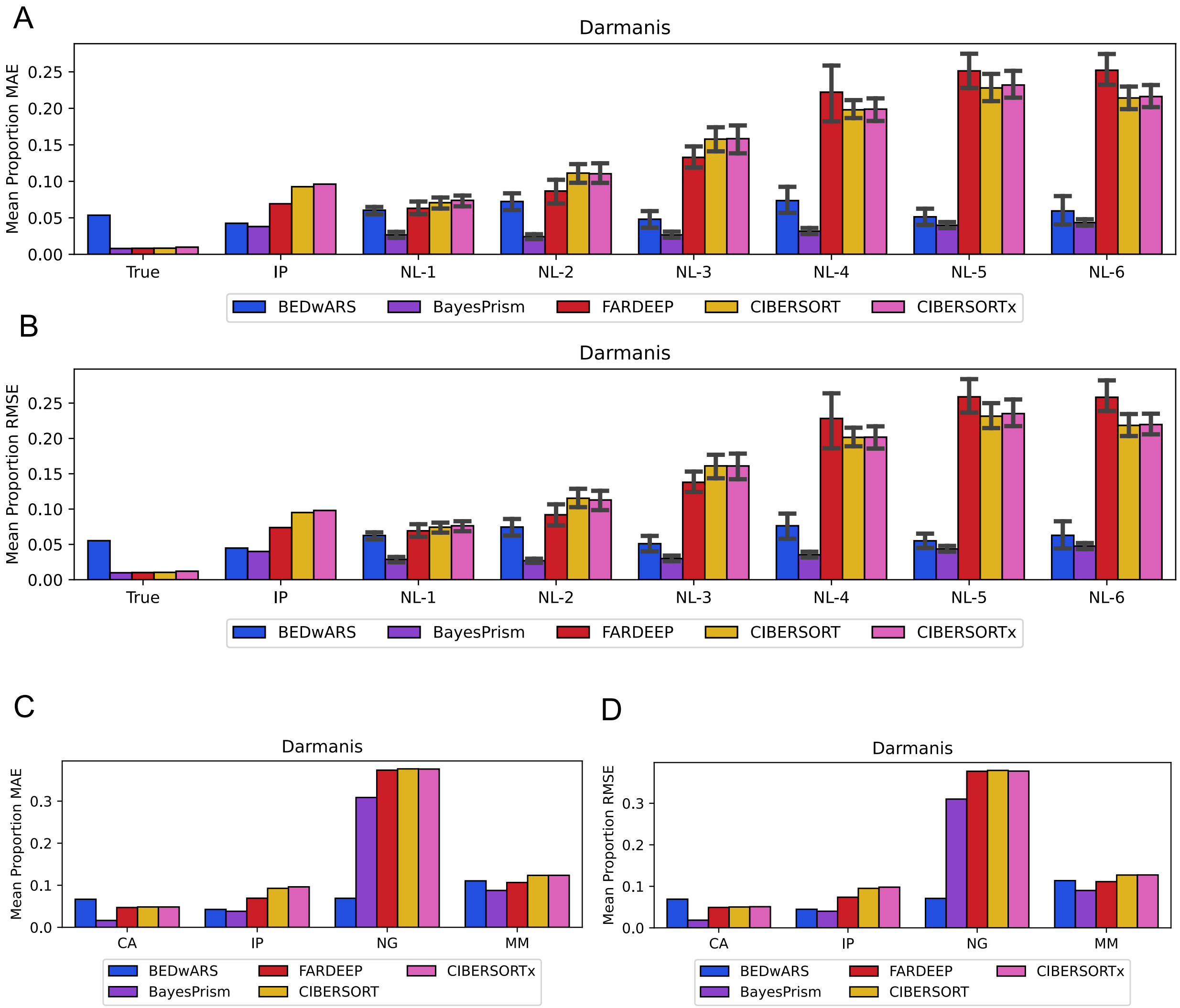

Figure S17. Performance evaluation, by RMSE and MAE criteria, of methods for estimation of cell type proportions by deconvolving pancreatic and brain transcriptomic/pseudo-bulk profiles. Shown are MAE (mean absolute error) (**A, C**) and RMSE (root mean squared error) (**B, D**) between estimated and true proportions, averaged over the cell types, for all five methods – BEDwARS, BayesPrism, FARDEEP, CIBERSORT, CIBERSORTx – tested by us. RMSE and MAE are computed between the inferred and true cell type proportions of 100 pseudo-bulk samples derived from Darmanis dataset. Evaluations are shown with the IP reference signature as well as its noisy variants (NL-1, NL-2, … NL-6). BEDwARS and BayesPrism have similar RMSE for IP and noise levels NL-3, NL-5 and NL-6. Evaluations are also shown for the hypothetical case where the true underlying signature was available during deconvolution ("True" category in each panel), though this is not a common situation (**A, B**). Similarly, BEDwARS has

the best performance by a large margin in both criteria using NG signature as the reference signature and it is close to other methods using CA and MM (**C, D**).

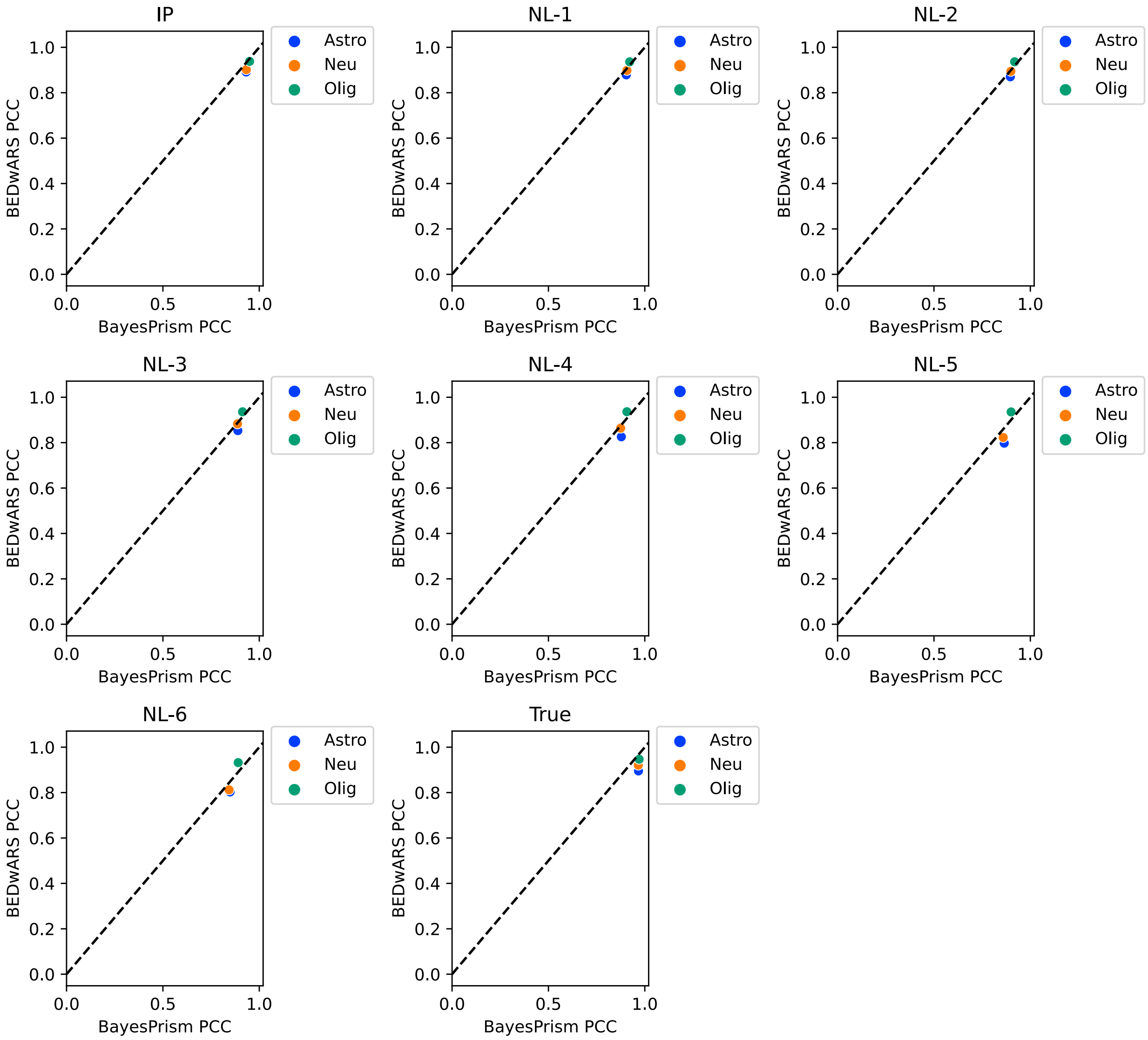

Figure S18. Cell type level comparison of BEDwARS and BayesPrism for the task of proportion estimation by deconvolution of Darmanis pseudo-bulk samples. Different panels correspond to different reference signatures used during deconvolution -- the IP signature and its noisy variants NL-1, NL-2, … NL-6, as well as the true signature. Performance is measured by the Pearson Correlation Coefficient (PCC) between estimated and true proportions of a cell type, across the 100 pseudo-bulk samples. For evaluations with noisy signatures, the average over 11 noisy signatures at the same noise level is shown. BEDwARS and BayesPrism are similar in all the cases.

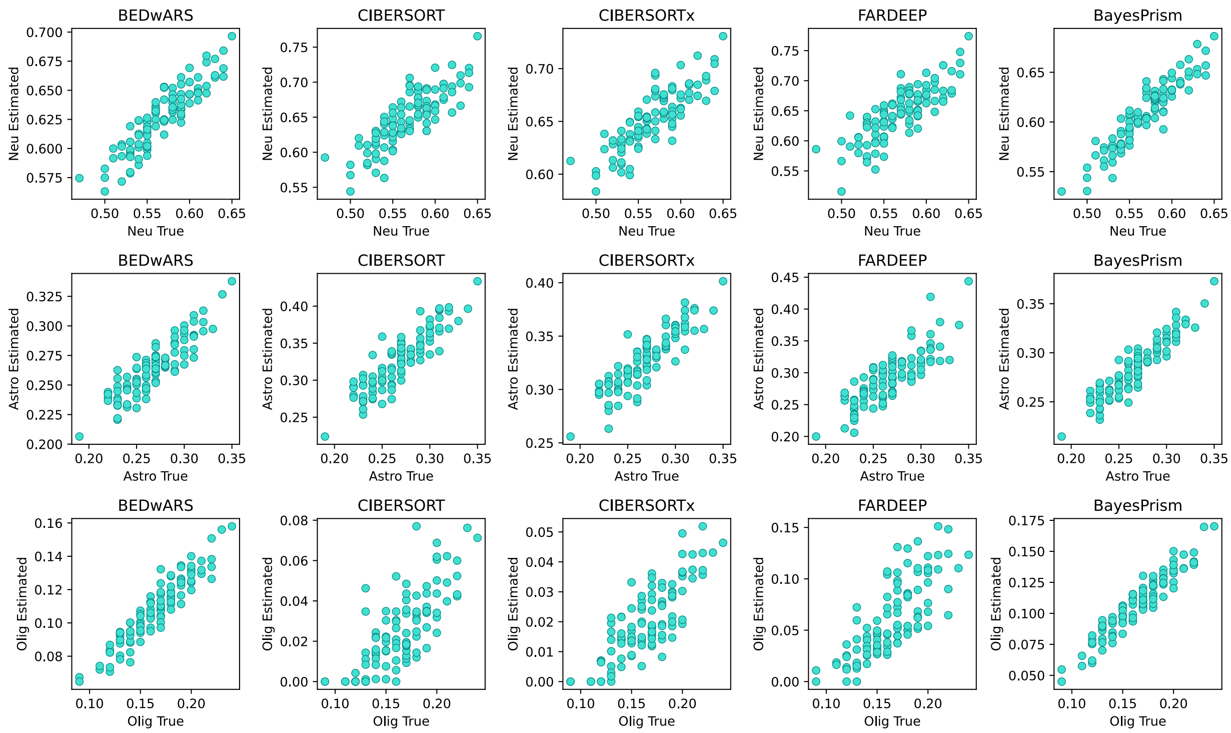

Figure S19. Quality of cell type proportion inference using IP signature in deconvolving pseudo-bulk samples from Darmanis. The estimated and true proportions for each of 100 pseudo-bulk samples are compared, for all cell types and all methods (BEDwARS, CIBERSORT, CIBERSORTx, FARDEEP). Overall, BEDwARS-estimated proportions have higher correlation with true proportions than other methods. The performance gap between BEDwARS and other methods except BayesPrism is larger for oligodendrocytes, for which CIBERSORT(x) underestimates the highest proportions by nearly 10-fold. Neurons, astrocytes and oligodendrocytes are abbreviated as Neu, Astro, and Olig, respectively.

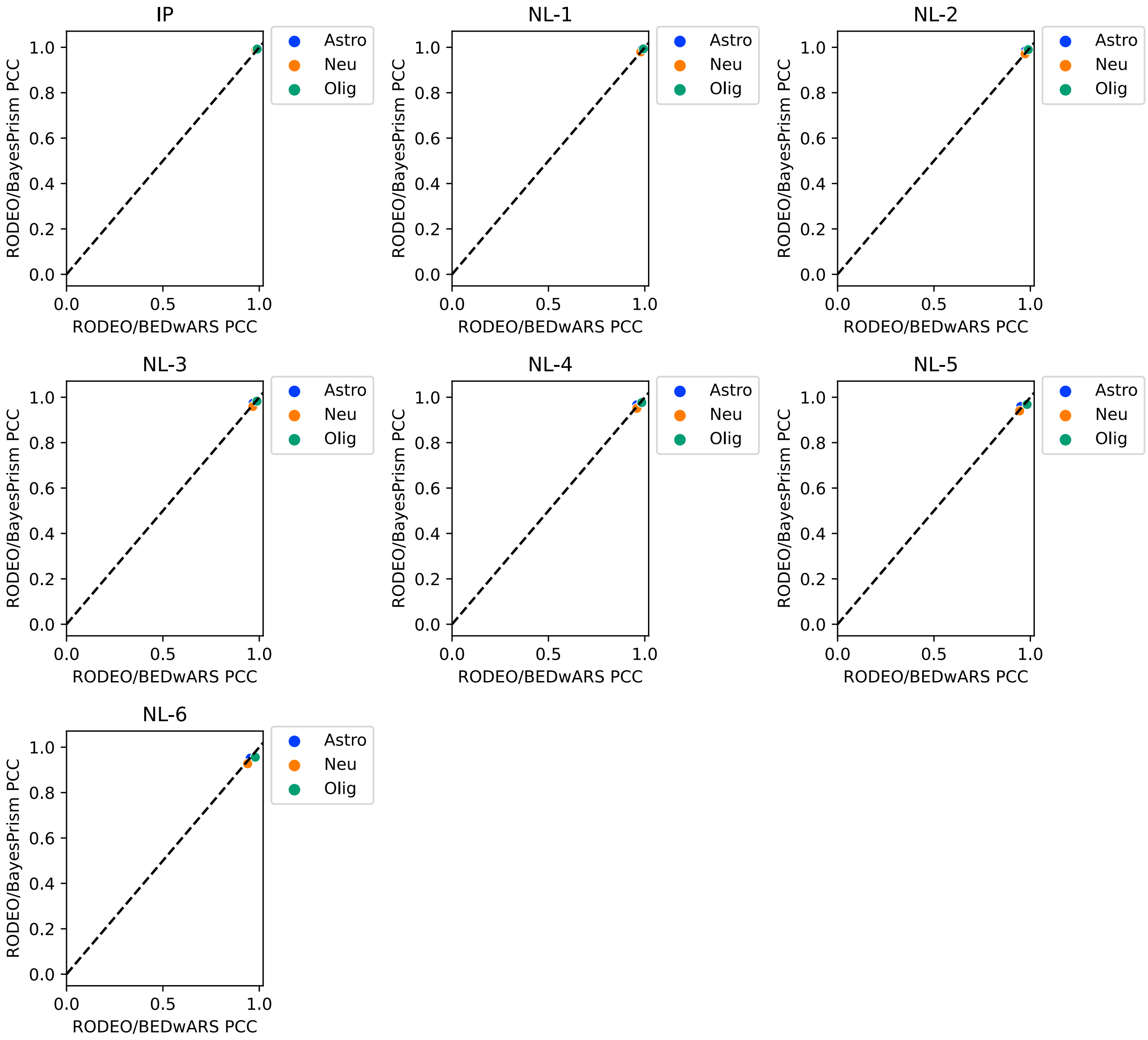

Figure S20. Cell type level comparison of RODEO/BayesPrism and RODEO/BEDwARS for the task of signature estimation by deconvolution of Darmanis pseudo-bulk samples. Different panels correspond to different reference signatures used during deconvolution -- the IP signature and its noisy variants NL-1, NL-2, … NL-6. Performance is measured by the Pearson Correlation Coefficient (PCC) between estimated and true signatures of a cell type. For evaluations with noisy signatures, the average over 11 noisy signatures at the same noise level is shown. In all cases RODEO/BEDwARS and RODEO/BayesPrism have similar performance.

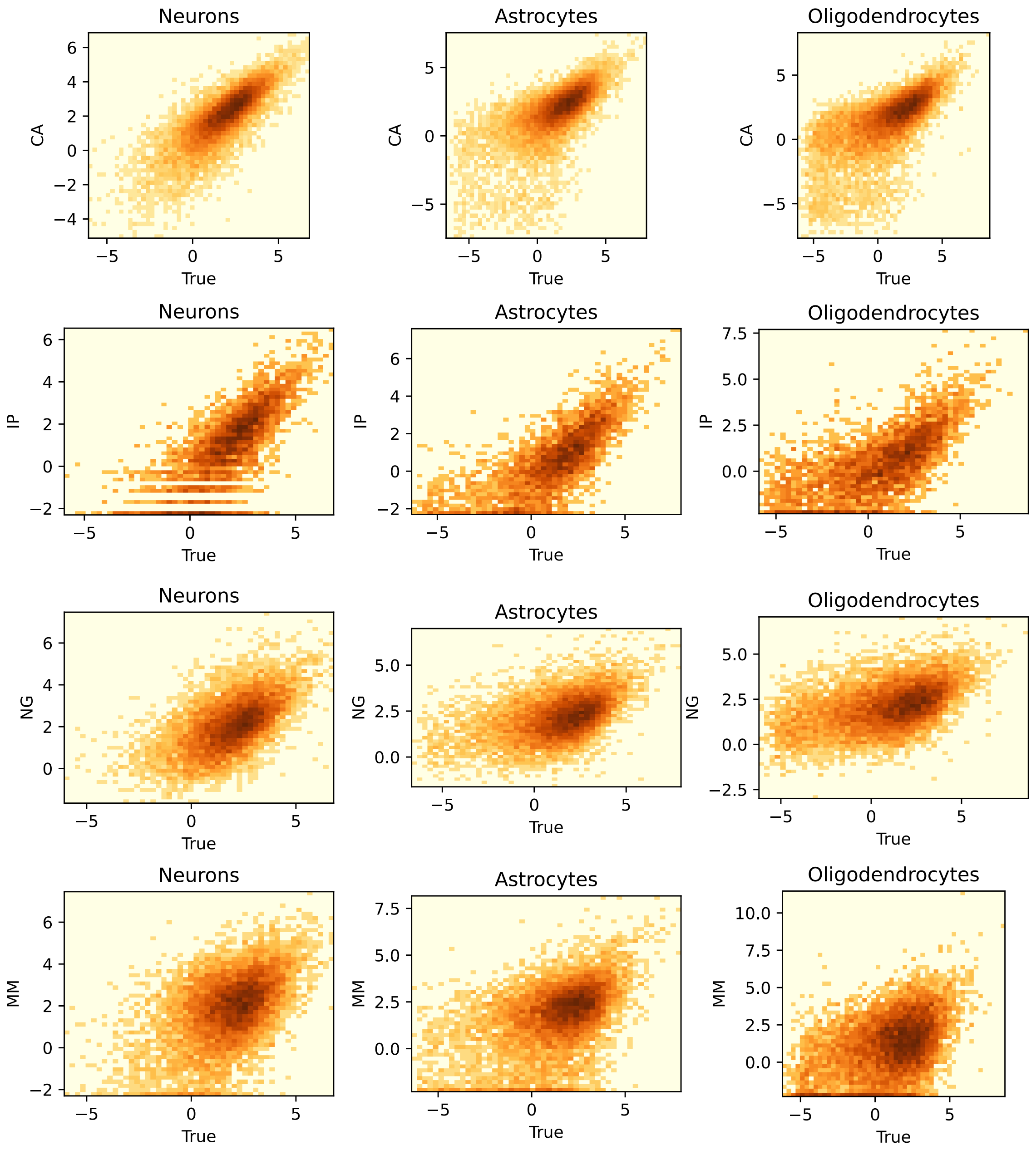

Figure S21. Relationship of different brain reference signatures with the true Darmanis signature. For each reference signature (CA, IP, NG, and MM), each gene's expression value in that signature is shown on the y-axis and its expression in the true signature (Darmanis) is shown on the x-axis; both are in log scale. CA is the most similar to the Darmanis signature whereas MM is the most dissimilar one. The MM signature is generated from mouse brain gene expression. NG signature is also highly diverged from the true signature, which may be attributed to it being derived from a different region (prefrontal cortex) of brain than the Darmanis (temporal middle gyrus) signature.

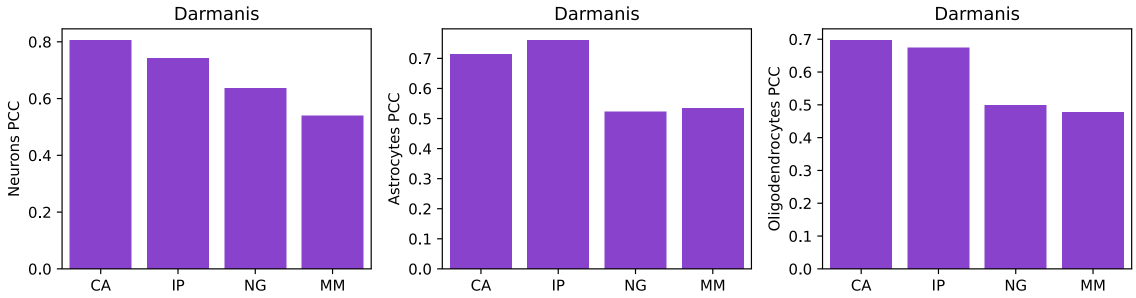

Figure S22. Correlation between the true Darmanis signature and reference brain signatures from multiple sources. For each cell type, Pearson correlation coefficient (PCC) is shown between the log-transformed reference and true signatures. Overall, CA and MM are the most similar and dissimilar respectively to the Darmanis signatures. CA: adult single-nuclease RNA-seq of human brain from Cell Atlas, IP: RNA-seq data on immune-purified cells from human brain, NG: single-nuclease RNA-seq of human brain (Nagy et al.), MM: RNA-seq data on immune-purified mouse brain.

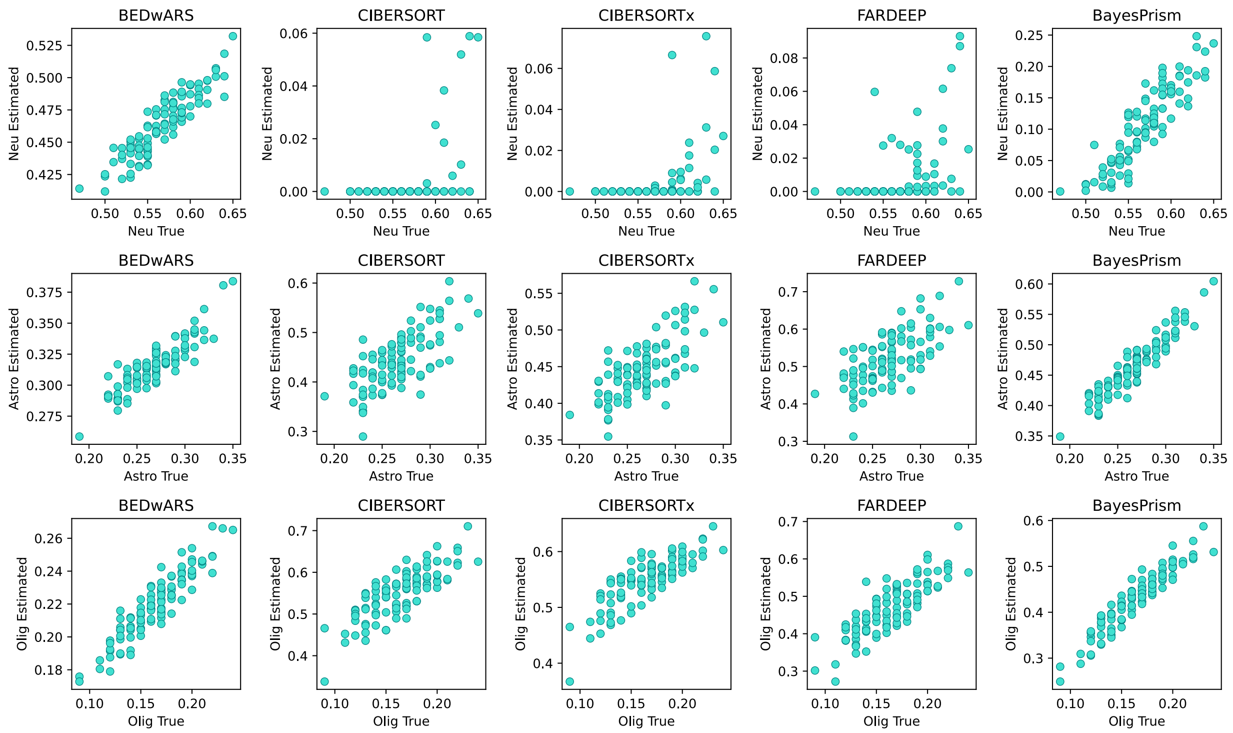

Figure S23. Quality of cell type proportion inference using NG signature in deconvolving pseudo-bulk samples from Darmanis. The estimated and true proportions for each of 100 pseudo-bulk samples are compared, for all cell types and all methods (BEDwARS, CIBERSORT, CIBERSORTx, FARDEEP, BayesPrism). All methods except BEDwARS fail to predict neuron proportion for most of the samples. BayesPrism underestimates neurons proportions by at least a factor of 2.5 and 10 for higher and lower proportions, respectively. For astrocytes, the estimated values of the highest proportions are almost twice as large as true values, for all methods except BEDwARS. The same holds for oligodendrocytes, where other methods overestimate the highest proportions by ~ 3-fold. Note that the NG signatures are derived from a different region of brain than the Darmanis signatures.

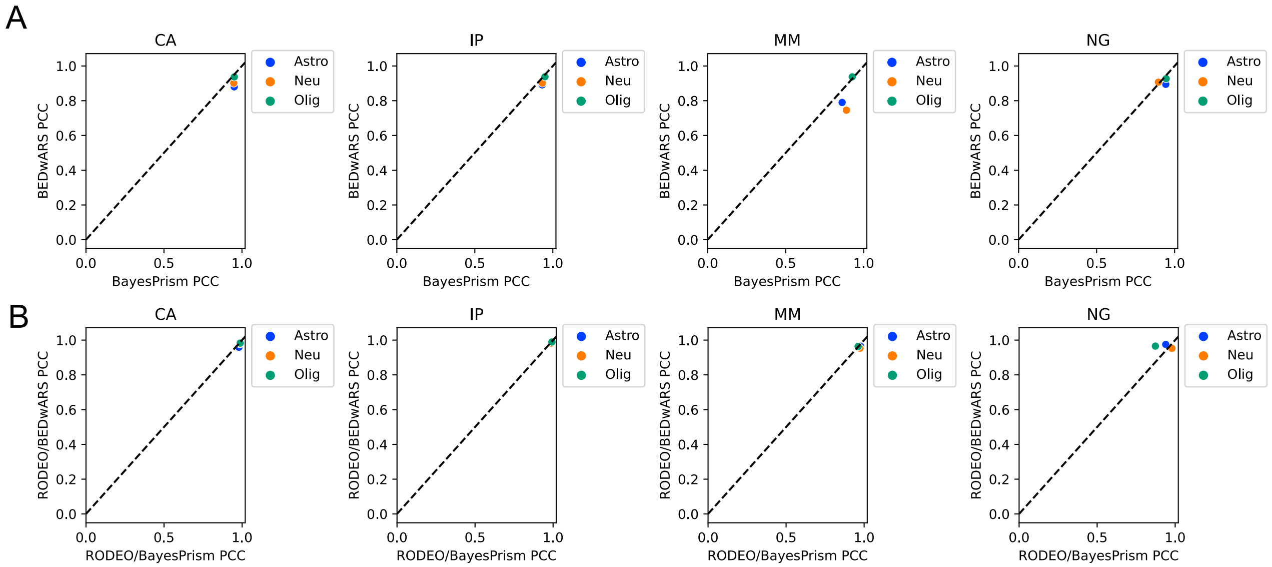

Figure S24. Cell type level comparison of (BEDwARS, BayesPrism) and (RODEO/BEDwARS, RODEO/BayesPrism) for proportion and signature estimation by deconvolution of Darmanis pseudo-bulk samples using multiple brain reference signatures. Different panels correspond to different reference signatures used during deconvolution -- CA, IP, MM and NG. **A.** Performance is measured by the Pearson Correlation Coefficient (PCC) between estimated and true proportions of a cell type, across the 100 pseudo-bulk samples. Performance of BEDwARS and BayesPrism are mostly similar in estimating cell type proportions. **B.** PCC is computed between estimated and true signatures of a cell type for signature estimation. Performance of RODEO provided with BEDwARS-estimated proportions is slightly better than RODEO/BayesPrism for NG signature.

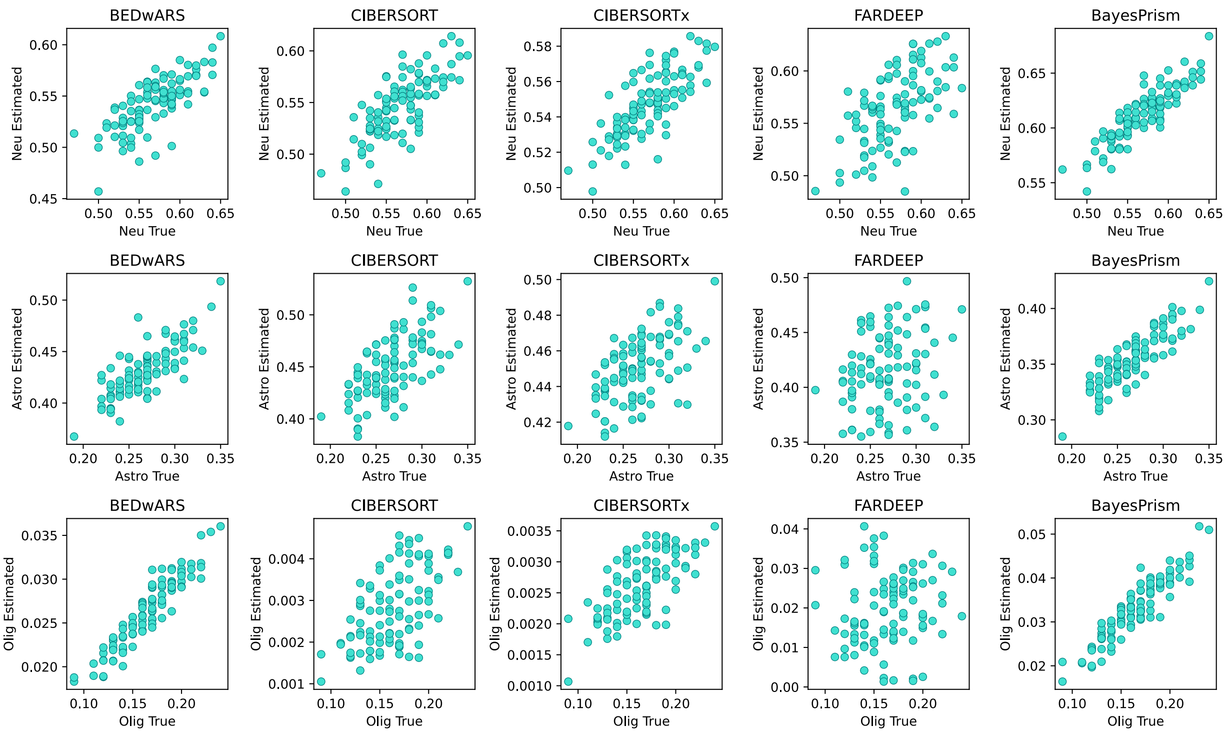

Figure S25. Quality of cell type proportion inference using MM signature in deconvolving pseudo-bulk samples from Darmanis. The estimated and true proportions for each of 100 pseudo-bulk samples are compared, for all cell types and all methods (BEDwARS, CIBERSORT, CIBERSORTx, FARDEEP, BayesPrism). Overall BEDwARS and BayesPrism-estimated proportions are better than other methods in capturing the trend of true proportions, especially for oligodendrocytes. While the maximum estimated proportion is ~10-fold (BEDwARS and FARDEEP) and ~100-fold (CIBERSORT(x)) less than the true proportion, BEDwARS and BayesPrism estimates have significantly higher correlation for this cell type. A cell type signature estimation method such as RODEO does benefit from estimations highly correlated with true values even if the absolute values do not match perfectly.

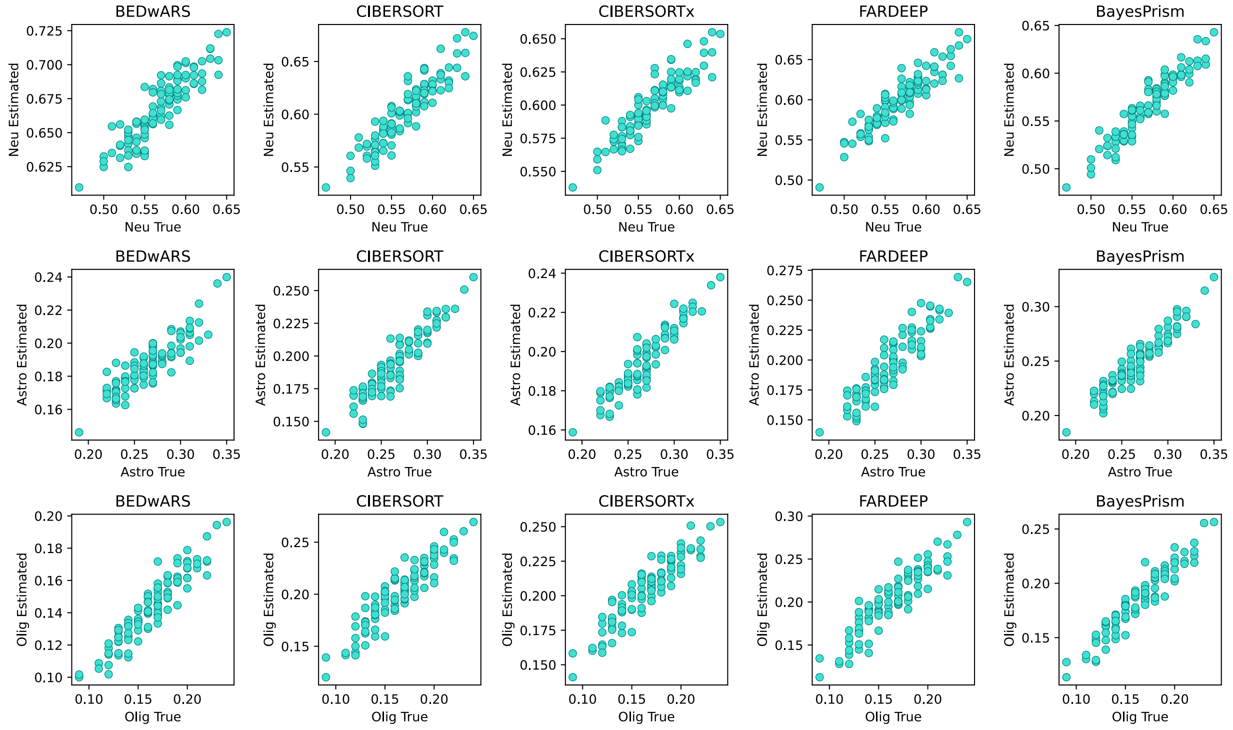

Figure S26. Quality of cell type proportion inference using CA signature in deconvolving pseudo-bulk samples from Darmanis. The estimated and true proportions for each of 100 pseudo-bulk samples are compared, for all cell types and all methods (BEDwARS, CIBERSORT, CIBERSORTx, FARDEEP). All methods perform almost equally well in estimation of proportions.

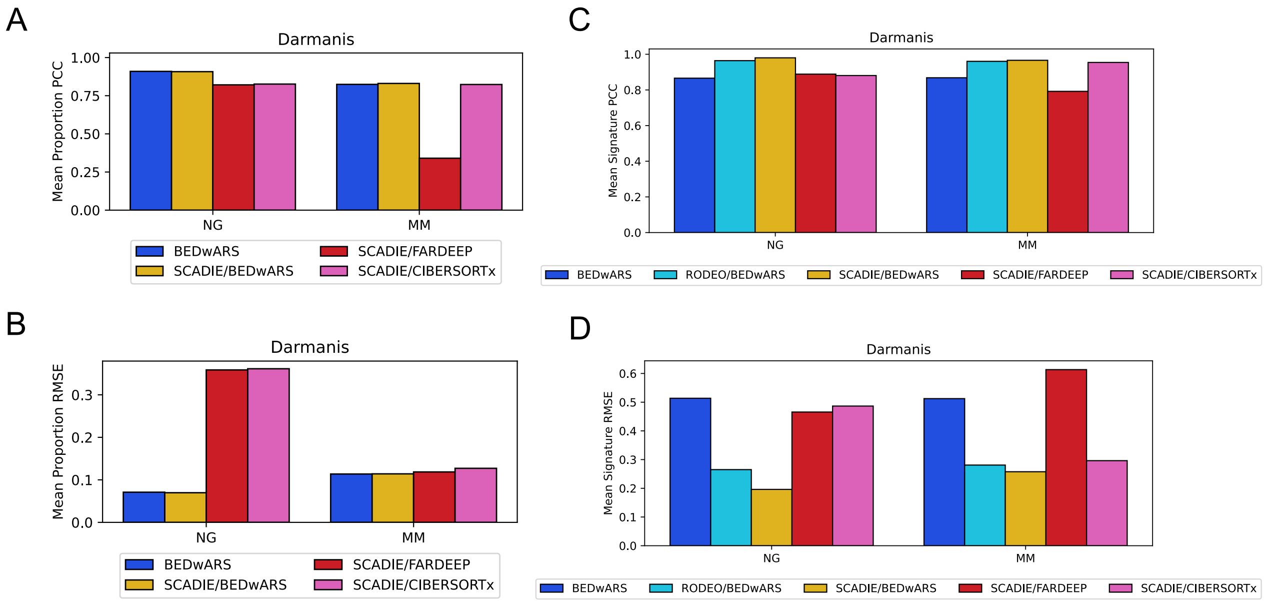

Figure S27. Evaluation of cell type proportion and signature estimation by Pearson Correlation Coefficient and RMSE for SCADIE. Pseudo-bulk profiles of Darmanis dataset were deconvolved with SCADIE using two reference signatures – NG and MM. Performance of SCADIE was evaluated with three different deconvolution methods – BEDwARS, FARDEEP and CIBERSORTx – in its first step, and compared with BEDwARS for cell type proportion (**A, B**) and with BEDwARS and RODEO/BEDwARS for cell type signature estimation (**C, D**). For the estimation of cell type proportion with NG signatures, SCADIE/BEDwARS is competitive with BEDwARS but SCADIE/FARDEEP and SCADIE/CIBERSORTx are not competitive. For the estimation of cell type signatures starting with NG signatures, SCADIE/BEDwARS is competitive with RODEO/BEDwARS but SCADIE/FARDEEP and SCADIE/CIBERSORTx are not. SCADIE's performance in the inference of cell type proportions and signatures is strongly dependent on the deconvolution method used for the initialization of cell type proportions in its first step. For example, FARDEEP shows the lowest quality of proportion estimates by PCC criterion using MM signature (**Figure 4C)** and SCADIE/FARDEEP (i.e., SCADIE with FARDEEP deconvolution in its first step) likewise has the lowest average correlation for the estimation of cell type proportions (A). Similarly, BEDwARS has the highest quality of cell type proportion estimates by both PCC and RMSE criterion using NG signature, and SCADIE initialized with BEDwARS-estimated proportions has similar performance to BEDwARS (A).

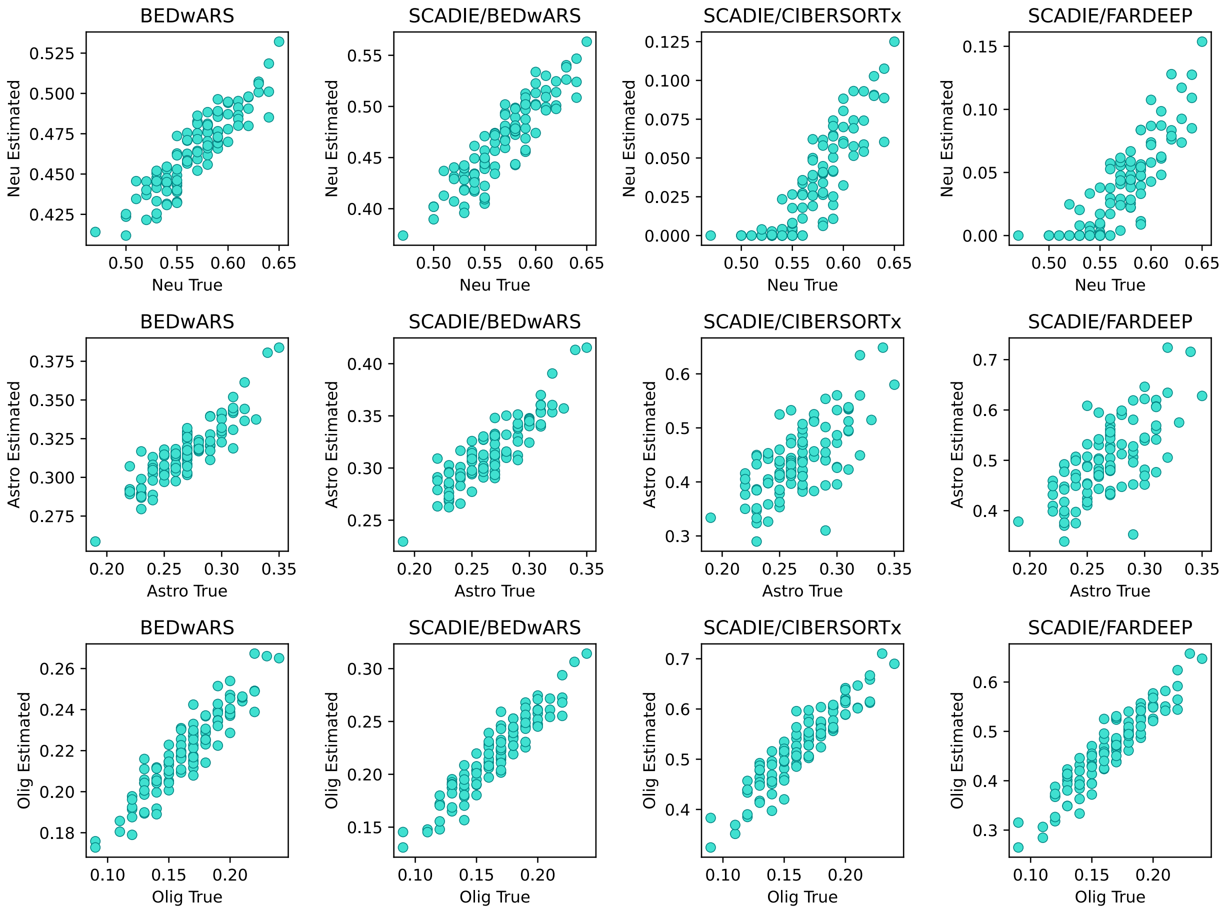

Figure S28. Quality of cell type proportion inference using NG signature in deconvolving pseudo-bulk samples from Darmanis for BEDwARS and SCADIE. SCADIE was evaluated with three different deconvolution methods – BEDwARS, CIBERSORTx, FARDEEP – for its first step (denoted by SCADIE/BEDwARS, SCADIE/CIBERSORTx, SCADIE/FARDEEP). The estimated and true proportions for each of 100 pseudo-bulk samples are compared, for all cell types and methods. Quality of estimated proportions by SCADIE are affected by the deconvolution method used for the initialization of cell type proportions. In astrocytes and oligodendrocytes SCADIE estimated proportions are almost two and three times larger than the true proportions when initialized with CIBERSORTx and FARDEEP. Neuron proportions are extremely underestimated as well. Similar patterns exist in **Figure S23** for CIBERSORTx and FARDEEP. BEDwARS and SCADIE/BEDwARS have similar quality of estimation and show the most accurate estimates for all cell types.

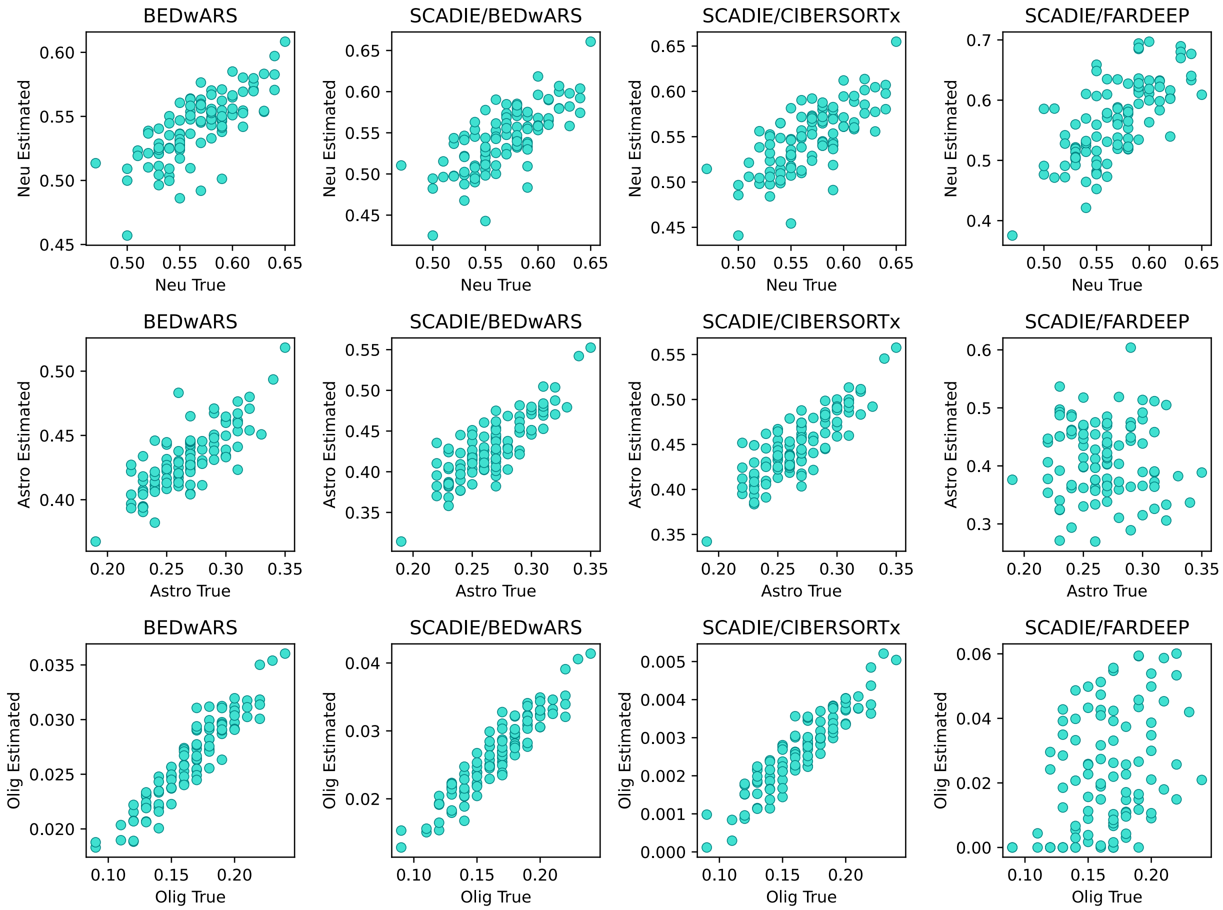

Figure S29. Quality of cell type proportion inference using MM signature in deconvolving pseudo-bulk samples from Darmanis for BEDwARS and SCADIE. The estimated and true proportions for each of 100 pseudo-bulk samples are compared, for all cell types and variants of SCADIE as well as BEDwARS. Quality of estimated proportions by SCADIE are affected by the deconvolution method used for the initialization of cell type proportions. For oligodendrocytes and astrocytes, the estimated proportions are poorly correlated with true correlations when FARDEEP is used for initialization. When initialized with CIBEROSRTx-estimated proportions the final proportions of oligodendrocytes reported by SCADIE are 100 times less than the true proportions even though they are highly correlated with each other. These observations are similar to the ones in Figure **S25** which confirms that SCADIE's performance is highly dependent on its initialization with other deconvolution methods. SCADIE initialized with BEDwARS has similar quality of estimates to BEDwARS and is more accurate than its other variants.

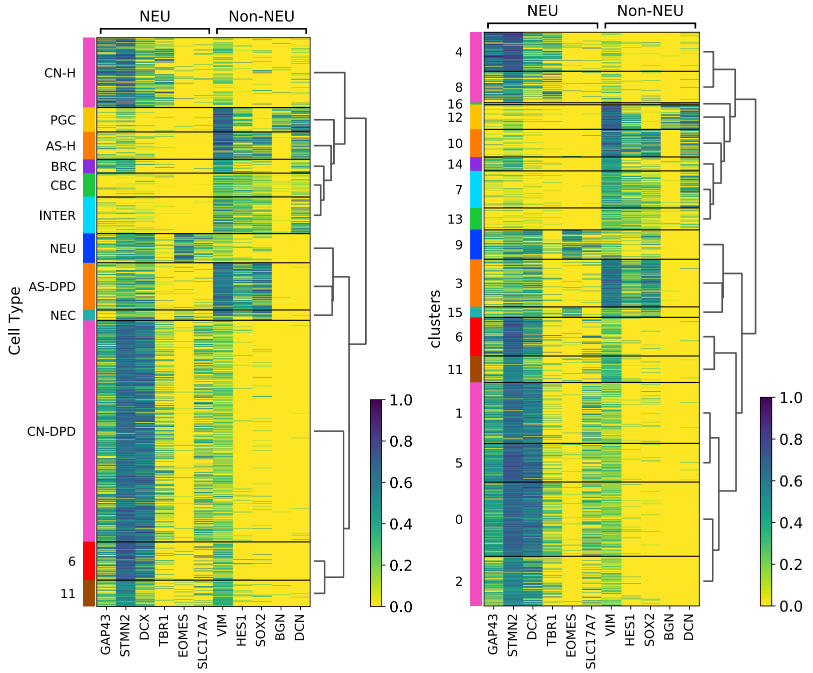

Figure S30. Hierarchical clustering of the cells from DPD-deficient patient and the non-affected individual. The standardized/scaled expression (divided by max in each dimension to fall in (0, 1) interval) of neuronal (GAP43, STMN2, DCX, TBR1, EOMES, SLC17A7) and non-neuronal (VIM, HES1, SOX2, BGN, DCN) gene markers are shown for the cells of DPD-deficient patient and non-affected individual. Hierarchical clustering was performed on the cells grouped by their assigned cell type (**left**) or their cluster index (**right**). Each cell type and all the cluster indices assigned to it are represented with the same color in both heatmaps. Cells from both individuals were pooled into a single set before clustering.

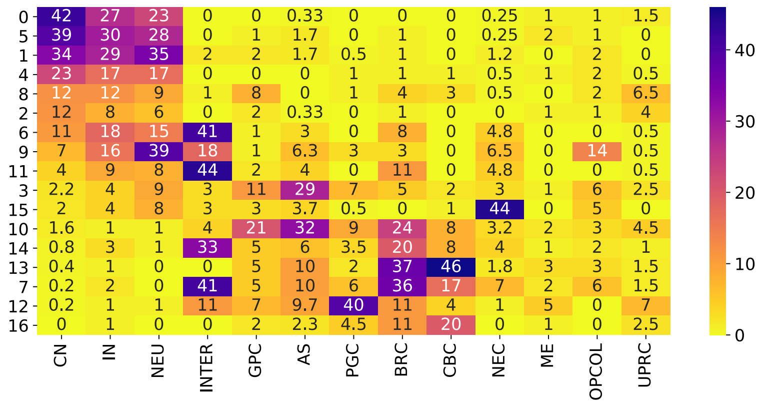

Figure S31. Overlap of statistically derived markers between DPD deficiency dataset and Tanaka et al. (27) study. For each cluster of cells (rows), shown is the overlap of its markers with the markers of a cell type from Tanaka et al. (columns). In cases where a cell type from Tanaka et al. has multiple subtypes such as CN1, CN2, …, CN5, the average overlap over the subtypes was computed.

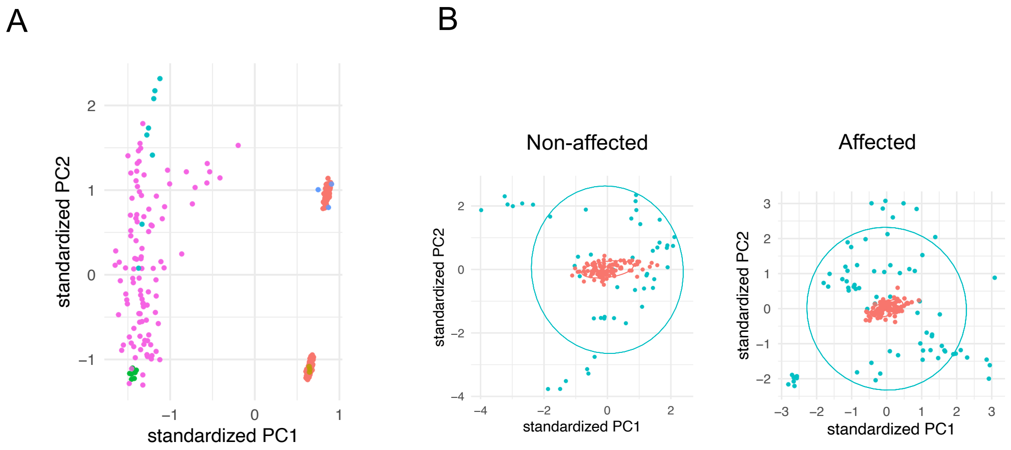

Figure S32. Batch correction for bulk RNA-seq samples of affected and non-affected groups of individuals. A. PCA plot of bulk RNA-seq and pseudo-bulk samples used in the study of DPD deficiency. Points on the left (pink, teal and green) are the bulk RNA-seq samples from non-affected (48) and affected individuals (72). The teal and green points represent the eight semi-matched bulk samples of the non-affected and affected groups. Points on the right are the pseudo-bulk samples for each group. Red points represent pseudo-bulk samples generated by bootstrapping from single cell data for non-affected (top) and affected (bottom) groups. The blue and brown points are the pseudo-bulk samples generated by summing the expression of cells within each organoid. **B.** The bulk samples (teal) of non-affected and affected groups are batch corrected to the bootstrapped pseudo-bulk samples (red) of non-affected and affected groups separately.

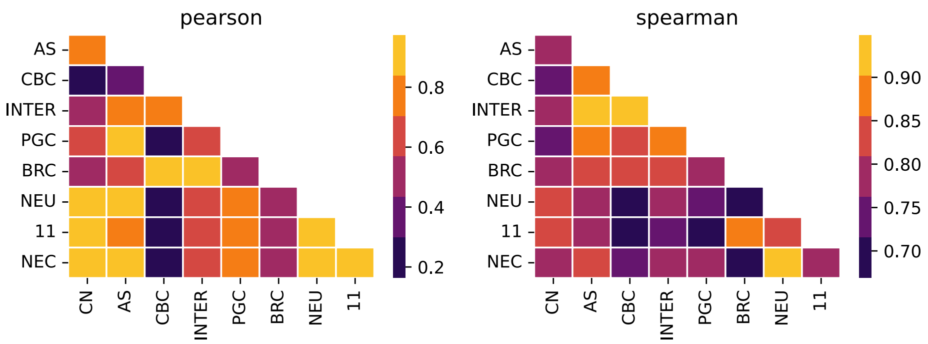

Figure S33. Pairwise similarities of cell type signatures used for deconvolving affected and non-affected bulk samples by DPD deficiency. Pearson (left) and Spearman (right) correlation coefficients are shown for each pair of cell types. CBC has the highest correlation with INTER by both statistics. Similarly, CN is highly correlated with neuron (NEU), NEC, and cluster 11 by both criteria.

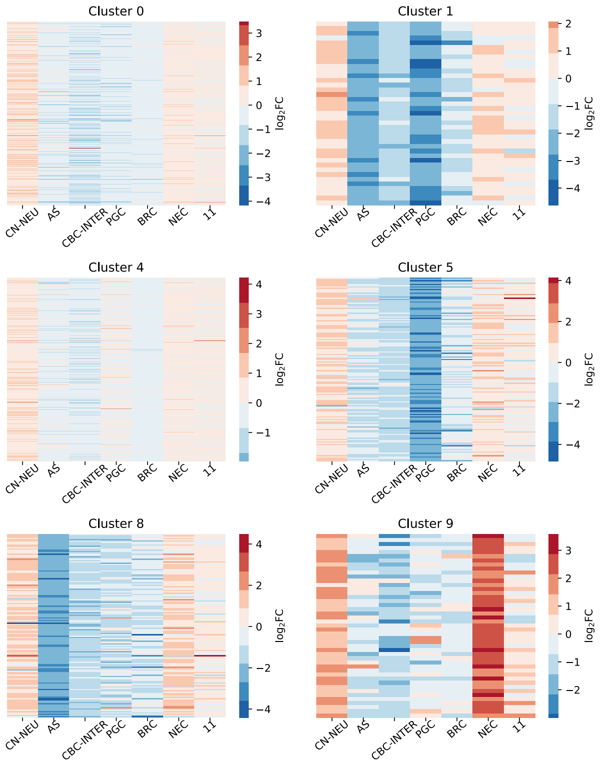

Figure S34. Kmeans clustering of differential expression patterns across cell types. Each gene is assigned a cell type-specific expression fold change between affected and non-affected individuals based on results from BEDwARS deconvolution of bulk RNA-seq data. A gene's (log) fold-change in each of 7 cell types (two of these represent pairs of cell types) is its differential expression pattern. Genes' expression patterns are clustered into ten clusters using K-means. Shown are the differential expression patterns of genes in each resulting cluster.

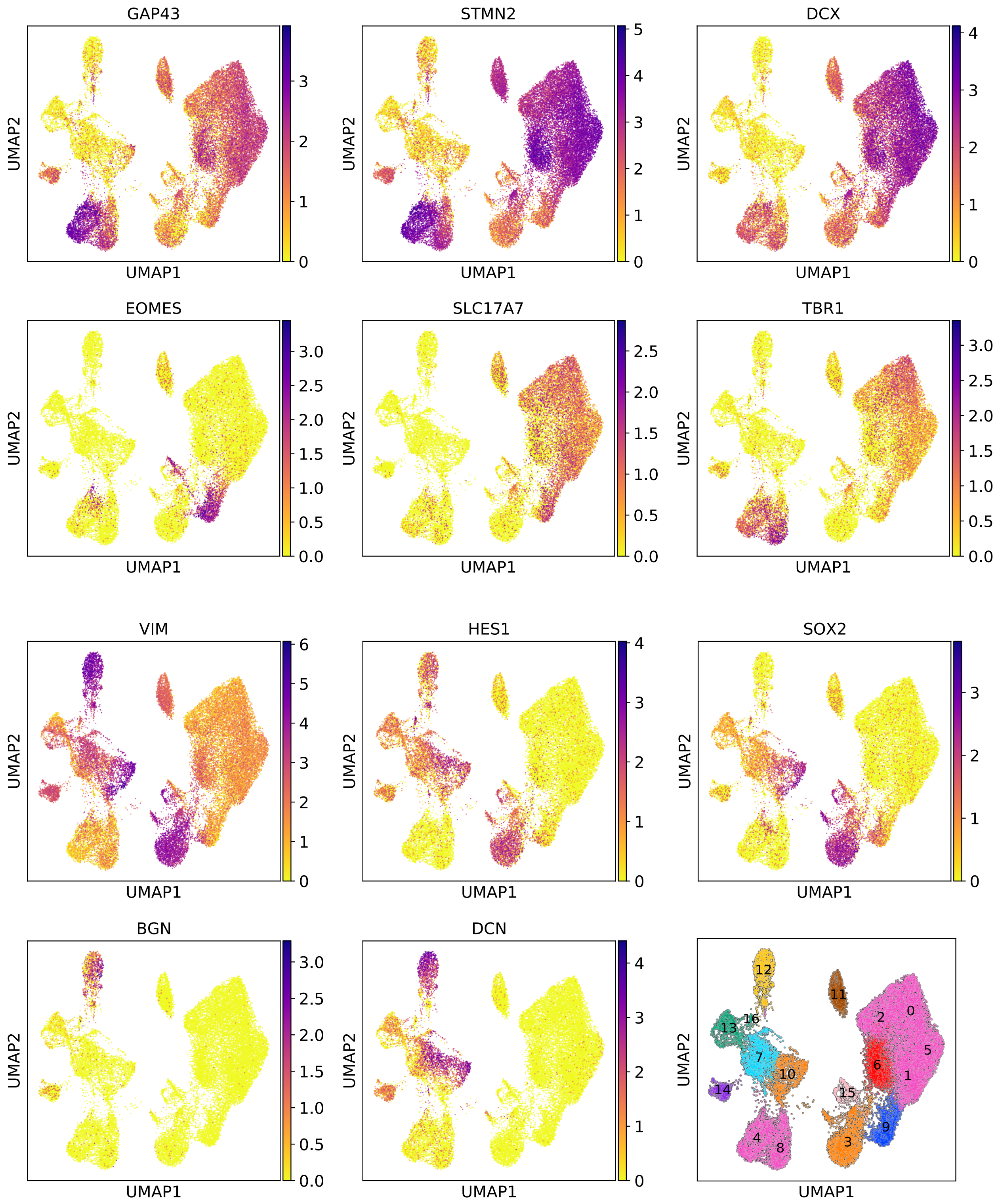

Figure S35. Distribution of the expression of marker genes over the cells. The normalized and log-transformed expression of neuronal (GAP43, STMN2, DCX, TBR1, EOMES, SLC17A7) and non-neuronal (VIM, HES1, SOX2, BGN, DCN) marker genes represented in the UMAP plots

of the DPD-deficient patient and non-affected individual. The last panel shows the cluster indices on cell clusters. Clusters with the same color were assigned to the same cell type.

Cluster Index	Expressed Markers	Marker Overlap	GO Enrichment	Cluster type	Assigned Cell Type
0	CN	CN	-	Affected	CN
1	CN	CN	-	Affected	CN
2	CN	CN	-	Affected	CN
3	Non-neuronal	AS	AS	Affected	AS
4	CN	CN	-	Non-Affected	CN
5	CN	CN	-	Affected	CN
6	CN	INTER	-	Affected	unassigned
7	Non-neuronal	INTER/BRC	-	Non-Affected	INTER
8	CN	CN	-	Non-Affected	CN
9	Neuron	Neuron	-	Affected	Neuron
10	Non-neuronal	AS	AS	Non-Affected	AS
11	Inconclusive	INTER	-	Non-Affected	unassigned
12	PGC	PGC	-	Non-Affected	PGC
13	Non-neuronal	CBC	CBC	Non-Affected	CBC
14	Non-neuronal	INTER/BRC	-	Non-Affected	BRC
15	Non-neuronal	NEC	NEC	Affected	NEC
16	Non-neuronal	CBC	CBC	Non-Affected	CBC

Table S1. Cell type assignment to cell clusters for the DPD deficient patient and the non-affected individual for whom scRNA-seq data were available. Each cluster of cells is assigned to a cell type based on the agreement of at least two out of three criteria used. First criterion (Expressed Markers) is the average expression of neuronal and non-neuronal genes used by Tanaka et al., second criterion (Marker Overlap) is the overlap of the statistically derived markers of our clusters and the cell types of Tanaka et al., and the third criterion (GO Enrichment) is the enrichment of statistically derived markers in relevant GO terms. Cluster type specifies whether most cells belong to the affected individual or non-affected individual.

Table S2. Differential gene expression analysis for DPD deficiency. The summary of differential gene expression analysis using Limma package for bulk, pseudo-bulk, and deconvolved cell type or pairs of cell types (CN-NEU, CBC-INTER) expression profiles (**“DGE”**). Top 200 DE genes per cell type or pairs of cell types are listed in **“Top 200 DE genes per cell type”** sheet**.**

GO:0000785	chromatin
GO:0005576	extracellular region
GO:0005615	extracellular space
GO:0005683	U7 snRNP
GO:0005739	mitochondrion
GO:0005743	mitochondrial inner membrane
GO:0005747	mitochondrial respiratory chain complex I
GO:0005758	mitochondrial intermembrane space
GO:0005783	endoplasmic reticulum
GO:0005788	endoplasmic reticulum lumen
GO:0005794	Golgi apparatus
GO:0005852	eukaryotic translation initiation factor 3 complex
GO:0016282	eukaryotic 43S preinitiation complex
GO:0016607	nuclear speck
GO:0032543	mitochondrial translation
GO:0032870	cellular response to hormone stimulus
GO:0034663	endoplasmic reticulum chaperone complex
GO:0035976	transcription factor AP-1 complex
GO:0042612	MHC class I protein complex
GO:0042824	MHC class I peptide loading complex
GO:0051082	unfolded protein binding
GO:0098869	cellular oxidant detoxification
GO:1990837	sequence-specific double-stranded DNA binding

Table S3. GO term names for the GO IDs in Figure 5E.

Table S4. Summary of David gene set characterization performed on top 200 DE genes identified by DGE analysis for bulk, bootstrapped pseudo-bulk and deconvolved cell type expression profiles for DPD deficiency. In each annotation cluster the first GO with significant FDR (FDR < 0.05), highlighted with yellow, was considered.

Table S5. Cluster of genes identified by Kmeans clustering based on the pattern of genes' differential expression across the cell types for DPD deficiency. Each column represents the genes belonging to the same cluster.

Table S6. Summary of David gene set characterization for cluster of genes identified by Kmeans algorithm based on the pattern of genes' differential expression across the cell types for DPD deficiency. The David results for each cluster are included in a sheet named with the cluster name (Cluster X). Clusters zero and four were excluded as they had more than 2000 genes. David results for top 200 DE genes identified by DGE analysis on bulk and bootstrapped pseudo-bulk are included for convenient comparison.

Table S7. Top 200 markers per cluster of non-affected and affected cells for DPD deficiency. Each column contains the top 200 filtered marker genes for a cluster of cells. These markers were used for assigning cell types to the clusters.

Cluster Index	GO Term	$-\log_{10}(\text{pvalue})$
3	astrocyte differentiation	2.7
10	astrocyte differentiation	4.5
15	mitotic cell cycle	17.7
	mitotic chromosome condensation	10.6
	regulation of mitotic cell cycle	5.8
	regulation of mitotic nuclear division	2.8
13	motile cilium	4
	epithelial cilium movement	3.6
16	cilium movement	9.7
	motile cilium	7.6
	cilium assembly	4.7
	motile cilium assembly	4.3

Table S8. Enrichment of statistically derived cell cluster markers for gene ontology terms used by Tanaka et al. in cell type assignment. Significance of the GO terms enriched in top 200 statistically derived marker genes for a subset of cell clusters from affected and non-affected individuals. The enrichment of these GO terms was used in assigning cell type to clusters.

Table S9. KnowEng gene set characterization performed on the markers of a subset of cell clusters for DPD deficiency. These results were used to assign cell types to cluster of non-affected and affected cells. The GO terms that were enriched and used for cell type assignment are summarized in **Table S8** for easier lookup.

Method	Run Specifications	Time	Memory
BayesPrism	5 threads	60 mins	1 Gb
BayesPrism	20 threads	40 mins	1 Gb
CIBERSORTx	-	65 mins	630 Mb
BEDwARS	150 chains	7 hrs	150 Mb

Table S10. Comparison of the run time and memory requirements for deconvolution of Segerstolpe-T2D dataset with Baron signatures. One and two Intel(R) Xeon(R) CPUs X5650 @ 2.67GHz were used for CIBERSORTx and BayesPrism, respectively. One Tesla V100 GPU was used for BEDwARS.

Supplementary Note 1.

Part A. We examined the histograms of cell type proportions for the benchmark datasets on pancreas and brain. In all pancreatic datasets, for each cell type there are samples where the cell type's proportion is less than 0.1 (10%) (**Table S11, Figure S36A,B,C**). In the brain benchmark (Darmanis dataset) presence of rare cell types is not as frequent as the benchmark on pancreas (**Figure S36D**). Thus, we performed a closer examination of results on the pancreas benchmarks from the point of view of rare cell types.

We compared the performance of BEDwARS to BayesPrism (the second-best method in our benchmarking, added in this revision) in recovering the proportion of cell types for pseudo-bulk samples containing at least one rare cell type (having nonzero proportion less than 0.1) (group "Has-Rare") as opposed to pseudo-bulk samples that do not have any rare cell types (Group "No-Rare"). We computed the PCC and RMSE between estimated and true proportions of each cell type, for each group. For both metrics, BEDwARS performance is similar between the two groups in all datasets and all cell types except for the ductal cell type RMSE in Segerstolpe-H and Segerstolpe-T2D datasets (**Figure S37**). In comparison, there is a more pronounced mismatch between the two groups (Has-Rare and No-Rare) in tests of BayesPrism (**Figure S38**), suggesting that the presence of rare cell types has a greater effect on this method. **Figures S37** and **S38** also illustrate the generally superior performance of BEDwARS over BayesPrism, regardless of the presence of rare cell types. For instance, BayesPrism shows significantly higher RMSE (than BEDwARS) in both groups, for beta, delta and acinar cell types in Segerstolpe-H and Segerstolpe-T2D datasets. Also, RMSE of BayesPrism is significantly higher than BEDwARS for both groups, for ductal cell type in Segerstolpe-T2D and for beta and delta cell types in Enge-H.

To take a closer look at BEDwARS estimates of rare cell types' proportions, for each pancreatic dataset we replotted the estimated vs true proportions for all cell types and all methods at a greater resolution of 0-0.2 for both axes. **Figures S39**, **S40** and **S41** are "zoomed-in" versions of **Figures S2, S8, and S10**. Overall, BEDwARS estimates match the true proportions in magnitude better than the other methods as the scatter plots of BEDwARS are more aligned with the diagonal (identity) line. BayesPrism shows severe underestimation of beta cell type proportions by a factor of ~2 and overestimation of delta and gamma in all three datasets. Also, all methods except

BEDwARS underestimate the acinar cell type proportions by at least 2 fold in Segerstolpe-H (**Figure S39**) and Segerstolpe-T2D (**Figure S40**).

In summary, BEDwARS estimates the rare cell type proportions better than the other methods in the benchmark on pancreatic datasets.

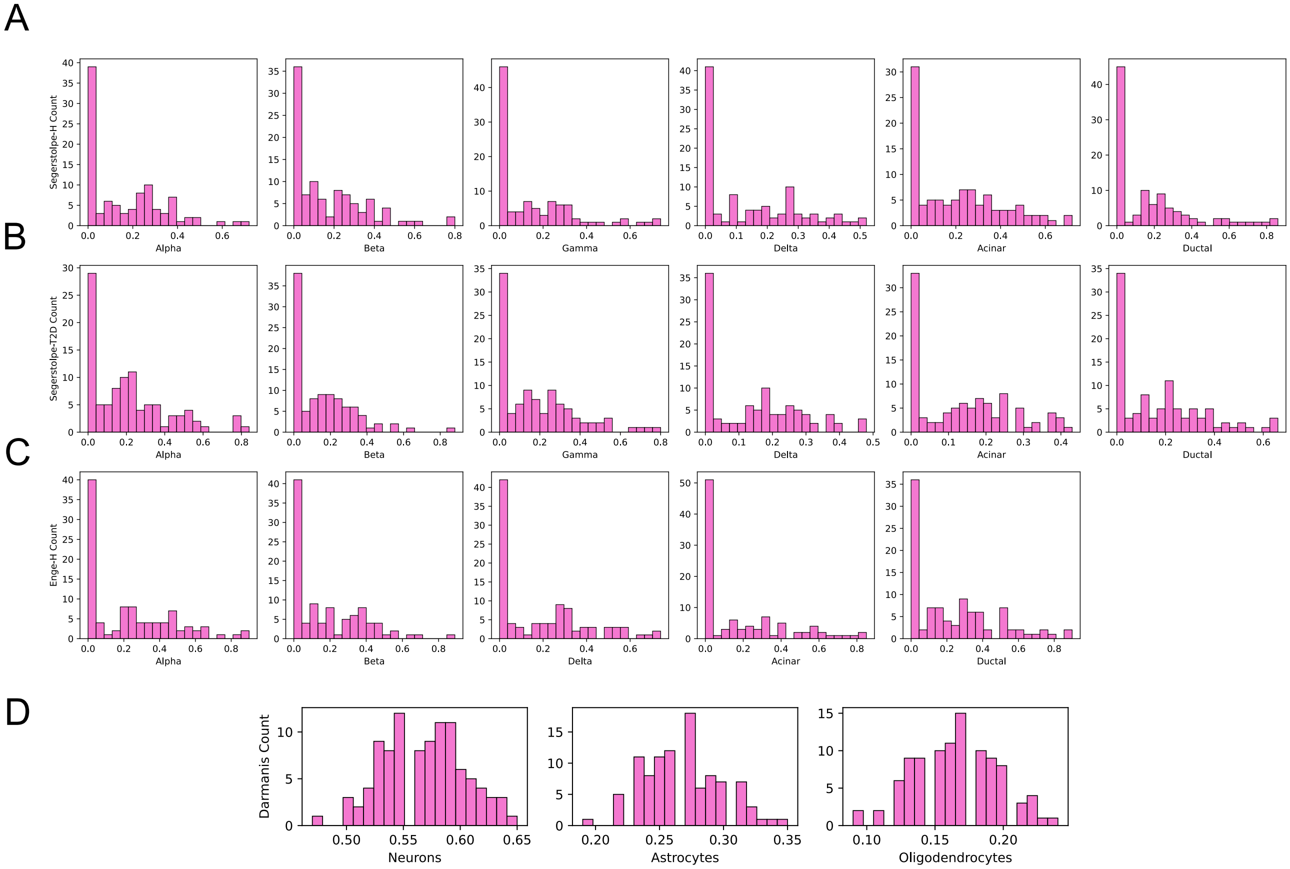

Figure S36. The histogram of cell type proportions for the pseudo-bulk mixtures used in the pancreas (A-C) and brain (D) benchmarks.

Dataset	Segerstolpe-H	Segerstolpe-T2D	Enge-H
Rare Ratio (%)	38	44	17

Table S11. The proportion of pseudo-bulk samples containing at least one rare cell type (cell type proportion <= 10%), in each pancreatic dataset.

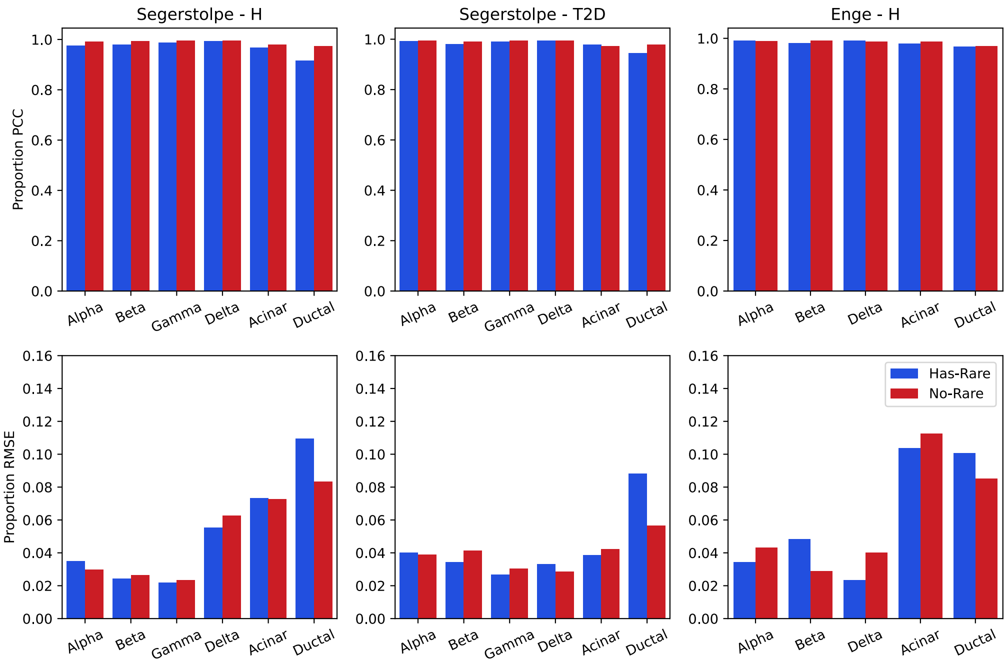

Figure S37. BEDwARS performance comparison between pseudo-bulk samples with ("Has-Rare") or without ("No-Rare") rare cell types.

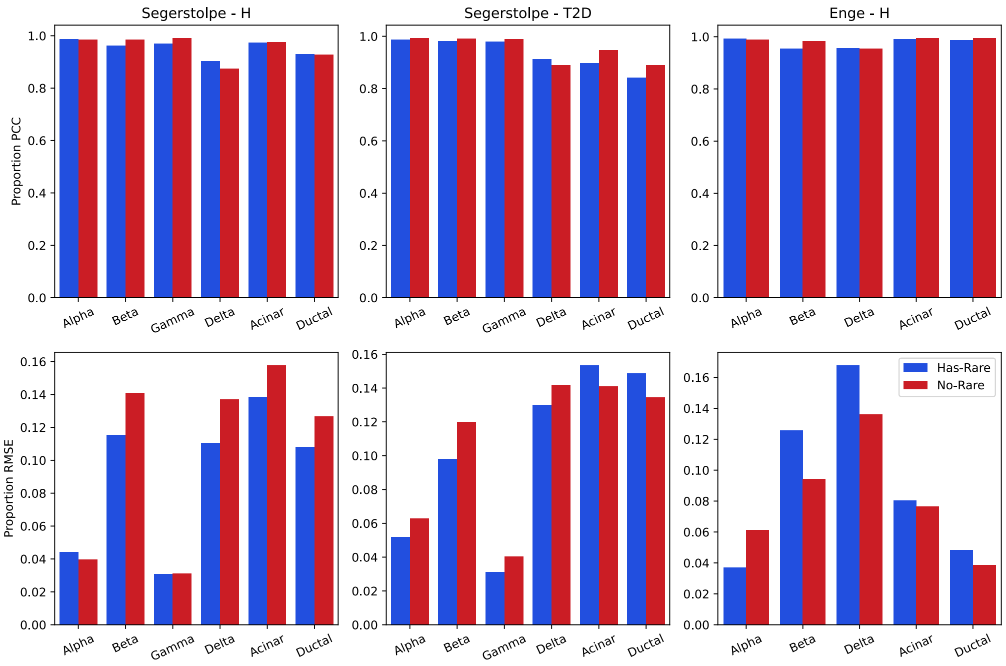

Figure S38. BayesPrism performance comparison between pseudo-bulk samples with ("Has-Rare") or without ("No-rare") rare cell types.

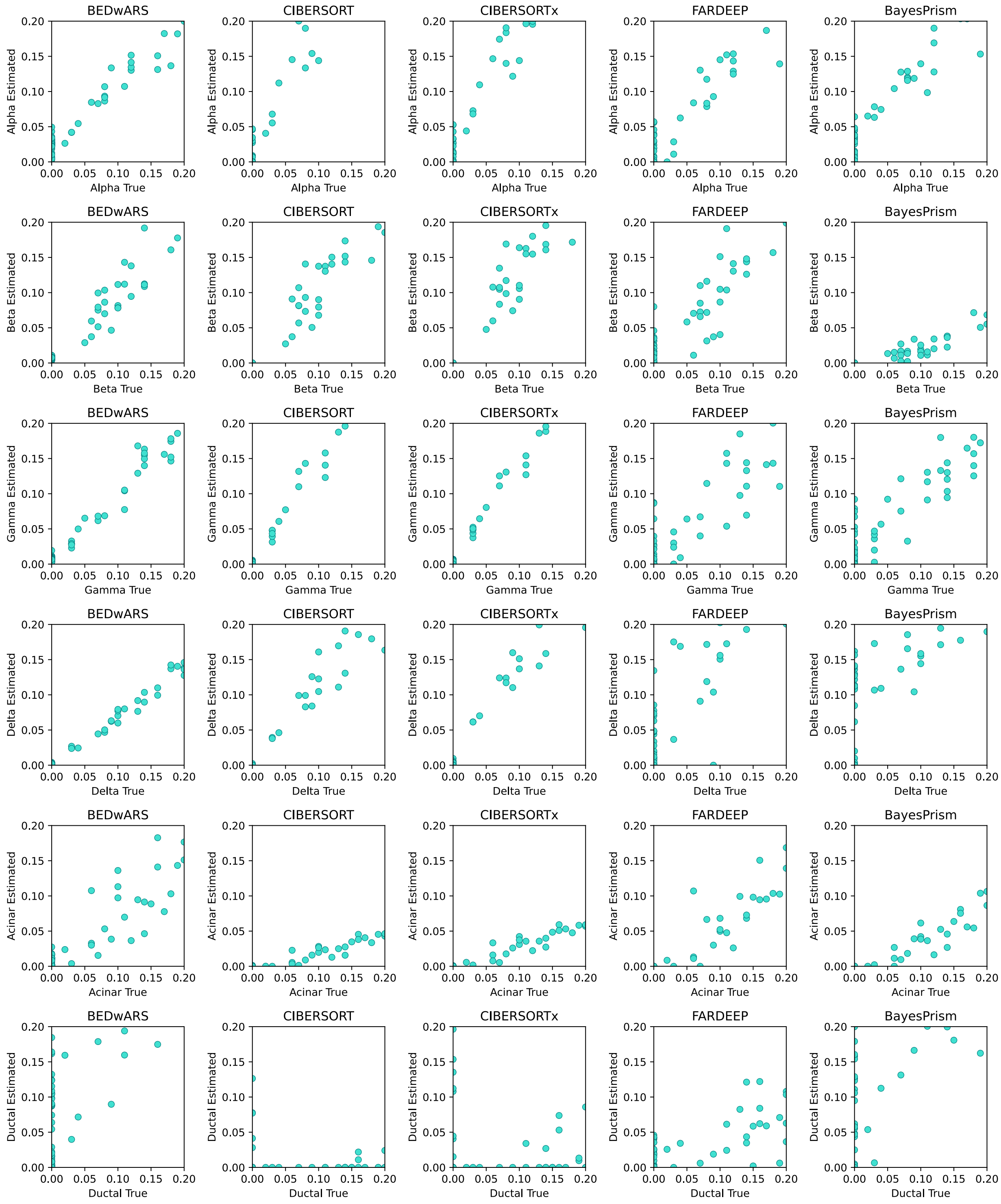

Figure S39. Quality of cell type proportion inference for small proportions (0-0.2) using Baron signature in deconvolving pseudo-bulk samples from Segerstolpe-H. This Figure is revisualization of Figure S2 focused on small proportions.

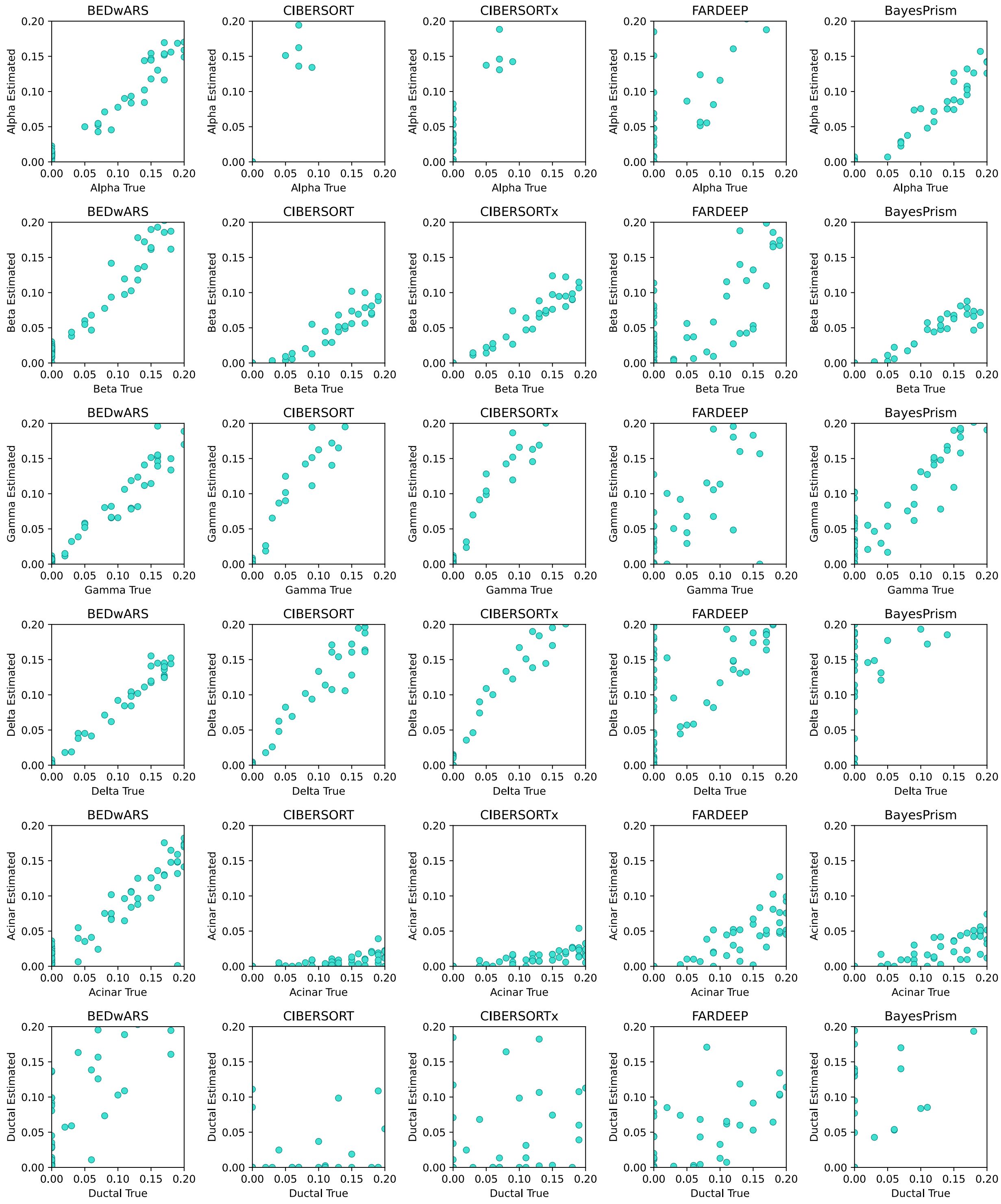

Figure S40. Quality of cell type proportion inference for small proportions (0-0.2) using Baron signature in deconvolving pseudo-bulk samples from Segerstolpe-T2D. This Figure is revisualization of Figure S8 focused on small proportions.

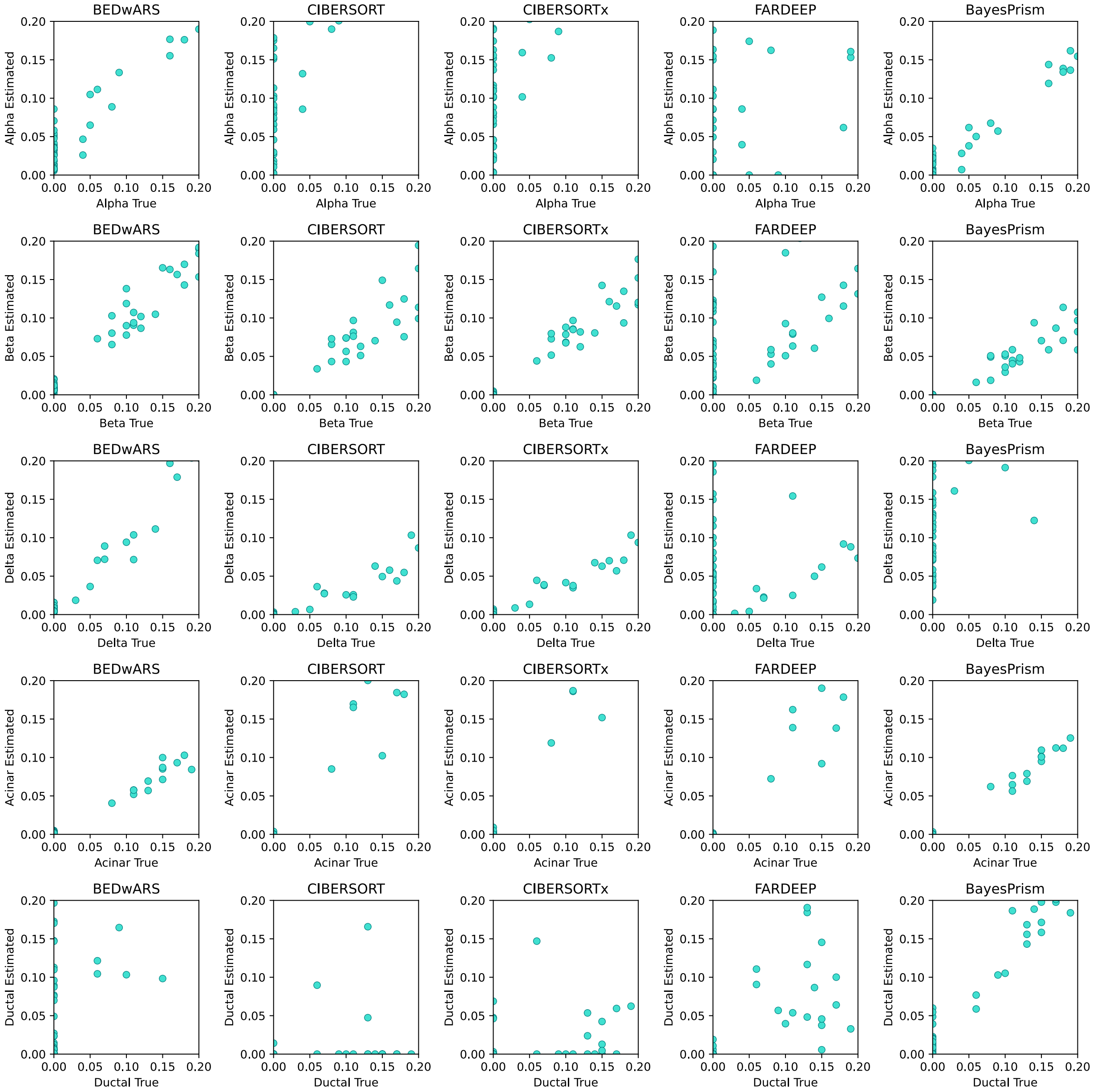

Figure S41. Quality of cell type proportion inference for small proportions (0-0.2) using Baron signature in deconvolving pseudo-bulk samples from Enge-H. This Figure is revisualization of Figure S10 focused on small proportions.

Part B. To study the performance of BEDwARS in deconvolution of heterogenous bulk population we constructed a third dataset (named “Segerstolpe-MIX”) consisting of 50 pseudo-bulk samples from Segerstolpe-H and 50 pseudo-bulk samples from Segerstolpe-T2D. We then performed deconvolution on Segerstolpe-MIX dataset using Baron signatures. **Figure S42A** shows the performance comparison by PCC and RMSE metrics averaged over the 50 Segesrtolpe-H pseudo-bulk samples that were deconvolved in homogenous (Segerstolpe-H) vs heterogeneous (Segerstolpe-MIX/H) settings. The same performance comparison is shown for Segerstolpe-T2D in **Figure S42B.** The deconvolution performance of Segerstolpe-H pseudo-bulk mixtures is similar for all cell types in heterogenous and homogeneous settings by both metrics, whereas the deconvolution of Segerstolpe-T2D pseudo-bulk mixtures is worsened in heterogenous setting for acinar and ductal cell types by both metrics. For beta and delta cell types, the deconvolution performance is affected as per RMSE metric only. In summary, BEDwARS performance is by and large robust to violation of the homogeneity assumption, as per our evaluations.

The observed occasional deterioration in performance is expected as the underlying assumption of BEDwARS is a shared signature among all pseudo-bulk samples. Accommodating for higher granularity of signature adjustment, e.g., sample-specific signatures, is a more challenging problem, which is left for future work.

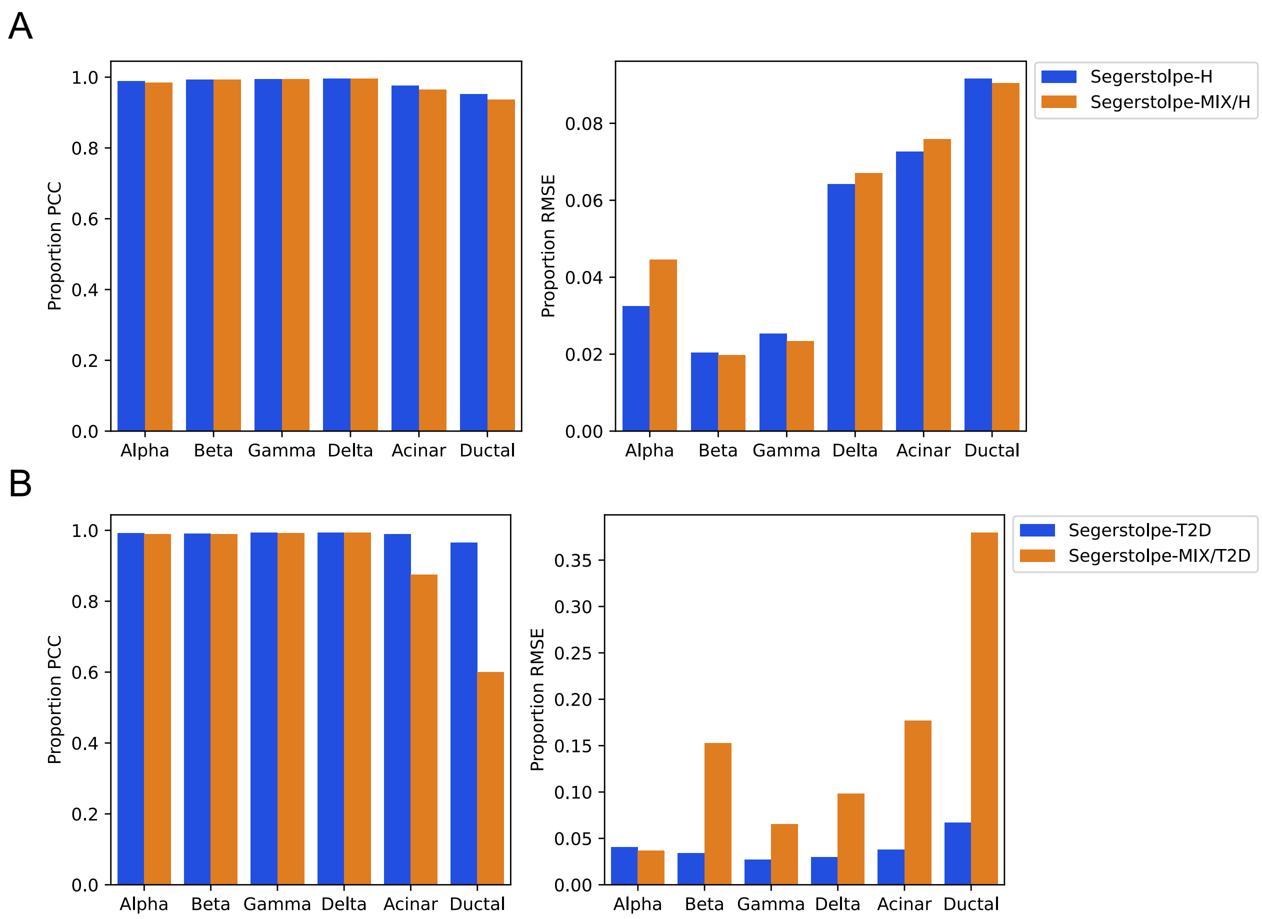

Figure S42. Deconvolution performance comparison between homogenous and heterogenous settings on Segerstolpe-H and Segerstolpe-T2D datasets. In the heterogeneous setting the bulk samples provided to the deconvolution method are a mix of pseudo-bulk samples from Segerstolpe-H and Segerstolpe-T2D. In either setting, the reported accuracy is for pseudo-bulk samples from Segerstolpe-H only (panel A) or from Segerstolpe-T2D only (panel B).

Part C. To study the effect of sample size, we repeated deconvolution of Segerstolpe-T2D data set using Baron signature with 50 and 25 samples and compared the performance with sample size 100 (original analysis in the paper, **Figure 2B**), using averaged PCC and RMSE per cell type (**Figure S43**). The PCC is not affected by sample size based on PCC metric. Also, except for gamma and beta cell types, the RMSE is not affected by sample size. This experiment suggests that at least 25 samples should be used in presence of six cell types for the deconvolution task. Generalizing from this, we thus recommend users to use datasets with the size at least four times the number of cell types to be deconvolved, as smaller sample sizes may not be well suited for the BEDwARS methodology.

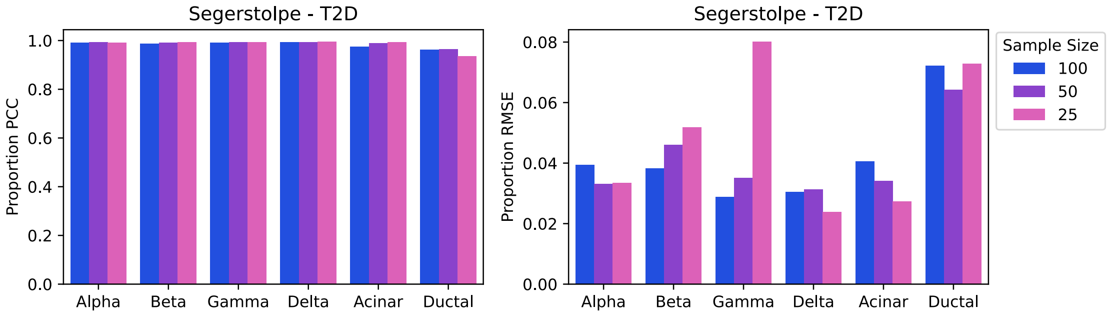

Figure S43. Impact of sample size on the BEDwARS performance. Deconvolution of Segerstolpe-T2D data set using Baron signature with three samples sizes 25, 50, and 100.
