# Supplementary material for: BEDwARS: a robust Bayesian approach to bulk gene expression deconvolution with noisy reference signatures
Source: Genome Biol. 2023 Aug 3;24:178. doi: 10.1186/s13059-023-03007-7 (PMC10399072; doi:10.1186/s13059-023-03007-7)
Supplement: Supplementary file 8 — Additional file 8. The peer review history. [file 13059_2023_3007_MOESM8_ESM.docx]

**1^st^ round**

**Reviewer 1**

Saba Ghaffari et al. developed a Bayesian deconvolution method named BEDwARS specifically designed to address the noisy reference data when deconvolute the bulk transcriptomic profiles in real-world. They have shown advantages of BEDwARS over other state-of-the-art tools for cell type deconvolution using multiple metrics. BEDwARS is shown particularly more robust to noisy reference signatures than all compared methods. Finally, the authors applied BEDwARS to newly generated RNA-seq and scRNA-seq data on over 100 induced pluripotent stem cell-derived neural organoids and identified the possible involvement of ciliopathy and impaired translational control in the etiology of the disorder. In general, I found this study quite interesting, the authors are trying to address an important question in the field and the manuscript is well written. I have a few concerns before considering for publication:

Major concerns:

1. To benchmark BEDwARS over other tools, the authors used single cell RNA-seq data on human pancreatic islets from healthy subjects and generate 100 pseudo-bulk profiles from weighted mixtures of cells of six pre-labeled types - alpha, beta, gamma, delta, acinar, and ductal. These are all major cell types in pancreas, and each of which can be further break down to functionally distinct cell subtypes. It is also quite often that people will find tens of cell subtypes in pancreas, and I am wondering whether BEDwARS still is the best performed tool for cell subtype deconvolution?

2. As we know, the constitutions of cell subtypes can be very different under different conditions. In some organs, the proportions of all cell subtype are very similar, i.e. the distribution of different types of cells are quite even, however, there are cases where some cell type are predominant while others are minority but still functionally important. I am wondering how does the proportion of cell types affect the performance of BEDwARS? Can BEDwARS still accurately identify and correctly predict (compare with other tools) the proportions of rare cell subtypes from bulk cells?

3. The authors chose 3 methods for comparison based on a benchmark study in 2020, however there have been more methods published after 2020, such as BayesPrism (Nature Cancer, 2022). I would suggest the authors compare BEDwARS with more recent published methods.

4. What makes BEDwARS better performed than other tools and why? The authors should at least discuss in the manuscript.

5. The time and memory consumption of each algorithm is also an issue that users are concerned about, and there is a lack of relevant analysis and comparison in the manuscript.

6. Line 539-543, the authors claimed that "However, the proportions of these two individual cell types could not be accurately resolved due to the similarity in the signatures (see Additional File 1: Figure S33). Similar observations were made for the cilia-bearing cells (CBC) and intermediate (INTER) cell type proportions, with their sum matching between bulk-deconvolved and single cell data" This makes me concerned about the performance of BEDwARS in distinguishing similar cell types, which goes to my question 1.

Minor points:

1. In Figure S3 and Figure S17. the citation of FARDEEP, CIBERSORT and CIBERSORTx is missing, simply wrote "[CITE]" in the manuscript.

2. Reference for Tanaka et al. in Figure S31, should be 26 instead of 25.

**Reviewer 2**

Ghaffari et al. present a novel bulk gene expression deconvolution method BEDwARS using Bayesian inference. The authors took single-cell RNA-seq signature matrices as prior and estimated the cell type proportions and the true signature matrix for bulk simultaneously. With an emphasis on differences between reference signatures, Ghaffari et al. improved the performance of cell type proportions estimation even with single-cell reference. The authors demonstrate the accuracy of BEDwARS with simulations under different noise levels and two tissue types: pancreatic islet and brain. The authors also demonstrate the application of BEDwARS to characterize the cell-type specific regulomes of DPD-deficient patients with iPSC.
The manuscript is clear, and the model is rigorous. However, I have a few concerns with the mode and general applicability of the tools. I have provided more details in my comments below:
1. For reference-based deconvolution methods, such as CIBERSORT, MuSiC and Bisque, the deconvolution results for each bulk sample are independent. With the same single-cell reference, the cell type proportions estimated from bulk samples are affected by other bulk samples. However, independence does not hold for BEDwARS. I have two questions:
a. There is an underlying assumption: the bulk expression should come from the homogenous population. Because this homogenous population comes from one true signature matrix. Does the author explore the violation of the homogeneity of the population?
b. How do the sample sizes affect the accuracy of BEDwARS when the samples are from the homogenous population? Intuitively, the larger the sample size, the higher accuracy. How many samples at least do you recommend to achieve better or comparable accuracy with CIBERSORT, CIBERSORTx, and FARDEEP?

2. In the simulation, what do the estimated true signatures look like when they are estimated from different reference signatures? For example, with different noise levels, do the estimated true signature matrices looks the same? Intuitively, the discrepancies between estimated true signature matrices and the true signature matrices are correlated with the noise level.

3. On page 28, the formula at the bottom of the page. Rigorously, it would be better to write as
Θ ̂ = argmax_Θ {Pr⁡(Θ|X)}=argmax_Θ {Pr⁡(X│Θ) Pr⁡(Θ)}

**Authors’ response**

**Reviewer 1:** Saba Ghaffari et al. developed a Bayesian deconvolution method named BEDwARS specifically designed to address the noisy reference data when deconvolute the bulk transcriptomic profiles in real-world. They have shown advantages of BEDwARS over other state-of-the-art tools for cell type deconvolution using multiple metrics. BEDwARS is shown particularly more robust to noisy reference signatures than all compared methods. Finally, the authors applied BEDwARS to newly generated RNA-seq and scRNA-seq data on over 100 induced pluripotent stem cell-derived neural organoids and identified the possible involvement of ciliopathy and impaired translational control in the etiology of the disorder. In general, I found this study quite interesting, the authors are trying to address an important question in the field and the manuscript is well written. I have a few concerns before considering for publication: 
**Response:** We thank the reviewer for their positive feedback and constructive criticism, and we hope our point-by-point response below addresses their concerns.

**Major concerns:
Comment 1:** To benchmark BEDwARS over other tools, the authors used single cell RNA-seq data on human pancreatic islets from healthy subjects and generate 100 pseudo-bulk profiles from weighted mixtures of cells of six pre-labeled types - alpha, beta, gamma, delta, acinar, and ductal. These are all major cell types in pancreas, and each of which can be further break down to functionally distinct cell subtypes. It is also quite often that people will find tens of cell subtypes in pancreas, and I am wondering whether BEDwARS still is the best performed tool for cell subtype deconvolution?

**Response:**

We appreciate the reviewer’s point that distinguishing cell subtypes is of interest. However, we are not aware of a comprehensive benchmark study on comparing deconvolution methods in their ability to address this issue. According to a recent study by Jin et al. (39), current deconvolution methods have difficulty in estimating the proportion of closely related cell types. BEDwARS shares this limitation with other state-of-the-art methods and no aspect of its model makes it particularly robust to problems posed by highly similar subtypes. We plan to address this issue in future versions of BEDwARS. We had included a note about this unsolved problem (faced by existing deconvolution methods) in the last paragraph of the Discussion section.

“Based on our empirical experience as well as on theoretical grounds, we believe BEDwARS may not be able to accurately tease apart the contributions/proportions of highly correlated cell types (4,38,39), a common unsolved problem with deconvolution methods. We also believe that BEDwARS performance can be further improved by modifying it to be more robust to outliers, e.g., by changing the optimization objective or through outlier detection and removal. We leave these important engineering challenges for future iterations of the tool.”

It is worth noting that in some existing deconvolution methods such as CIBERSORT(x), selection of markers (through support vector regression) can in principle help with the above multicollinearity problem; however, in practice this has not proven to be the most effective approach. We show, in the response to the reviewer’s Comment 6 below, that CIBERSORTx estimations are also negatively affected by the similarity of cell types that are abundant in the bulk profiles.

**Comment 2:** As we know, the constitutions of cell subtypes can be very different under different conditions. In some organs, the proportions of all cell subtype are very similar, i.e., the distribution of different types of cells are quite even, however, there are cases where some cell type are predominant while others are minority but still functionally important. I am wondering how does the proportion of cell types affect the performance of BEDwARS? Can BEDwARS still accurately identify and correctly predict (compare with other tools) the proportions of rare cell subtypes from bulk cells?

**Response:**

We thank the reviewer for this insightful comment. To address it, we first examined the histograms of cell type proportions for the benchmark datasets on pancreas and brain. In all pancreatic datasets, for each cell type there are samples where the cell type’s proportion is less than 0.1 (10%) (**Table R1, Figure R1A,B,C**). In the brain benchmark (Darmanis dataset) presence of rare cell types is not as frequent as the benchmark on pancreas (**Figure R1D**). Thus, we performed a closer examination of results on the pancreas benchmarks from the point of view of rare cell types.

We compared the performance of BEDwARS to BayesPrism (the second-best method in our benchmarking, added in this revision) in recovering the proportion of cell types for pseudo-bulk samples containing at least one rare cell type (having nonzero proportion less than 0.1) (group “Has-Rare”) as opposed to pseudo-bulk samples that do not have any rare cell types (Group “No-Rare”). We computed the PCC and RMSE between estimated and true proportions of each cell type, for each group. For both metrics, BEDwARS performance is similar between the two groups in all datasets and all cell types except for the ductal cell type RMSE in Segerstolpe-H and Segerstolpe-T2D datasets (**Figure R2**). In comparison, there is a more pronounced mismatch between the two groups (Has-Rare and No-Rare) in tests of BayesPrism (**Figure R3**), suggesting that the presence of rare cell types has a greater effect on this method. **Figures R2 and R3** also illustrate the generally superior performance of BEDwARS over BayesPrism, regardless of the presence of rare cell types. For instance, BayesPrism shows significantly higher RMSE (than BEDwARS) in both groups, for beta, delta and acinar cell types in Segerstolpe-H and Segerstolpe-T2D datasets. Also, RMSE of BayesPrism is significantly higher than BEDwARS for both groups, for ductal cell type in Segerstolpe-T2D and for beta and delta cell types in Enge-H.

To take a closer look at BEDwARS estimates of rare cell types’ proportions, for each pancreatic dataset we replotted the estimated vs true proportions for all cell types and all methods at a greater resolution of 0-0.2 for both axes. **Figures R4**, **R5** and **R6** are “zoomed-in” versions of **Additional File 1: Figures S2, S8, and S10**. Overall, BEDwARS estimates match the true proportions in magnitude better than the other methods as the scatter plots of BEDwARS are more aligned with the diagonal (identity) line. BayesPrism shows severe underestimation of beta cell type proportions by a factor of ~2 and overestimation of delta and gamma in all three datasets. Also, all methods except BEDwARS underestimate the acinar cell type proportions by at least 2 fold in Segerstolpe-H (**Figure R4**) and Segerstolpe-T2D (**Figure R5**).

In summary, BEDwARS estimates the rare cell type proportions better than the other methods in the benchmark on pancreatic datasets.

A

**D**

**Figure R1.  The histogram of cell type proportions for the pseudo-bulk mixtures used in the pancreas (A-C) and brain (D) benchmarks.**

| Dataset | Segerstolpe-H | Segerstolpe-T2D | Enge-H |
| --- | --- | --- | --- |
|  |  |  |  |
| Rare Ratio (%) | 38 | 44 | 17 |

**Table R1. The proportion of pseudo-bulk samples containing at least one rare cell type (cell type proportion <= 10%), in each pancreatic dataset.**

**Figure R2. BEDwARS performance comparison between pseudo-bulk samples with (“Has-Rare”) or without (“No-Rare”) rare cell types.**

**Figure R3.** **BayesPrism performance comparison between pseudo-bulk samples with (“Has-Rare”) or without (“No-rare”) rare cell types.**

**Figure R4. Quality of cell type proportion inference for small proportions (0-0.2) using Baron signature in deconvolving pseudo-bulk samples from Segerstolpe-H.**

**Figure R5. Quality of cell type proportion inference for small proportions (0-0.2) using Baron signature in deconvolving pseudo-bulk samples from Segerstolpe-T2D.**

**Figure R6. Quality of cell type proportion inference for small proportions (0-0.2) using Baron signature in deconvolving pseudo-bulk samples from Enge-H.**

**Comment 3:** The authors chose 3 methods for comparison based on a benchmark study in 2020, however there have been more methods published after 2020, such as BayesPrism (Nature Cancer, 2022). I would suggest the authors compare BEDwARS with more recent published methods.

**Response:**

We thank the reviewer for their suggestion. We had already compared BEDwARS with SCADIE (23), recently published in Genome Biology, for the deconvolution of Darmanis mixtures using NG and MM signatures. Please see the second-to-last paragraph (lines 492-500) of the Results subsection “**Robust deconvolution of brain transcriptomic profiles with BEDwARS”.** We did not perform the full benchmark on SCADIE as its performance is strongly dependent on the quality of estimated cell type proportions by *other* methods as initialization.

In response to the reviewer’s comment, we have now added BayesPrism to the evaluations in the revised manuscript. All the related figures and text in the manuscript and supplementary materials have been updated accordingly. Here is a summary of the performance comparison between BayesPrism and other methods.

Proportion estimation performance:

Pancreas data:

- Segerstolpe –T2D: When evaluated using Pearson Correlation Coefficient (PCC) between real and predicted proportions of a cell type (across all samples), BEDwARS and BayesPrism are better than others with noisy signatures; BEDwARS is more robust to noise than BayesPrism, with significant improvement over BayesPrism at NL-4, NL-5 (**Figure 2C**). In evaluations using RMSE or MAE (**Additional File 1: Figure S3C,D)** there is an even more clear advantage of BEDwARS over BayesPrism, at all noise levels and even in no-noise Baron signature.
- Segerstolpe – H: we see the same trends as above, with a clear advantage of BEDwARS over BayesPrism at noise levels NL-4, NL-5, NL-6 (PCC) (**Figure 2A**). **Additional File 1: Figure S3A,B** (MAE, RMSE) also reveals the same trend as above, i.e., a clear advantage of BEDwARS over BayesPrism at all noise levels and in the no-noise setting.
- Enge – H: same trends as above, with BEDwARS showing improved performance over BayesPrism (PCC difference >= 0.1) for NL-4, NL-5, NL-6 (**Figure 2E**). **Additional File 1: Figure S3E,F** (MAE, RMSE) reveals the same trend as above.

Brain data:

- IP signature: PCC reveals that BEDwARS and BayesPrism are comparable and significantly better than others at higher noise levels (NL-4, NL-5, NL-6) (**Figure 4A**). **Additional File 1: Figure S17A,B** (MAE, RMSE) also reveals the advantage of these two methods over others at higher noise levels.
- Other signatures also confirm that BEDwARS and BayesPrism are comparable (when evaluated using PCC), and for the more diverged signatures (NG, MM) they are clearly better than the other methods (**Figure 4C**). **Additional File 1: Figure S17C,D** (MAE, RMSE) shows that for the MM signature all methods are similar, for NG BEDwARS is substantially better than all other methods including BayesPrism (also see **Additional File 1: Figure S23**) and for CA (the least diverged signature) BayesPrism achieves a very strong performance, though other methods (including BEDwARS) also perform well (MAE, RMSE < 0.1).

Signature estimation performance:

Pancreas data:

- Segerstolpe – T2D (PCC): BEDwARS, RODEO/BEDwARS and RODEO/BayesPrism are similar, and are better than others at all noise levels (**Figure 3C**).
- Segerstolpe – H (PCC): BEDwARS, RODEO/BEDwARS and RODEO/BayesPrism are better than others at all noise levels; BEDwARS and RODEO/BEDwASRS show better PCC than RODEO/BayesPrism at higher noise levels (NL-4, NL-5 and NL-6) while BayesPrism is marginally better at lower noise levels (NL-1, NL-2, NL-3) (**Figure 3A**).
- Enge – H (PCC): BEDwARS, RODEO/BEDwARS and RODEO/BayesPrism are better than others at all noise levels; they are similar to each other except at the highest noise levels (NL-5, NL-6) where BEDwARS and RODEO/BEDwARS are clearly better (**Figure 3E**).

Brain data:

- Darmanis (PCC): all methods are similarly accurate, except at the highest noise levels (NL-5, NL-6) where RODEO/BEDwARS and RODEO/BayesPrism are better than others (**Figure 4B**).
- Other signatures (PCC): all methods are similarly accurate, though RODEO/BEDwARS and RODEO/BayesPrism have some advantage for the more diverged signatures NG, MM (**Figure 4D**).

We should point out that some RODEO runs failed when using estimated proportions from some methods including BayesPrism but not BEDwARS. In the results reported in the revised manuscript, we report the performance metrics based only on the successful, choosing to ignore the failed runs of RODEO/X, which would otherwise have produced a zero PCC. Below we show the effect of this allowance, comparing signature estimation PCC on the Segerstolpe-H data set when ignoring the failed runs (A) and when setting the evaluation metric (PCC) to zero in cases of failed RODEO runs (**Figure R7**). This shows that the performance advantage of BEDwARS over competing methods for signature estimation may in fact be greater than that reported in the manuscript (summarized above) if the failed RODEO runs were not ignored.

A

B

**Figure R7. Evaluation of cell type signature estimation by PCC criterion without (A) and with (B) zeroing out the correlation of any failed RODEO run with the estimated proportions from BayesPrism, FARDEEP, and CIBERSORT(x). None of the RODEO runs failed with BEDwARS estimated proportions. Thus, no difference is seen between A and B for BEDwARS.**

**Comment:** 4. What makes BEDwARS better performed than other tools and why? The authors should at least discuss in the manuscript.

**Response:**

In paragraph 4 of the Background section, we have provided an overview of the previous efforts in addressing the mismatch between cell type signature and bulk expression profiles. In paragraph 5, we have explained how BEDwARS is different from prior approaches. BEDwARS explicitly models the noisy signatures and allows for the true unknown cell type signature to deviate from the reference signature in a cell type specific manner which is also a key factor in distinguishing BEDwARS from BayesPrism. BayesPrism refines the estimation of cell type proportions by introducing perturbations to the single cell-derived reference, with a prior expectation that the *extent* of such perturbation is shared across cell types, while BEDwARS allows (a priori) for the perturbations to have cell type-specific extents. (Here, “extent” refers to standard deviation parameters in appropriate prior distributions.). Therefore, BEDwARS is more suited in cases where different cell types undergo perturbations with varying degrees, which is demonstrated in our benchmarking.

The above key difference between BEDwARS and BayesPrism is discussed in the Discussion section. We note that these two methods use completely different models, and it is challenging to distil all of their technical differences in simple language, and hope that the above passage will be helpful to the reader.

**Comment 5:** The time and memory consumption of each algorithm is also an issue that users are concerned about, and there is a lack of relevant analysis and comparison in the manuscript.

**Response:**

We compared the running time and memory requirements for CIBERSORTx, BayesPrism and BEDwARS in deconvolution of Segerstolpe-T2D pseudo-bulk samples using Baron signature (**Table R2**). Run time of BayesPrism is dependent on the number of CPU threads used. Two Intel(R) Xeon(R) CPU X5650 @ 2.67GHz were used for BayesPrism which was run with 20 or 5 CPU threads. CIBEERSORTx was run on one Intel(R) Xeon(R) CPU X5650 @ 2.67GHz.

We used one Tesla V100 GPU for BEDwARS which was run with 150 sampler chains. We should note that BEDwARS run time increases with the number of chains and depends on the number of steps for the random walk. Both parameters can be set by the user which is covered by a complete guide on the code repository on GitHub. Based on this comparison, BEDwARS takes longest to run; however, we believe that its running time can be improved (by exploiting strategies that guarantee faster convergence.)

**Table R2.** Comparison of the run time and memory requirements for deconvolution of Segerstolpe-T2D dataset with Baron signatures.

Details of computing the memory and time required for running BEDwARS are shared on the GitHub page. The analysis of run time and memory requirements has been added to the end of the Methods section of the paper and the table above has been added to the supplement.

**Comment 6:** Line 539-543, the authors claimed that "However, the proportions of these two individual cell types could not be accurately resolved due to the similarity in the signatures (see Additional File 1: Figure S33). Similar observations were made for the cilia-bearing cells (CBC) and intermediate (INTER) cell type proportions, with their sum matching between bulk-deconvolved and single cell data" This makes me concerned about the performance of BEDwARS in distinguishing similar cell types, which goes to my question 1.

**Response:**

We repeated the deconvolution results shown in Figure 5C (repeated here as **Figure R8B**), now with CIBERSORTx (**Figure R8A** below). CIBERSORTx is better than BEDwARS in that it estimates the INTER cell type to have higher proportion than CBC which matches the true relative abundance of these cell types in the Non-affected group. However, CIBERSORTx largely underestimates cortical neurons (CN) and overestimates neurons (NEU) proportions while the sum of inferred CN and NEU proportions roughly matches the sum of their true proportions. In other words, the reviewer’s concern about ambiguity in inferred proportions of “similar” cell types is valid and applies to other state-of-the-art deconvolution methods as well. Also, cell type 11, which is highly correlated with CN, neurons, PGC and astrocytes, (**Additional File 1: Figure S33**) has its proportion overestimated by CIBERSORTx, which is another sign of the method confusing similar cell types.

As discussed in the response to the first question and noted also by others (39), distinguishing highly similar cell types is challenging for current methods and is left for future work.

A.

B

**Figure R8. (A) Repeating the deconvolution of Figure 5C with CIBERSORTx. (B) Same as Figure 5C, i.e., deconvolution using BEDwARS.**

**Minor points:**
1. In Figure S3 and Figure S17. the citation of FARDEEP, CIBERSORT and CIBERSORTx is missing, simply wrote "[CITE]" in the manuscript.

**Response:** Thanks. This has been corrected.

2. Reference for Tanaka et al. in Figure S31, should be 26 instead of 25.

**Response:** The reference has been changed to 27 and corrected after updating the reference list.

**Reviewer 2:** Ghaffari et al. present a novel bulk gene expression deconvolution method BEDwARS using Bayesian inference. The authors took single-cell RNA-seq signature matrices as prior and estimated the cell type proportions and the true signature matrix for bulk simultaneously. With an emphasis on differences between reference signatures, Ghaffari et al. improved the performance of cell type proportions estimation even with single-cell reference. The authors demonstrate the accuracy of BEDwARS with simulations under different noise levels and two tissue types: pancreatic islet and brain. The authors also demonstrate the application of BEDwARS to characterize the cell-type specific regulomes of DPD-deficient patients with iPSC. 
The manuscript is clear, and the model is rigorous. However, I have a few concerns with the mode and general applicability of the tools. I have provided more details in my comments below:

**Response:** We thank the reviewer for their positive feedback and constructive criticism, and we hope that our point-by-point response addresses their concerns and improves the manuscript.

**Comment 1:** For reference-based deconvolution methods, such as CIBERSORT, MuSiC and Bisque, the deconvolution results for each bulk sample are independent. With the same single-cell reference, the cell type proportions estimated from bulk samples are (not) affected by other bulk samples. However, independence does not hold for BEDwARS. I have two questions:
**Comment 1a.** There is an underlying assumption: the bulk expression should come from the homogenous population. Because this homogenous population comes from one true signature matrix. Does the author explore the violation of the homogeneity of the population?

**Response:**

We thank the reviewer for their attention to the main assumption of the model and the suggested analysis. To study the performance of BEDwARS in deconvolution of heterogenous bulk population we constructed a third dataset (named “Segerstolpe-MIX”) consisting of 50 pseudo-bulk samples from Segerstolpe-H and 50 pseudo-bulk samples from Segerstolpe-T2D. We then performed deconvolution on Segerstolpe-MIX dataset using Baron signatures. **Figure R9A** shows the performance comparison by PCC and RMSE metrics averaged over the 50 Segesrtolpe-H pseudo-bulk samples that were deconvolved in homogenous (Segerstolpe-H) vs heterogeneous (Segerstolpe-MIX/H) settings. The same performance comparison is shown for Segerstolpe-T2D in **Figure R9B.** The deconvolution performance of Segerstolpe-H pseudo-bulk mixtures is similar for all cell types in heterogenous and homogeneous settings by both metrics, whereas the deconvolution of Segerstolpe-T2D pseudo-bulk mixtures is worsened in heterogenous setting for acinar and ductal cell types by both metrics. For beta and delta cell types, the deconvolution performance is affected as per RMSE metric only. In summary, BEDwARS performance is by and large robust to violation of the homogeneity assumption, as per our evaluations.

The observed occasional deterioration in performance is expected as the underlying assumption of BEDwARS is a shared signature among all pseudo-bulk samples. Accommodating for higher granularity of signature adjustment, e.g., sample-specific signatures, is a more challenging problem, which is left for future work. This has now been noted in the Discussion section. Also, the analysis performed on heterogenous bulk population has been added to the paper at lines 369-372.

A

B

**Figure R9. Deconvolution performance comparison between homogenous and heterogenous settings on Segerstolpe-H and Segerstolpe-T2D datasets.** In the heterogeneous setting the bulk samples provided to the deconvolution method are a mix of pseudo-bulk samples from Segerstolpe-H and Segerstolpe-T2D. In either setting, the reported accuracy is for pseudo-bulk samples from Segerstolpe-H only (panel A) or from Segerstolpe-T2D only (panel B).

**Comment 1b:** How do the sample sizes affect the accuracy of BEDwARS when the samples are from the homogenous population? Intuitively, the larger the sample size, the higher accuracy. How many samples at least do you recommend to achieve better or comparable accuracy with CIBERSORT, CIBERSORTx, and FARDEEP?

**Response:**

We thank the reviewer for the suggested analysis. To study the effect of sample size, we repeated deconvolution of Segerstolpe-T2D data set using Baron signature with 50 and 25 samples and compared the performance with sample size 100 (original analysis in the paper, **Figure 2B**), using averaged PCC and RMSE per cell type (**Figure R10**). The PCC is not affected by sample size. Also, except for gamma and beta cell types, the RMSE is not affected by sample size. This experiment suggests that at least 25 samples should be used in presence of six cell types for the deconvolution task. Generalizing from this, we thus recommend users to use datasets with the size at least four times the number of cell types to be deconvolved, as smaller sample sizes may not be well suited for the BEDwARS methodology. This analysis has been added to the paper at lines 373-375.

**Figure R10. Impact of sample size on the BEDwARS performance.** Deconvolution of Segerstolpe-T2D data set using Baron signature with three samples sizes 25, 50, and 100.

**Comment 2:** In the simulation, what do the estimated true signatures look like when they are estimated from different reference signatures? For example, with different noise levels, do the estimated true signature matrices look the same? Intuitively, the discrepancies between estimated true signature matrices and the true signature matrices are correlated with the noise level.

**Response:**

If we have interpreted the reviewer’s comment correctly, we did in fact examine this point in the original manuscript, computing the PCC and RMSE between estimated and true cell type signatures for all noise levels in the pancreatic and brain benchmarks. Please see **Figure 3A,C,E** and **Figure 4B** for signature PCC and **Additional File 1: Figure S15** for signature RMSE. Indeed, as the reviewer anticipated, with increasing noise level the PCC and RMSE get worse for all methods but BEDwARS (RODEO/BEDwARS) is more robust to the noise.

**Comment:** 3. On page 28, the formula at the bottom of the page. Rigorously, it would be better to write as Θ ̂ = argmax_Θ {Pr(Θ|X)}=argmax_Θ {Pr(X│Θ) Pr(Θ)}

**Response:**

Thanks. This has been changed to the rigorous format (page 29).

**2^nd^ round**

**Reviewer 1**

All my concerns were nicely addressed.
